# Optimization of parameters for semiempirical methods VI: more modifications to the NDDO approximations and re-optimization of parameters

**DOI:** 10.1007/s00894-012-1667-x

**Published:** 2012-11-28

**Authors:** James J. P. Stewart

**Affiliations:** Stewart Computational Chemistry, 15210 Paddington Circle, Colorado Springs, CO 80921 USA

**Keywords:** NDDO, Parameterization, PM6, Intermolecular interactions, PM7, Reaction barrier heights, Crystals, Solids, Transition metals, Semiempirical methods

## Abstract

Modern semiempirical methods are of sufficient accuracy when used in the modeling of molecules of the same type as used as reference data in the parameterization. Outside that subset, however, there is an abundance of evidence that these methods are of very limited utility. In an attempt to expand the range of applicability, a new method called PM7 has been developed. PM7 was parameterized using experimental and high-level ab initio reference data, augmented by a new type of reference data intended to better define the structure of parameter space. The resulting method was tested by modeling crystal structures and heats of formation of solids. Two changes were made to the set of approximations: a modification was made to improve the description of noncovalent interactions, and two minor errors in the NDDO formalism were rectified. Average unsigned errors (AUEs) in geometry and Δ*H*_f_ for PM7 were reduced relative to PM6; for simple gas-phase organic systems, the AUE in bond lengths decreased by about 5 % and the AUE in Δ*H*_f_ decreased by about 10 %; for organic solids, the AUE in Δ*H*_f_ dropped by 60 % and the reduction was 33.3 % for geometries. A two-step process (PM7-TS) for calculating the heights of activation barriers has been developed. Using PM7-TS, the AUE in the barrier heights for simple organic reactions was decreased from values of 12.6 kcal/mol^-1^ in PM6 and 10.8 kcal/mol^-1^ in PM7 to 3.8 kcal/mol^-1^. The origins of the errors in NDDO methods have been examined, and were found to be attributable to inadequate and inaccurate reference data. This conclusion provides insight into how these methods can be improved.

**Electronic supplementary material:**

The online version of this article (doi:10.1007/s00894-012-1667-x) contains supplementary material, which is available to authorized users.

## Introduction

In computational chemistry, semiempirical methods occupy a position intermediate between molecular mechanics and ab initio theory. By using approximations to avoid computationally intensive steps, and by using empirically determined parameters to obtain the best fit of predicted results to a training set of reference data, a method that is considerably faster than ab initio methods and considerably more versatile than molecular mechanics methods can be developed. Methods of this type—part quantum theory and part empirical—are known as semiempirical methods. One of the more robust families of such methods are the neglect of diatomic differential overlap (NDDO) methods [1, 2] first developed by Pople. Following the pioneering work of Dewar and Thiel in developing the modified neglect of differential overlap (MNDO) method [3, 4], several modifications were made to the NDDO formalism in attempts to increase accuracy and generality, among which the most popular are AM1 [5], PM3 [6, 7], PM6 [8], and RM1 [9].

As each new method became available, it exhibited some advantages over previous methods; thus, when PM6 was developed, the average unsigned error (AUE) in the calculated Δ*H*_f_ values for simple organic compounds decreased by about 30 % relative to PM3. These advantages made each new method more attractive than its predecessors for modeling chemical systems. Unfortunately, an inevitable drawback of new methods is that, with increased usage, new limitations or faults become apparent. Many of these faults were present in earlier methods but were hidden by more severe limitations in those methods, and only became evident when the masking effects of the earlier faults were eliminated.

The advantages and faults of PM6 were typical of this pattern. The increased accuracy of PM6 compared to PM3 made it the preferred NDDO method, but, with increased usage, various faults—some previously undetected and some introduced during the development of PM6—became apparent. Among the latter was the incorrect prediction that the Si–O–H system was linear, a fault that was not present in PM3. Other errors became apparent when attempts were made to model systems, in particular various simple solids, which were very different from the species used in the parameter optimization.

The objective of the work described in the present paper was to investigate the causes of some of the known errors in PM6, in the hope that they could then be corrected and the applicability of the NDDO methods to biochemical macromolecules and crystalline solids improved. Because of the importance of structure in solids, and because of the increasing use of these methods when modeling systems of biochemical interest, increased emphasis was placed on heats of formation and geometries, and less emphasis on electronic phenomena such as dipole moments and ionization potentials.

## Known faults in PM6

After PM6 was published, several types of errors were found. Many of these errors were of the type that could have been corrected, had they been detected during the development of the method. Among these was the reduced or missing repulsion between certain pairs of atoms, most importantly Na–Na, Br–N, Br–O, Br–Br, S–N, S–S, S–O, S–Cl, I–N, I–O, and I–I. Several other less important errors were found, all of which could have been eliminated had they been detected before the method was released.

Another type of error in PM6 was detected only after an attempt was made [10] to use PM6 to model crystal structures. This error was unique in that it had an insignificant effect on discrete species such as atoms, molecules, and ions, on polymers, and even on layer systems, but gave rise to an infinite error when applied to solids. A re-examination of the approximations allowed the origin of the error to be identified and a correction was made [10] to PM6; however, to avoid method proliferation, this correction was implemented only when PM6 was used in the modeling of crystalline solids.

A procedural fault was identified during the development of PM6. This fault was present in the earlier methods, but its importance only became apparent with the release of PM6. Despite the fact that great care was taken during the development of PM6, a process that took many years, most of the faults in it were not identified during the development phase; they were only detected after the method was published. As most of these faults could have been corrected if they had been detected before the method was completed, this phenomenon—readily correctable errors lying undetected until after the method was finalized, and then being discovered when the method was used “in the field”—should itself be regarded as a fault that should be corrected.

## Theory

### Further modifications to the NDDO formalism

#### Constraint on the value of the core–core interaction

**Fig. 1:**
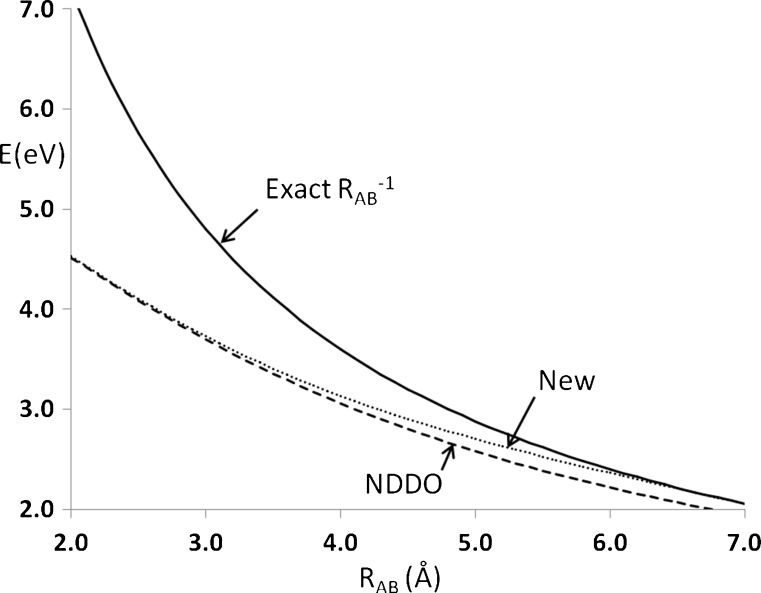
Energy versus distance for two unit point charges, showing the transition from conventional NDDO (*dashed line*), exact point charge repulsion (*solid line*), and the new approximation used in PM7 (*dotted line*)

Early NDDO methods were parameterized to reproduce properties of molecules. Various constraints were imposed in order to ensure physically realistic behavior. For example, it is essential that the nuclear–nuclear interaction energy converges asymptotically to the exact value as the interatomic separation increases. Constraints of this type were sufficient for discrete species, but when applied to crystalline systems, the early NDDO approximations were found to be inadequate [10], and additional constraints were required. In conventional NDDO methods, the rate at which different nuclear–nuclear interactions converged on the exact value as the interatomic distance increased differed depending on the specific atoms involved. This difference would obviously be very small (at 10 Å the difference would be chemically insignificant); however, when infinite sums of the type found in solids are involved, such small differences could become very large. In the specific case of solids, the error due to differing nuclear–nuclear interactions would be infinite [10]. A minor change was made to the NDDO formalism for solids to avoid this catastrophe and allow the electron–electron, electron–nuclear, and nuclear–nuclear terms to converge to exact values at separations greater than 5 Å. Having different approximations for the electrostatic interaction in discrete species and solids is obviously not ideal, so in the current work the solid-state form of the electrostatic interaction was used for both isolated species and for solids. To minimize the effect of this change on isolated species, the distance for completion of the transition to the exact value was increased to 7.0 Å, well beyond the covalent bonding distance. Any modification of this type would have a small but significant effect on solid-state properties, an effect that could be minimized by increasing the rate of transition to the exact point charge value. The rate of this transition could not be set arbitrarily high: if the rate of transition were too high, the electrostatic terms between atom pairs would become unrealistic—with increasing distance, the magnitudes of all electrostatic terms and their first derivatives would no longer decrease monotonically (a necessary condition for the interaction to be physically sensible). A compromise that satisfied these three requirements—minimizing changes to discrete species, eliminating infinite errors in solids, and ensuring realistic physical behavior—was achieved by replacing the two-electron two-center integral <*ss*|*ss*>, normally abbreviated to *γ*_AB_ (see Eq. 4 in [10]), with Eq. 1 for all interatomic separations *R* of less than 7.0 Å; up to that distance, a smooth transition from the NDDO approximation to the exact point charge expression was used, as shown in Fig. 1. In this expression, *G*_*A*_ is the two-electron one-center integral for atom A.

1 $$ {\gamma_{AB }}=\frac{1}{R}{e^{{-0.22{{{\left( {R-7} \right)}}^2}}}}+\left( {1-{e^{{-0.22{{{\left( {R-7} \right)}}^2}}}}} \right){{\left( {{R^2}+\frac{1}{4}{{{\left( {\frac{1}{{{G_A}}}+\frac{1}{{{G_B}}}} \right)}}^2}} \right)}^{-1/2 }}. $$

#### Constraint on the value of the electron–electron repulsion integral

A consequence of the previous constraint requiring the <*ss*|*ss*> term to converge to the exact solution at a finite distance was that the nuclear–nuclear and electron–nuclear terms also had to be modified in a similar way. Changes of this type are necessary in order to satisfy the requirement that there must be no net attraction or repulsion between any two well-separated neutral atoms. However, while examination of various atom pairs showed that this was true for two hydrogen atoms, there were small but finite forces present for all other atom pairs. These forces were traced to the one-center multipole terms, which increase or decrease the apparent distances between atoms. As also seen for the nuclear–nuclear and electron–nuclear terms, although the effect of individual perturbations of this type was small, the effect on solids would be infinite. The correction was also similar: a constant was added to the nine integrals of the type <*pp*|*pp*> so that the average value was exactly equal to that of the <*ss*|*ss*> integral.

Similar changes were made to the three integrals of type <*ss*|*pp*>, and, where relevant, to integrals of type <*ss*|*dd*>, <*pp*|*dd*>, and <*dd*|*dd*>. The result was an exact balance of electron–electron repulsion, electron–nuclear attraction, and nuclear–nuclear repulsion at all distances beyond 7 Å, ensuring that, at such distances, there was no net attraction or repulsion between neutral atoms.

A second modification was made to correct a spurious contribution to the energy of solids arising from hybrid orbitals or lone pairs. Two types exist: the *s-p* type, best exemplified by the lone pair in ammonia, and the *s-d* type, found in some transition metal complexes. Errors in interaction energies involving atoms separated by 10 Å are small compared to the error just described; however, in solids, all such infinite summations of even very small errors generate infinite errors. The correction made was to decrease the value of the hybrid NDDO integrals in a manner similar to that in Eq. 1 but without adding in any compensating term, with the result that the modified integrals converged to zero with increasing distance faster than the original NDDO integrals.

#### Energy of *p*-shell electrons in transition metals

There is a paucity of reliable thermochemical data on gas-phase transition metal compounds. In an attempt to augment the data, recourse was made to the Moore atomic energy level data [11–13]. These data provide information on the internal structures of individual atoms and ions. No problems were encountered for states involving only *s* or *d* electrons; however, during parameter optimization of the transition metals, a problem was encountered when data representing atomic energy levels involving *p* electrons were included. Initially, the resulting parameters appeared reasonable: as expected, the *p* populations of all transition metal atoms in all systems used in the parameterization were very small. Additionally, in most solids involving transition metals, the *p* population was again very small. However, in a limited number of systems, specifically some metallic elements, the *p* population increased considerably and the calculated heat of formation (Δ*H*_f_) became unrealistically negative.

This catastrophe was traced to one of the main features of NDDO theory: the neglect of differential overlap. In transition metals, the *p* atomic orbitals are highly diffuse, so the interaction of such orbitals with any nearby atom would be very small. This was the situation in all of the species used in the parameter optimization and in most solids, but was not the case in many of the pure crystalline elements. Two factors, one common to all solids and one unique to pure elements, combined to produce this nonsensical result. First, instead of the diffuse *p* orbital interacting with only a small number of other atoms, now there were a large number of atoms in the vicinity of any given metal atom. This would give rise to a large number of small one-electron energy terms that, when summed, would be significant. This would not necessarily present a problem in solid compounds because bond polarization would reduce the covalent character, but a unique feature of pure elements introduces a second factor. In most crystalline metal elements (manganese being the most dramatic exception), all atoms are in the same environment, so all chemical bonds are purely covalent; that is, the ionic bond character found in almost all other solids is absent. Atomic orbital overlap behaves like the one-electron energy term: although each individual overlap would be small, in solids there are a large number of such terms, and the sum would be significant. If atomic orbital overlap had been taken into account, then the energy term would (correctly) have been reduced in magnitude, but because such overlap terms are ignored in NDDO theory, the energy term is not reduced by the overlap, so the interatomic one-electron energy term would be much too large.

If the energy of a *p* atomic orbital in an atom were sufficiently high, the *p* population in all solids would necessarily be small, but in those cases where its energy was derived from experimental atomic energy level data [11–13], the *p* orbitals are competitive with the *s* and *d* orbitals for the available electrons. When the self-consistent field (SCF) equations are solved for transition metals using *p* orbital energies derived from atomic energy levels, some systems became excessively stabilized, resulting in the computational model becoming unrealistic.

Fortunately, the number of chemical systems that involve atoms in which both *p* and *d* electrons are involved in a chemical bond is vanishingly small. This fact suggests a way out of the impasse: by artificially raising the energy of the *p* orbitals in certain elements, thus removing them from participating in bonding, the excessive stabilization just described can be avoided. This effect could be readily achieved by adding appropriate excited-state reference data and by removing all experimentally derived atomic energy level data that include *p* electrons. Other than introducing some spectroscopic inaccuracies, no physical quantity would be compromised by this action—if the *p* electron population was artificially reduced to an insignificant value, the result would be the same as removing the elements’ *p* basis set.

#### Addition of dispersion and hydrogen bonds

When MNDO was first developed in 1977, it was rightly hailed as a major advance over earlier semiempirical methods such as MINDO/3 [14]. Part of the design of MNDO was that parameters were optimized to reproduce molecular properties, and this contributed to a large increase in the accuracy of prediction of heats of formation, geometries, etc. Unfortunately, soon after MNDO was completed, a severe error was found: an almost complete lack of intermolecular interactions such as van der Waals attractions and hydrogen bonds. Over the next few decades, many attempts were made to mimic these interactions by modifying the core–core interactions. These attempts resulted in only minor improvements, and even in the most recent formulation, PM6, the errors were still very large. Because of these failures, it is unlikely that any further efforts to modify the NDDO formalism would result in a significant decrease in this error.

With the advent of linear scaling techniques, semiempirical methods have become useful for modeling large biochemical systems such as DNA and proteins, particularly enzymes. In all such systems, intermolecular interactions, especially hydrogen bonding, play an essential role, so the failure of these methods to accurately reproduce intermolecular interactions seriously limits their applicability and casts doubt on the validity of any results obtained.

Recently, several post-SCF corrections have been proposed in attempts to correct the errors in PM6. Thus, Hobza’s group has proposed adding Jurečka et al.’s dispersion terms [15] and a hydrogen bond correction, as these are the two most important intermolecular interaction terms.

Testing was done using Hobza’s benchmark database S22 set [16]. As the name suggests, this was a set of 22 systems, each of which consisted of a two-molecule assembly in which the geometry had been optimized using high-level ab initio methods, and the energies obtained using CCSD(T)/CBS [17]. The interaction energy was calculated by subtracting the sum of the energies of the two components—calculated separately, but using the geometry that each component had in the assembly—from the energy of the assembly. As the authors noted, the resulting quantity is uniquely defined and would be useful when testing lower-level methods and as reference data for parameterizing semiempirical methods. Hobza’s databases thus have great value when determining the accuracy of theoretical methods and as a source of reference data. A minor point is that they do not, however, have physical significance, in that the geometries of the two components, calculated separately, were not at their respective energy minima.

Hobza’s first method, PM6-DH [18], reduced the mean unsigned error (MUE) in intermolecular interactions for the S22 set for PM6 from 3.17 kcal/mol^-1^ to 0.54 kcal/mol^-1^. The hydrogen bond term in PM6-DH was particularly simple, depending only on an interatomic distance, an angle, and the partial charges on the hydrogen and acceptor atoms involved. This dramatic improvement was followed quickly by PM6-DH2 [19], in which the hydrogen bond energy term was extended to include some torsion angles. This resulted in the MUE decreasing further to 0.36 kcal/mol^-1^, a value significantly less than the MUE of 0.7 kcal/mol^-1^ obtained [19] when B3LYP with dispersion correction was used.

On the other hand, because the main objective of semiempirical methods is to model chemical systems, the value of such methods depends only on how accurately they reproduce the physical system involved. For these methods, an essential step when calculating intermolecular interaction energies is to optimize geometries, and this operation requires that the forces acting on the atoms must be calculable. In semiempirical theory, the force or gradient calculation relies on the fact that when a self-consistent field exists, the energy of the system is irreducibly low (a restatement of the variational principle), and therefore the first derivative of the electron distribution with respect to the geometry must be exactly zero. Unfortunately, in both PM6-DH and PM6-DH2, the hydrogen bond energy is a function of the atomic partial charges on the hydrogen and acceptor atoms, which in turn is a function of the electron distribution. The hydrogen bond correction in these methods is calculated using the density distribution from the SCF calculation, and since the hydrogen bond correction alters the energy of the system but does not affect the electron distribution, the energy of the system is no longer irreducibly low. Obviously, the energy could be lowered by altering the electron distribution. When either PM6-DH or PM6-DH2 is used, an unavoidable consequence is that small errors are introduced into the calculation of the forces acting on the atoms. The effect is small but finite, and has the practical effect of making geometry optimization less efficient. More perniciously, the energy and force minima do not coincide, leading to the result that any resulting optimized geometry is ill-defined.

To ensure that the energy and force minima coincide, the energy of the hydrogen bond interaction must be made independent of the fractional atomic charge. This was investigated by Korth, who developed an elegant (i.e., simple) method, PM6-DH+ [20], which depended only on the geometry of the system, not on partial charges, thus ensuring compliance with the variational principle.

A problem with PM6-DH+ has recently been reported by Řezáč and Hobza [21] when linear hydrogen bonds are involved. This was reproduced during testing of PM6-DH+, in that the geometry optimization procedure sometimes failed when specific, very simple, hydrogen-bonded systems were involved. Interestingly, errors of this type were not found when more complicated systems were used. The cause of the fault [21] was confirmed to be the existence of a cusp (i.e., a discontinuous, albeit still single-valued, function) in the treatment of the hydrogen bond.

The ideas explored in PM6-DH2, PM6-DH+, and PM6-D3H4 [21] were used to construct an intermolecular interaction term for PM7. To prevent discontinuities, Korth’s damping functions were used, and, following Řezáč and Hobza’s warning, care was taken to avoid cusps. To ensure compliance with the variational principle, partial charges were not used. With these changes, all common hydrogen bonds could be modeled, the only exceptions being some very strong bonds of the type found in systems such as the Zundel ion, H_5_O_2_^+^, the dimer of formic acid, and in acid salts, e.g., NaH(CH_3_COO)_2_. All such bonds are characterized by very short O–H–O distances. In all these cases, the reduced O–O distance could be attributed to electronic phenomena: in the case of the charged species, to the large partial charges on the atoms, and in the case of neutral dimeric species, to cooperative effects in the eight-membered dicarboxylic acid ring. The requirements of the variational principle preclude adding a corrective term based on the partial charges; nevertheless, the extra stabilization that occurs in these species was too large to be ignored. The unusually short O–O distance, a feature obviously not present in ordinary (i.e., 4–6 kcal/mol^-1^) hydrogen bonds, was used as a basis for constructing a post-SCF correction. After a few trials, the form of the energy (*E*) correction adopted was that shown in Eq. 2, where *θ* is the O–H–O angle and *R*_*AB*_ is the O–O distance in Å.

2 $$ E=-2.5{{\left( {\cos \theta } \right)}^4}{e^{{-80{{{\left( {{R_{AB }}-2.67} \right)}}^2}}}}. $$

Another (less obvious) problem was encountered when Jurečka et al.’s dispersion terms were used to model solids. Dispersion interaction energy decreases as the sixth power of the interatomic distance, and Jurečka’s dispersion scheme is based on this behavior. No problems were encountered when Jurečka’s dispersion method was used to model discrete species, but the dispersion energy became unrealistically large when it was applied to solids. The extra dispersion energy was traced to the large number of small contributions from diatomic interactions that involve atoms separated by more than 4 Å, a situation that necessarily occurs in all solids. Although each interaction amounts to less than 10^−5^ kcal/mol^-1^, the large number of such interactions that occur in solids amounted to about 33 % of the total dispersion energy.

**Fig. 2:**
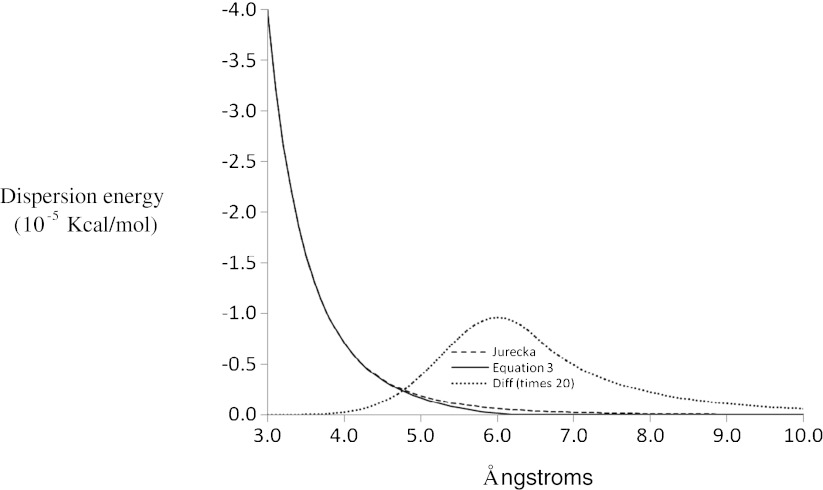
Effect of dampening function on correlation energy in solids

Classically, pairs of electrons correlate their instantaneous positions so as to minimize electron–electron repulsion, with electron pairs involving nearby atoms correlating their positions to a greater degree than when more distant atoms are involved (because the electron–electron interaction strengthens at smaller distances). For solids, the speculation can be made that the strong correlation arising from the presence of nearby atoms (i.e., atoms separated by 4 Å or less) has the effect of reducing the instantaneous correlation energy arising from more distant atom pairs. The postulated reduction in long-range correlation effects,* E*_disp_, in solids resulting from strong short-range correlation was implemented in PM7 by the addition of a damping function for all atom pairs separated by up to 6.5 Å (see Eq. 3 and Fig. 2); above 6.5 Å, all correlation terms were set to zero.

3 $$ E_{\text{disp}}^{\prime }={E_{\text{disp} }}\left( {1-{e^{{-{{{\left( {R-6.5} \right)}}^2}}}}} \right) $$

Both PM6-DH2 and PM6-DH+ were developed as post-SCF corrections to a PM6 calculation, and were designed to improve the accuracy of prediction of intermolecular interactions; they were not designed to improve the accuracy of prediction of Δ*H*_f_. Indeed, since the corrections typically amounted to several kilocalories per mole per hydrogen bond, and since the average signed error in PM6 was very small, the effect of adding in the dispersion and hydrogen bonding correction would be to make the calculated Δ*H*_f_ more negative, with the result that average signed and unsigned errors would both increased significantly. Therefore, these post-SCF corrections, while resulting in a large increase in the accuracy of prediction of intermolecular interaction energy, were unsuitable for use in predicting heats of formation. To avoid this, in the current work, the dispersion and hydrogen bond corrections were incorporated into the method before parameter optimization was performed, so PM7 is designed to reproduce both intermolecular interaction energies and heats of formation.

#### Reduction in the number of parameters

One consequence of the increase in computational power over the past few decades has been the ability to use more and more data in the training set and to allow more and more parameters to be optimized, so that, while in MNDO (developed in 1977) only a few tens of compounds were used in the training data set, and only seven parameters were optimized per element, in the more recent PM6 method (2007), several thousand compounds were used, and the number of parameters per element was increased dramatically.

While an increase in the number of parameters allows for increased flexibility, a potential problem arises in that the minimum of the error function in parameter space could become ill-defined (a well-defined minimum being one in which any significant motion in parameter space in any direction would be accompanied by a concomitant significant increase in the value of the error function). The presence of an ill-defined minimum is undesirable for many reasons, such as the inability to generate a unique set of parameters for a given training set, the presence of computational artifacts on molecular potential energy surfaces, and the violation of the rule known as Ockham’s razor, which can be re-worded to say that—all other conditions being equal—a simpler method is to be preferred over a more complicated one.

Although the parameter space minimum in MNDO was the most well defined of the NDDO methods, the MNDO method itself had a severe limitation in that π-stacking and hydrogen bonds were essentially nonexistent. To correct this fault, core–core Gaussian functions were added to atoms in PM3 and PM6 to mimic correlation or van der Waals effects. The resulting models included a much improved description of intermolecular interactions, but a direct and deleterious result of this change was to make the minimum in parameter space less well defined: a result which even a large increase in the number of reference data was unable to correct.

In PM7, correlation effects of the type used in PM3 and PM6 have been replaced by Jurečka’s dispersion term. This has allowed all of the atomic core–core Gaussian functions to be deleted with the exceptions of H, C, N, and O, as sufficient high-quality reference data are available to allow the Gaussian functions for these elements to be defined.

#### Modification to allow for UHF partial open shells

No further changes were made to the algebraic form of the Hamiltonian, but one minor change was made to the treatment of partial open-shell systems when unrestricted Hartree–Fock (UHF) methods were used. In many solids containing transition metal complexes with partially filled* d* shells, Jahn–Teller distortions are not observed due to rapid interconversion of the different forms—the dynamic Jahn–Teller effect. This experimentally observed phenomenon—solids having a higher symmetry than that expected by the Jahn–Teller theorem—can be reproduced in modeling by averaging the various states. Half-electron methods for MNDO have been developed for use with restricted Hartree–Fock wavefunctions [22, 23], but the application of these methods to solids would necessitate a solid-state configuration interaction (C.I.) calculation—a task that, at present, would be prohibitively expensive. By using a UHF wavefunction, the C.I. calculation could be avoided. In principle, UHF C.I. corrections could be made, but, as the energies involved would be very small, they can be ignored for the present purpose. The extension of half-electron methods to fractionally occupied degenerate manifolds of molecular orbitals in unrestricted Hartree–Fock methods, although obvious, has apparently not been reported. In part, this may have been due to the absence of any need to model systems of artificially high symmetry, a need that is pressing now that attempts are being made to model ambient temperature high-symmetry solids involving certain open-shell metal complexes.

Extension of the half-electron concept to UHF wavefunctions is straightforward. The only molecular orbitals (MOs) that need be considered are those with fractional orbital occupancy.

In these MOs, one spin—let it be beta for the sake of convenience—is either totally occupied or totally unoccupied. The alpha set of MOs, $\psi_i^{\alpha }=\sum\limits_{\lambda } {c_{{\lambda i}}^{\alpha}\phi \lambda }$, is given a fractional occupancy, *p*_*i*_, in the range 0 < *p* < 1. The density matrix, *P* ^*α*^, is then constructed as usual (Eq. 4), except that instead of all MO occupancies being zero or one, some would have fractional occupancy:

4 $$ P_{{\lambda \sigma}}^{\alpha }=\sum\limits_i {{p_i}c_{{\lambda i}}^{\alpha }c_{{\sigma i}}^{\alpha }}. $$

When several metal ions are present, they would all be treated similarly. For example, consider a complex in which the large unit cell contained 16 Ti^III^ ions, each with one alpha electron in a degenerate t_2g_ MO. Such a system would be treated as if there were 16 alpha electrons in a 48-fold degenerate set of MOs.

### Specific parameterization to reproduce barrier heights

Methods such as AM1, PM3, and PM6 have low accuracy in reproducing barrier heights for reactions. Various possible causes for this can be suggested: the restricted basis set used in semiempirical methods might preclude the development of a method that could simultaneously model both ground and transition states; subtle electronic phenomena might occur in the region of the transition state because of the lowered HOMO–LUMO gap; the almost complete absence of transition state systems in the parameterization training set might result in a lack of definition in that region of parameter space. For whatever reason, the low accuracy of these methods makes them unsuitable for modeling barrier heights. In an attempt to improve the accuracy of prediction of barrier heights, a specific parameterization has been attempted. Rather than investigate which of the three causes of error just described was responsible for the low accuracy, the focus of this parameterization was restricted to predicting barrier heights. This approach, while not desirable because it is not general, can be justified on pragmatic grounds: a method for predicting barrier heights with increased accuracy is likely to be useful when modeling chemical reactions.

The only methodological change required by this parameterization was to freeze all geometries (reactants, transition state, products) at their optimized PM7 structures.

### Reference data

#### Nature and structure of reference data and the parameter space

During various attempts to optimize parameters for semiempirical methods, i.e., MNDO (1977), AM1 (1985), PM3 (1989), PM6 (2007), and now PM7 (2012), the ideas of the nature and structure of reference data and parameter space have developed steadily. Over the same period, the methodology used to optimize the values of parameters has also evolved to the point where it can now be regarded as a stable and reliable process. It is therefore appropriate to describe the concepts currently used when considering issues involved in parameter optimization, in the hope that these will be of use to other method developers.

Before continuing, however, a semantic ambiguity must be resolved. Semiempirical methods involve parameters whose values are determined by minimizing an error function. When discussing parameter optimization, the two terms “values of the parameters” and simply “parameters” are used interchangeably. These terms are obviously different, but to avoid becoming pedantic or repetitious, the term “parameters” will be used below in place of “values of the parameters” when the meaning is obvious from the context.

Several types of reference data are used in parameter optimization, with heats of formation, geometries, dipoles, and ionization potentials being the most common. Before a reference datum can be used, it must first be rendered dimensionless; this is done by multiplying its value by an appropriate constant when the datum is fed into the computer program; for all subsequent operations that datum can be regarded as being a pure scalar quantity.

Associated with each datum is a chemical structure or chemical quantity. Examples of these would be a data set specifying a benzene molecule or the interaction of two water molecules.

The parameters can be regarded as forming a space of dimension equal to the number of parameters, with each point in parameter space being defined by the numerical values of the parameters at that point. Within parameter space, each datum can be characterized by an un-normalized vector of first derivatives, *d*_*i*I_, of the calculated value of the datum *i*, with respect to each parameter I. That is, quantities such as the structure of a benzene molecule or the energy of a hydrogen bond can be expressed as a simple vector. For the purposes of parameter optimization, this allows a reference datum to be re-defined as a two-quantity entity: a dimensionless scalar quantity representing the value of the datum, *c*_*i*_, and a vector representing the chemical nature of that reference datum, as shown in the following equation:

5 $$ {R_i}={c_i}+\sum\limits_{ \text{I}} {{d_{i { \text{I}} }}}. $$

##### Relationship between reference data

Just as a chemical can be characterized by properties such as reactivity, melting point, toxicity, etc., each computational reference datum can be regarded as having properties, admittedly completely alien to those of chemicals, but nevertheless very meaningful within the universe of parameter space. Some relationships between pairs of reference data are obvious; for instance, vectors for two reference data that have no elements in common, e.g., the bond length in hydrogen chloride and the Δ*H*_f_ of molecular nitrogen, are orthogonal in parameter space. The first vector would have nonzero derivatives for all parameters involving hydrogen or chlorine and zero derivatives for all other parameters, and the second vector would have nonzero derivatives only for parameters of nitrogen. Because they have no nonzero terms in common, the vectors are automatically orthogonal. The converse also holds: when two reference data represent the same property of two compounds that are similar (e.g., consecutive members of a homologous series), the angle between their vectors would be small. Intermediate between these extremes is the case of two reference data representing related but still different quantities, e.g., the Δ*H*_f_ of benzene and its C–C bond length, or the Δ*H*_f_ of acetic acid and the Δ*H*_f_ of glycerol. In cases like this, although the two vectors would not be orthogonal, they would still point in different directions in parameter space.

The relationship between the reference data vectors provides a mechanism for deciding which candidate reference data to use in parameter optimization. In order to allow the structure of a region of parameter space to be defined, it is both a necessary and a sufficient condition that there should be sufficient vectors that motion in any direction in parameter space could be represented by some linear combination of reference data vectors. A useful candidate datum, then, would be one whose vector pointed in a direction orthogonal or almost orthogonal to all existing vectors, as this would help define the structure of parameter space. On the other hand, a candidate datum whose vector could be constructed from a linear combination of existing vectors could not contribute towards satisfying the condition, and would therefore not be useful in defining parameter space. This can be expressed in chemical terms as the requirement that the compounds “should of course be chosen to cover as many different types of bonding situations as possible” [4].

Expressing reference data in terms of their behavior in parameter space results in a formalization of many of the ideas and guidelines used when developing methods. For example, it is obvious that if two different reference data exist for one chemical property, say the Δ*H*_f_ of water, they should not both be used. This can be re-stated in terms of vectors by saying that two identical vectors necessarily have identical properties in parameter space, so it would be impossible to fit both reference data (assuming they were different) using any conceivable set of parameters.

##### Properties of the minimum in parameter space

The objective of parameter optimization is to move across the parameter space surface so as to minimize the value of the error function *S*, the sum of squares of the errors in the predicted values *c*_*i*(calc)_ of the reference data *c*_*i*(ref)_:

6 $$ S=\sum\limits_i {{{{\left( {{c_{{i\left( {\text{ref}} \right)}}}-{c_{{i\left( {\text{calc}} \right)}}}} \right)}}^2}}. $$

The direction of motion Δ*P* required to reduce the error function can be calculated using the set of first derivatives of the error function (Eq. 7 below), as shown in Eq. 8:

7 $$ {g_{\text{I}}}=\frac{{\partial S}}{{\partial {\text{I}}}} $$

8 $$ \varDelta {P_{\text{I}}}=2\sum\limits_i {{g_{ {\text{I}} i }}\left( {{c_{{i\left( {ref} \right)}}}-{c_{{i\left( {calc} \right)}}}} \right)}. $$

At the parameter minimum, the local structure of parameter space (i.e., the rate of curvature in the various orthogonal directions) can be determined using the parameter Hessian, ***H***, the matrix of second derivatives of the error function with respect to the parameters:

9 $$ {H_{{\text{IJ}} }}=2\sum\limits_i {{g_{{\text{I}}i }}{g_{{\text{J}}i }}} $$

Diagonalization of the Hessian yields a set of eigenvalues which represent the force constants of the error function and their eigenvectors representing the corresponding normal modes of motion in parameter space.

The parameter hypersurface is built from contributions to the error function arising from all of the reference data used in the training set. As such, its construction is without doubt a complicated process. However, when viewed from a purely mathematical perspective, the parameter hypersurface is simple: it is merely a multidimensional, single-valued—albeit complicated—function. By regarding it as such, all the subtleties of semiempirical quantum chemistry theory can be ignored, and the behavior of the function as a purely mathematical construct can be investigated.

For a minimum to exist, two conditions must be satisfied: the gradient of the function with respect to all variables (parameters) must be zero, and all its eigenvalues must be either zero or positive. Note that it is not sufficient that all second derivatives of the function with respect to the variables are positive: indeed, from the fact that the error function is a sum of squares, these derivatives are obligate positive; it is the values of the eigenvalues that are important.

##### Properties of the parameter Hessian

Several properties of the Hessian are of interest. Given a Hessian of size *N*, if exactly one reference datum were used, there would be one positive eigenvalue and *N*−1 exactly zero eigenvalues. The eigenvector associated with the nonzero eigenvalue would then be the vector of the reference datum, normalized to unity. If more reference data were used, the number of nonzero eigenvalues would increase. The eigenvectors of the set of nonzero eigenvalues would then map out the directions along which motion would result in an increase in the error function. Motion in the direction of any eigenvector whose eigenvalue was precisely zero would not result in an increase in the value of the error function; nor would motion in any direction that could be represented by a linear combination of such eigenvectors. At this point, the mathematics is similar to that involved in solving a polynomial with *N* terms and an increasing number of independent equations. When there are *N* such independent equations, an exact solution becomes possible.

As alluded to above, a necessary and sufficient condition for the minimum on the parameter hypersurface to be a true minimum is that all eigenvalues of the associated Hessian must be positive. If any eigenvalue were to be exactly zero, then motion in the direction of the associated eigenvector would not result in an increase in the error function, and the corresponding parameter set would therefore not be unique: rather, it would be ill-defined. The presence of very small or exactly zero eigenvalues in the parameter Hessian thus violates Ockham’s razor in that it implies that there are more parameters than necessary. Unfortunately, it is difficult to identify and remove parameters responsible for zero eigenvalues, as the offending eigenvectors invariably involve significant contributions from many parameters. An alternative is to add to the training set reference data that would cause the zero eigenvalues to become positive. This might appear to be a challenging task too, but in practice it is quite straightforward. If there are no restrictions on the amount of reference data used to build the Hessian, it is sufficient to simply include large amounts of a wide range of reference data: if the data are sufficiently varied, positive contributions are made to all possible eigenvalues. Eigenvalues that were zero would become positive, and eigenvalues that are large would become even larger.

The objection might be made that if two parameters were completely dependent on each other, an exactly zero eigenvalue would necessarily exist. Such a situation would occur if, for example, all ionization potentials and all ionized states were omitted from the training set. In that case, the addition of a constant to all one-electron one-center integrals would have no influence on any results (heats of formation, dipole moments, and geometries would all be unaffected), but as soon as ionized states are included, the previously zero eigenvalue would become positive, and the arbitrary constant would be constrained by the optimization procedure. During the development of AM1 and PM3, multiple Gaussian functions were used in the construction of the core-core term. This gave rise to parameter dependence, resulting in methodological artifacts that were a source of frustration, as mentioned by Dewar during the AM1 parameter optimization of phosphorus: “The parameter hypersurface … is complex, having numerous local minima” [24–26]. At the time, this was attributed [26] to the parameters being “trapped in a ‘wrong’ minimum on the parameter hypersurface.”

To date, no general strategy has been developed to remove this dependence, although reducing the number of parameters obviously reduces the likelihood of parameter dependence. This is another re-statement of the well-known generalization that the number of parameters should be as small as possible.

From this description, it is obvious that an analysis of the eigenvalues and eigenvectors of the parameter Hessian provides a wealth of information on the nature of the minimum. It indicates the relevance of the reference data and its limitations, and provides a guide on how to improve it. The significance of the parameters can also be deduced—whether a parameter is redundant and therefore should be removed, or whether it is essential to the theoretical framework.

#### Not enough reference data to allow a wide range of chemistry

In earlier semiempirical methods, all entries in the training and survey reference data sets were limited to experimental or high-level ab initio quantities such as heats of formation, bond lengths and angles, etc., of discrete molecular species. Early data sets, such as those used when parameterizing and surveying MNDO, were assembled directly from the original published literature. Building these data sets was a time-consuming process which placed severe limitations on the types of parameter optimizations that could be done. Indeed, in the early years, parameterizing single elements was considered a significant achievement that warranted reporting in the form of a journal publication. Most elements had only a small number of parameters, typically 7–16 for a main-group element, and the available data were sufficient for the resulting methods (MNDO, AM1, and PM3) to be of useful accuracy and predictive power. With the advent of compendia such as the NIST WebBook [27] and the Cambridge Structural Database (CSD) [28], large amounts of critically reviewed (i.e., accurate) reference data became available. Constructing training sets became easier. In the next NDDO method, PM6, diatomic parameters were re-introduced. Diatomic parameters had been used in MINDO/3, but were abandoned, in part because of the proliferation of parameters that would occur as the number of elements increased. All technical difficulties involved with using diatomic parameters have now been overcome due to the development of faster processors and less expensive memory. What has not been solved was the problem of assembling enough reference data to allow all pairs of diatomic parameters to be defined, and in PM6 one consequence of this lack of definition was a method that has proven useful when applied to species of the type used in the parameterization but was essentially useless for all other systems. In an attempt to increase the range of applicability of PM6, over 9000 reference data were used in the training set, with most data being obtained from compendia of the type already mentioned. Despite this large number of data—only about 800 were used in the parameterization of PM3, and less than 100 in the parameterization of MNDO—the vast majority of possible diatomic interactions remained undefined.

The CSD contains over 600,000 small molecule crystal structures, with about half of them being suitable for use as reference data. This is an obvious source of geometric reference data, particularly for species in which intermolecular interactions are sufficiently small that crystal packing forces do not cause any significant distortion. In such systems, the geometry of a molecule would be a good approximation to the geometry of an isolated gas-phase molecule. While useful for parameterizing atom pairs that normally involve covalent bonds, such data are not suitable for atom pairs that involve strong noncovalent bonds of the type that exists between, say, sodium and oxygen in, say, Na_2_SO_4_.

Another large repository is the Inorganic Crystal Structure Database (ICSD) [29]. In contrast to the CSD, the ICSD consists largely of species that involve strong noncovalent interactions. That solids of the type found in the ICSD are important can be easily illustrated by considering the alkali metal ions. In molecular species, these are invariably monodentate, normally forming a single strong bond that is partially ionic and partially covalent; a simple example would be the isolated NaCl molecule. In biological systems, however, these ions are highly labile, with the covalent component being essentially insignificant. This situation is precisely reproduced in various crystal structures; thus, in sodium hydrogen phosphate, Na_2_HPO_4_, each sodium ion forms ionic bonds with several oxygen atoms, and each [HPO_4_]^2−^ ion is subject to the electrostatic environment of several sodium ions. Examination of the crystal structure shows that there is no species that could be identified as a discrete sodium hydrogen phosphate entity. Additionally, in crystalline Na_2_HPO_4_, there are strong hydrogen bonds of the type found in many biochemical systems: such bonds could not be realistically modeled using isolated systems.

The main characteristic of crystal structures that is absent from discrete species is the existence of long-range (2–4 Å) interactions. At such distances, purely quantum mechanical terms (i.e., covalent interactions) are decreasing rapidly with increasing distance, and the dominant terms are the electrostatic and steric interactions. As the electrostatic terms are well represented within the set of NDDO approximations, the only term present in crystals for which there is no equivalent in discrete species is the nonbonding steric interaction. This feature of crystal structures makes them a source of valuable reference data for defining the values of parameters. Unfortunately, it is impractical to use entire crystals in the parameterization because of the computational effort involved, and isolated species are de facto not good models of the types of solids in the ICSD because of the presence of strong electrostatic interactions.

#### Use of proxy functions to represent noncovalent interactions

Within current NDDO theory, the nonbonding steric term is represented by Voityuk’s core–core interaction approximation [30]:

10 $$ {E_n}\left( {A,B} \right)={Z_A}{Z_B}\left\langle {{s_A}{s_A}\left| {{s_B}{s_B}} \right.} \right\rangle \left( {1+{x_{AB }}{e^{{-{\alpha_{AB }}{R_{AB }}}}}} \right). $$

In this expression, *Z*_*A*_ and *Z*_*B*_ are the core charges on atoms *A* and *B*, respectively, <*s*_*A*_ *s*_*A*_|*s*_*B*_ *s*_*B*_> is the two-center two-electron repulsion integral involving *s* orbitals, *R*_*AB*_ is the interatomic distance, and *x*_*AB*_ and *α*_*AB*_ are parameters. Because the steric repulsion is defined using two parameters per diatomic pair, this term has great flexibility, and methods that use it are capable of improved accuracy, as illustrated by PM6. However, when methods that were parameterized using molecular species were used to model crystal structures, the results were often unsatisfactory. This was a direct consequence of the absence of any reference data that specifically represented interatomic interactions outside the covalent bonding distance. That is, the flexibility of Voityuk’s diatomic function, which was so beneficial when used to model molecules, was deleterious when applied to solids.

The fact that reference data representing steric behavior were not used in earlier methods obviously does not preclude their use in later methods. Indeed, by adding a very primitive reference function, specifically the steric term at a given distance, the fault just described can be completely corrected. The form of the reference datum is shown in the following equation, where the steric contribution *E*_*AB*_ at an interatomic separation of *R*_*AB*_ Å is set to *c* kcal/mol^-1^:

11 $$ {E_{AB }}\left( {{R_{AB }}} \right)=c. $$

Such a term does not involve the wavefunction, and its use can be restricted to optimizing the values of diatomic parameters, without reference to any other quantity. The value of *R*_*AB*_ can be set to the value of the smallest nonbonding interaction in a representative crystal, typically 2–4 Å, and the value of *c* would be set such that the correct interatomic distance was reproduced when the geometry of the crystal was modeled. Regrettably, at the present time, this step appears to be more art than science. Because noncovalent interatomic separations in crystal structures can be specified using functions of the type *E*_*AB*_, these functions could be regarded as proxy functions for crystals; functions used in parameterization that represent geometric quantities that are specific to solids.

The idea that a single reference datum of the type shown in Eq. 11 can be used to define two core–core repulsion parameters might superficially appear inadequate. However, other reference data, specifically data representing interactions at covalent distances, would also be available to the parameter optimization process. This allows the proxy function to be used solely for the purpose of defining the potential in the nonbonding region. Consider, for example, the noncovalent interactions in lithium borohydride, LiBH_4_. In this ionic solid, the lithium–boron interatomic distance is determined by two forces: the electrostatic attraction of the lithium cation and the borohydride anion, and the steric repulsion of the lithium by the boron and hydrogen atoms. At the energy minimum, these two forces are exactly equal and opposite in direction. If the calculated value of the Li–B distance were too small, increasing the value of *c* for Li–H or Li–B would increase the steric or repulsive force, with the result that the interatomic separation would increase.

No simple molecular systems exist for many diatomic pairs. When that happens, the two parameters in Eq. 10 can be defined by using two reference data of the type shown in Eq. 11: one at a relatively small distance, 1.5–2.5 Å, and one at a larger distance, 2.5–3.5 Å.

For most solids, increasing the value of the steric term for interactions outside the covalent bonding region would result in an increase in the interatomic separation. While this result might appear to be obvious, there is one group of solids for which an increase in steric repulsion in this region results in a *decrease* in interatomic distance. This apparently paradoxical effect occurs in only a few solids, mainly the pure elements, and only in those solids in which discrete molecules are absent (i.e., most of the metals and a small number of binary compounds). The difference between this set and all other solids is the presence in such solids of non-nearest neighbors at unusually small distances. If the steric term for such 1–3 interactions were to be reduced from the optimum value, then the 1–3 interaction energy would also be reduced, and it might appear as if the energy of the system would be reduced by decreasing the 1–3 interatomic separation. However, in these systems, an inevitable consequence of any reduction in the 1–3 steric energy is a concomitant increase in the gradient of the nearest-neighbor interaction energy. As 1–2 interactions are obviously much greater than 1–3, the increased gradient for the 1–2 interaction results in a larger decrease in energy when the 1–2 separation is increased. The net effect is that, for those elements in which covalent bonds form an infinite lattice, the effect of changing the steric energy term for 1–3 interactions is opposite to that seen in all other systems.

#### Reference data for PM7-TS

Reference data for barrier heights were obtained from collections of high-level calculations only [31–36]. As an objective of this work is to improve the accuracy of prediction of barrier heights in biochemical systems, specifically enzyme-catalyzed reactions, the set of reactions was restricted to those involving simple organic bond-making–bond-breaking, and electronic phenomena were restricted to the singlet surface only. Because the number of reference data was quite small (only data on 97 barrier heights were used), reference data for simple ground-state compounds that were predicted with good accuracy by PM7 were added to the training set in an attempt to increase the definition of the minimum in parameter space.

#### Additional constraints

For several elements there were insufficient reference data to allow the minimum in parameter space to be defined, and extra reference data were generated in an attempt to constrain the parameters to “reasonable” values. The most common type of constraint involved defining energies of isolated high-energy atoms and ions, so that, for example, during the parameterization of silver, in addition to the known excited states (^8^D_u_, ^8^P_g_, and ^8^F_u_), the hypothetical state of Ag^3+^ (with the configuration 5*s* ^2^4*d* ^6^) was used. Although such data have no chemical significance, they were found to be essential for defining the minimum in parameter space. The presence of these data is obviously undesirable, and as soon as sufficient data become available to allow the minimum to be defined, these artificial constraints should be removed.

### Parameter optimization

Parameter optimization was performed in a manner similar to that used in the development of the PM6 method. Starting with a training set of reference data consisting of systems containing the core elements H, C, N, and O only, and using the values of parameters from PM6, an initial set of optimized parameters were obtained. These parameters were then used as the starting point for parameter optimization of the other organic elements: F, P, S, Cl, Br, and I.

All other elements were then optimized, one at a time, while freezing the parameters for the organic elements. After the parameters were optimized, a validation test was done using solids containing the appropriate elements. Depending on the results of the test, the values of the proxy functions were changed, and the parameter optimization re-run.

Each individual parameter optimization procedure was relatively rapid, requiring only 1–2 CPU hours. However, since the process had to be repeated many times, the whole process of parameter optimization for the 70 elements took several CPU years of effort.

## Results

Average unsigned errors (AUEs) in the heats of formation for various sets of compounds and solids are presented in Tables 1 and 2, and the AUEs in geometries are shown in Tables 3 and 4. For compounds, the entries in each set contain all of the elements listed. For solids, each set contains only the elements listed (e.g., all sets contain hydrocarbons). Geometries of solids are expressed in arbitrary units as a function of errors in bond lengths and of the overall crystal structure.

**Table 1:** Comparison of the average unsigned errors in Δ*H*_f_ (in kcal/mol^-1^) for sets of compounds calculated via PM6 and PM7

Compounds containing	AUE PM6	AUE PM7	No. in set
H, C	4.75	4.13	307
H, C, N	3.66	3.30	210
H, C, O	4.26	3.62	370
H, C, N, O	4.61	4.47	231
H, C, P	5.16	3.98	9
H, C, O, P	6.74	8.71	24
H, C, S	3.56	2.45	59
H, C, F	3.91	4.66	32
H, C, Cl	2.46	2.36	43
H, C, Br	2.11	1.80	16
H, C, I	2.16	1.64	27
All simple organics	4.42	4.01	1366
All elements	8.38	12.03^a^	4369

^a^See text for explanation

**Table 2:** Comparison of the average unsigned errors in Δ*H*_f_ (in kcal/mol^-1^) for sets of solids calculated via PM6 and PM7

Solids containing	AUE PM6	AUE PM7	No. in set
H, C, N, O	15.95	5.66	76
H, C, N, O, F,P,S, Cl, Br I	19.77	6.98	93
H, Li, C, N, O, Na,P, S, K, Rb, Cs	20.15	8.63	98
All elements	557.66^a^	14.86	676

^a^See text for explanation

**Table 3:** Comparison of the average unsigned errors in bond lengths (in Å) for sets of compounds calculated via PM6 and PM7

Compounds containing	AUE PM6	AUE PM7	No. in set
H, C	0.016	0.015	76
H, C, N	0.017	0.016	92
H, C, O	0.022	0.019	93
H, C, N, O	0.022	0.019	109
H, C, P	0.017	0.017	80
H, C, O, P	0.023	0.020	97
H, C, S	0.016	0.015	81
H, C, F	0.017	0.026	77
H, C, Cl	0.016	0.015	77
H, C, Br	0.017	0.016	77
H, C, I	0.017	0.015	77
All elements	0.098^a^	0.087	5035

^a^See text for explanation

**Table 4:** Comparison of the average unsigned errors in the geometries of solids (in arbitrary units) calculated via PM6 and PM7

Solids containing	AUE PM6	AUE PM7	No. in set
H, C, N, O	16.80	13.66	209
H, C, N, O, F,P,S, Cl, Br I	18.71	14.78	311
H, Li, C, N, O, Na, P, S, K, Rb, Cs	19.13	15.17	299
All elements	34.02	22.43	2207

From Table 1, it is clear that, for simple organic compounds, PM7 is about 10 % more accurate than PM6 (which, in turn, is significantly more accurate than earlier methods, such as PM3 and AM1) in predicting heats of formation. For the same set, the AUE for B3LYP using 6-31 G(d) is 5.14 kcal/mol^-1^, and for HF 6-31 G(d) it is 7.34 kcal/mol^-1^. In both sets of ab initio calculations, contributions from zero-point and internal energies were ignored, the assumption being that these contributions would be accounted for in the root-mean-square fit for atom additivity. Based on these statistics, PM7 represents a significant improvement in the prediction of heats of formation over earlier NDDO-type methods, and is also significantly more accurate than popular ab initio methods when applied to simple organic species.

This improvement is not sustained when the set of all compounds is used. Instead, there was a dramatic increase in AUE in going from PM6 (8.4 kcal/mol^-1^) to PM7 (12.0 kcal/mol^-1^), strongly suggesting that PM7 would be less accurate than PM6 when applied to general chemistry. When this result was first observed, it was completely unexpected, because both methods were parameterized using similar procedures, and an investigation into its origin was immediately started.

During the parameterization of PM7, when the increased AUE in heats of formation first became apparent, an attempt was made to reduce the AUE by removing the proxy functions from the training set. While this did result in a reduction of the AUE for molecular species, it was also accompanied by a large increase in AUE for both heats of formation and geometries of solids. Interestingly, it quickly became apparent that there was a clear pattern to the errors: in the regions of chemistry where reference data were scarce, the predictions of PM7 were unusually inaccurate when proxy functions were present but of the normal accuracy when the proxy functions were absent. Given that these functions were designed to allow solids to be predicted with increased accuracy, and given that most reference data on solids were of unquestioned accuracy (at least, unquestioned compared to the errors in semiempirical methods), the unavoidable conclusion was that much of the reference data used in the parameterization of PM7 had to be inaccurate. This same reference data had been used to parameterize PM6, but as shown in Table 2, PM6 did not perform well when applied to solids.

Possible sources of inaccuracy in reference data can be identified. Where experimental data are scarce, the reason for the scarcity might also be the reason for the inaccuracy: experimental difficulties. Many theoretically predicted data, particularly for species involving very heavy elements, were incompatible with experimentally determined properties of solids. There is a high probability that the ab initio method used (DFT PW91 6-31 G(d)) was simply unsuitable. What is incontrovertible is that many of the reference data for discrete species were incompatible with the known properties of solids.

One option considered was to delete all reference data that was suspected to be faulty (i.e., all data that the embryonic PM7 was unable to reproduce with good accuracy). This option was rejected on the grounds that, if it were implemented, it would then be impossible to determine the accuracy of any resulting method. An unavoidable consequence of this decision was the need to report that, for Δ*H*_f_, the AUE of PM7 for discrete species is considerably higher than that of PM6, despite the fact that there are questions about the accuracy of some reference data. This is obviously an unsatisfactory situation, and one that should be rectified as soon as possible.

Average unsigned errors in dipole moments increased in going from PM6 (0.82 D) to PM7 (1.08 D), as did ionization potentials (0.50 eV for PM6 rising to 0.55 for PM7). This disappointing result was probably caused by the decreased emphasis on electronic properties, but the possibility cannot be dismissed that it was another indirect consequence of the use of proxy functions.

### Solids

Crystalline solids provide a good test of the ability of a semiempirical method to model real chemical systems. Several hundred experimentally determined heats of formation exist for a wide range of solids, and many hundreds of thousands of X-ray structures have been deposited in readily accessible collections. This provides a wealth of reference data with which to compare calculated results. Unlike most gas-phase species studied by computational chemistry methods, solids represent real, tangible, chemicals of the type used in an experimental laboratory. Also, unlike gas-phase species, a very wide range of chemical environments are found in solids. Several of these, such as hydrogen bonds, π-stacking, and salt bridges, are found in biochemical macromolecules, but any attempt to focus on modeling these environments in macromolecules is made difficult by the sheer size of these species. In comparison, solids are relatively simple, and, because there are so many solids available, a solid that illustrates a particular biochemical structure of interest can always be selected. Finally, solids are excluded from being used in parameter optimization, as the computational cost of including even one solid is still prohibitive. Solids thus provide a wide range of chemical environments for testing the accuracy and predictive power of computational chemistry modeling methods.

**Fig. 3:**
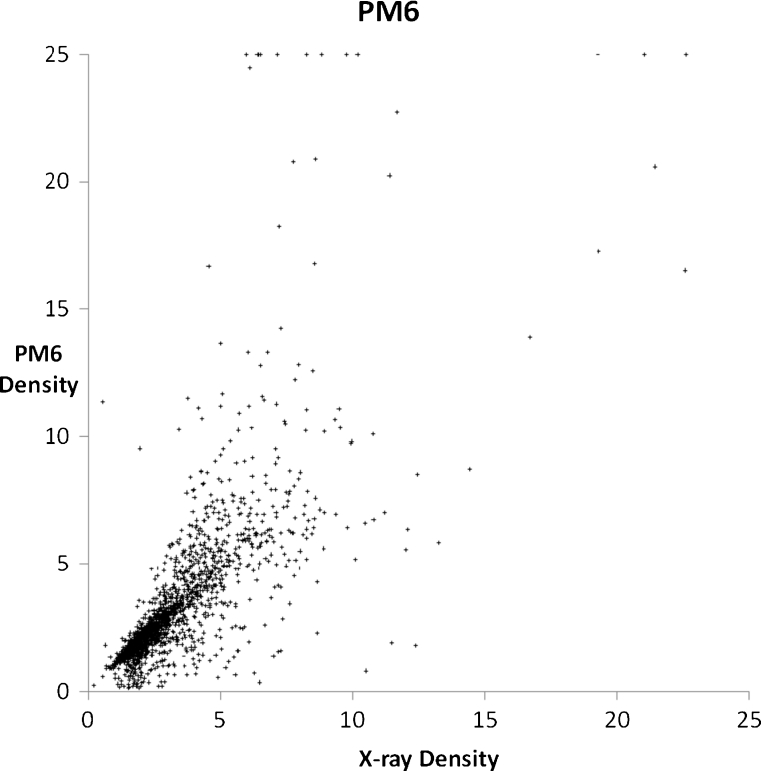
Comparison of PM6-predicted densities of 2,194 solids with their corresponding experimental values (g cm^−3^). Calculated densities that were greater than 25 were reset to 25

**Fig. 4:**
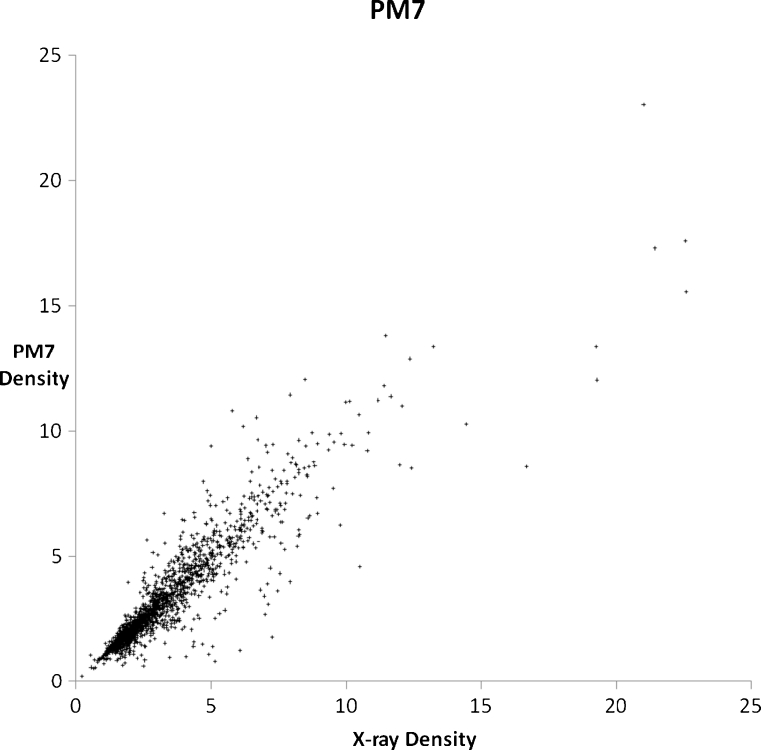
Comparison of PM7-predicted densities of 2,194 solids with their corresponding experimental values (g cm^−3^)

**Fig. 5:**
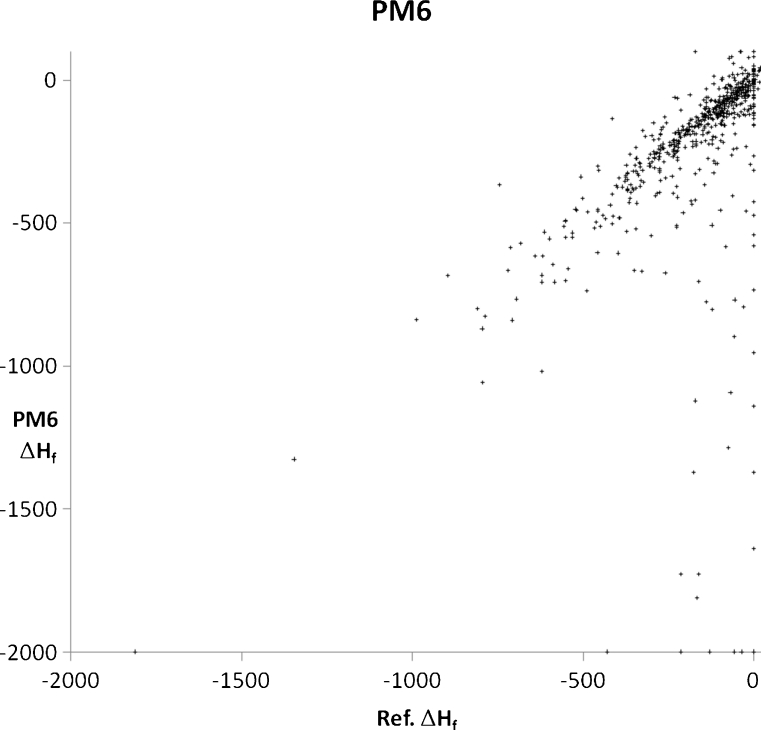
Comparison of PM6-predicted heats of formation of 676 solids with their corresponding experimental values (kcal/mol^-1^). Several solids, particularly elements, have large negative Δ*H*_f_ values, giving rise to the artifact at 0.0 kcal/mol

**Fig. 6:**
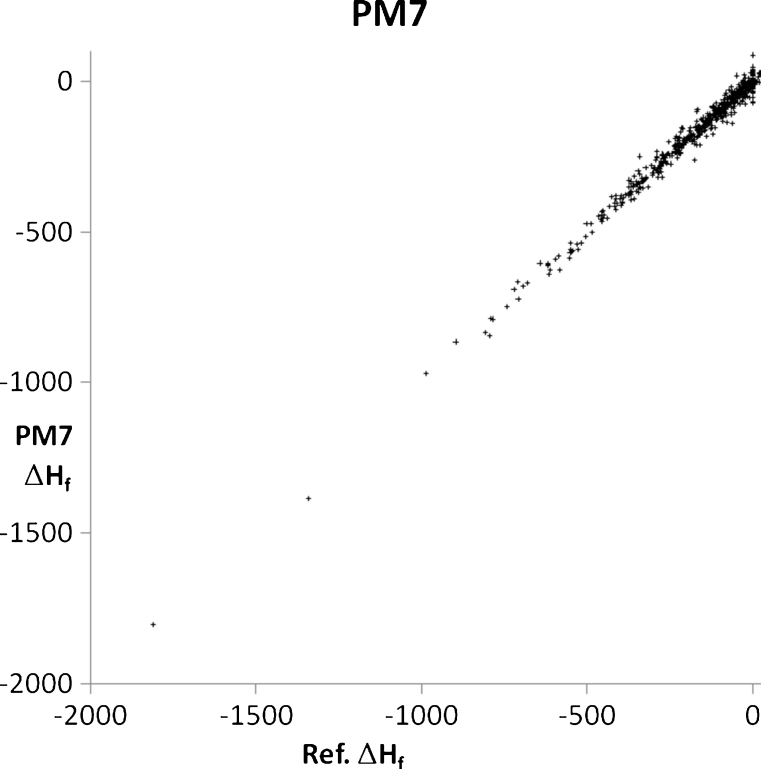
Comparison of PM7-predicted heats of formation of 676 solids with their corresponding experimental values (kcal/mol^-1^)

A set of 2,194 solids were modeled using PM6 and PM7, with each solid chosen in order to illustrate or examine some facet of chemistry. A general comparison of the accuracy of prediction of the properties of solids using PM6 and PM7 can be obtained from graphs of calculated and experimental densities (Figs. 3 and 4) and heats of formation (Figs. 5 and 6). Geometries predicted by PM7 represent a significant improvement over PM6, as reflected in the increased accuracy of density prediction, with the AUE for PM7 being 0.396 g cm^−3^, compared to 0.923 g cm^−3^ for PM6. A simple comparison of the AUE for Δ*H*_f_ between PM7 (14.8 kcal/mol^-1^) and PM6 (557.7 kcal/mol^-1^) would be misleading in that a small number of PM6 errors were extremely large. A better comparison would be the median unsigned error; this is 10.77 kcal/mol^-1^ for PM7 and 29.71 kcal/mol^-1^ for PM6.

#### Organic compounds

Most crystalline organic compounds are composed of discrete moieties. These range from simple structures (neopentane, adamantane), where only weak van der Waals forces hold the molecules together, through hydrogen and π-bonded systems (sucrose, anthracene, guanine), to ionic species (Na_2_CO_3_, the amino acids, and complexes such as [(N(CH_3_)_4_)]_2_[Pt^IV^Cl_6_]). This feature of organic compounds makes them especially useful for testing methods, particularly regarding how well they model intermolecular interactions.

Errors in the geometries of simple organic solids predicted using PM7 were about 26 % smaller than those predicted using PM6. A larger improvement was observed in the predicted heats of formation, with the AUE for PM7 being about 8.5 kcal/mol^-1^, and the AUE was 20.0 kcal/mol^-1^ for PM6. Part of this could be attributed to the changes in the approximations and to improved parameter optimization, but almost certainly the most important contribution was the improved representation of intermolecular interactions.

#### Dispersion and hydrogen bonds

Organic chemistry is dominated by the chemistry of molecules, and the nature of the noncovalent interactions between molecules is of great importance. Obtaining accurate reference data from experiment is difficult, and for that reason recourse has been made to the results of theoretical predictions. Two benchmark databases, S22 [16] and S66 [37], have been developed for testing computational methods. These databases contain the results of very high-level ab initio methods, and can be considered definitive: that is, the entries in the databases can be used to construct reference data for use in training sets and in surveys to determine the accuracy of semiempirical methods. Both databases report intermolecular interaction energies and geometries for pairs of simple organic compounds. The S22 database consists of 22 examples of typical noncovalent interactions that illustrate dispersion effects, hydrogen bonding, and mixed interactions. The S66 database is similar, but with increased emphasis on interactions of the type typically found in biochemistry. All of the entries in the S22 database, and none of the S66 entries, were used in the training sets. Errors in intermolecular interaction energies for various methods for the S22 set are shown in Table 5, and those for the S66 set are shown in Table 6.

**Table 5:** Intermolecular interactions for the S22 set (kcal/mol^-1^)

System	Ref.	Errors in				
	CCSD(T)/CBS	PM7	PM6	PM6-DH+	PM6-DH2	B3LYP
01. Ammonia dimer	−3.17	−1.18	0.87	0.01	−0.03	−1.40
02. Water dimer	−5.02	0.11	1.08	−1.45	0.12	−2.30
03. Formic acid dimer	−18.61	0.65	7.47	0.87	−0.04	−2.81
04. Formamide dimer	−15.96	−0.67	3.41	−1.89	0.10	−2.60
05. Uracil HB	−20.47	1.45	7.15	1.11	−0.73	−1.10
06. Pyridoxine aminopyridine	−16.71	−0.46	6.73	−0.07	0.36	−1.22
07. Adenine thymine WC	−16.37	0.66	7.31	0.50	−0.08	−0.96
08. Methane dimer	−0.53	0.17	0.47	0.08	0.08	0.74
09. Ethylene dimer	−1.51	0.44	1.11	0.45	0.45	1.05
10. Benzene methane	−1.50	−0.29	1.03	0.11	0.11	1.70
11. Benzene dimer stack	−2.73	−1.46	2.86	−0.84	−0.84	5.07
12. Pyrazine dimer	−4.42	−1.25	2.61	−0.92	−0.92	5.25
13. Uracil dimer stack	−9.88	1.30	5.42	0.47	0.44	5.94
14. Indole benzene stack	−5.22	−0.73	5.29	0.17	0.17	8.14
15. Adenine thymine stack	−12.23	0.92	7.29	0.57	0.54	10.03
16. Ethene ethyne	−1.53	0.54	0.98	0.58	0.58	−0.12
17. Benzene water	−3.28	0.51	1.00	0.10	0.10	0.62
18. Benzene ammonia	−2.35	−0.54	0.82	−0.19	−0.19	1.27
19. Benzene HCN	−4.46	1.41	2.48	1.47	1.47	1.38
20. Benzene dimer T	−2.74	−0.56	1.99	0.15	0.15	2.74
21. Indole benzene T	−5.73	−0.25	3.33	0.80	0.80	3.25
22. Phenol dimer	−7.05	0.79	3.67	−0.01	−0.01	0.92

**Table 6:** Intermolecular interactions for the S66 set (kcal/mol^-1^)

System	CCSD(T)/CBS value	Errors in				
		PM7	PM6	PM6-DH2	PM6-DH+	B3LYP
01. Water–water	−4.92	0.04	1.01	0.04	−1.55	−2.21
02. Water–methanol	−5.59	0.56	1.36	−1.23	−1.36	−1.50
03. Water–MeNH_2_	−6.91	0.10	2.86	0.62	−0.21	−1.48
04. Water–peptide	−8.10	0.46	1.82	0.08	−1.02	−1.06
05. MeOH–MeOH	−5.76	1.13	2.27	−0.51	−0.71	−1.44
06. MeOH–MeNH_2_	−7.55	1.51	4.46	1.83	0.91	−1.20
07. MeOH–peptide	−8.23	1.44	3.31	1.28	0.30	−1.65
08. MeOH–water	−5.01	0.72	1.82	0.75	−0.89	−2.16
09. MeNH_2_–MeOH	−3.06	−1.52	0.77	−0.92	−2.09	−0.57
10. MeNH_2_–MeNH_2_	−4.16	−1.22	2.32	0.85	−0.12	−0.02
11. MeNH_2–_peptide	−5.42	−0.77	1.57	0.07	−0.23	0.14
12. MeNH_2_–water	−7.27	0.79	3.42	1.29	0.73	−1.22
13. Peptide–MeOH	−6.19	−0.29	1.97	−0.20	0.16	−0.49
14. Peptide–MeNH_2_	−7.45	−1.91	3.28	0.58	−0.05	−0.41
15. Peptide–peptide	−8.63	−0.83	2.72	0.20	−0.68	0.25
16. Peptide–water	−5.12	−0.99	1.28	0.31	−0.24	−1.54
17. Uracil–uracil (BP)	−17.18	1.12	5.84	−1.72	−0.21	−1.29
18. Water–pyridine	−6.86	0.73	3.73	2.34	0.47	−0.51
19. MeOH–pyridine	−7.41	1.87	5.23	3.53	1.53	−0.37
20. AcOH–AcOH	−19.09	1.02	7.99	−0.19	1.34	−2.79
21. AnNH_2_–AcNH_2_	−16.27	−0.41	3.89	0.18	−1.58	−2.23
22. AcOH–uracil	−19.49	1.58	7.46	−0.50	1.26	−1.97
23. AcNH_2_–uracil	−19.19	0.31	5.14	−0.29	−0.64	−1.68
24. Benzene–benzene (π–π)	−2.82	−1.55	2.86	−0.65	−0.65	4.44
25. Pyridine–pyridine (π–π)	−3.90	−1.36	2.87	−0.72	−0.72	4.62
26. Uracil–uracil (π–π)	−9.83	1.35	5.46	0.45	0.48	6.11
27. Benzene–pyridine (π–π)	−3.44	−1.42	2.88	−0.68	−0.68	4.54
28. Benzene–uracil (π–π)	−5.71	0.20	4.08	−0.28	−0.28	5.85
29. Pyridine–uracil (π–π)	−6.82	−0.03	3.58	−0.68	−0.68	5.76
30. Benzene–ethylene	−1.43	−0.69	1.55	−0.45	−0.45	2.57
31. Uracil–ethylene	−3.38	0.65	2.34	0.14	0.14	2.85
32. Uracil–ethyne	−3.74	1.40	2.66	0.85	0.85	2.59
33. Pyridine–ethylene	−1.87	−0.43	1.63	−0.34	−0.34	2.57
34. Pentane–pentane	−3.78	−0.28	3.14	0.72	0.72	5.10
35. Neopentane–pentane	−2.61	−0.85	1.92	0.13	0.13	3.32
36. Neopentane–neopentane	−1.78	−0.99	1.23	−0.21	−0.21	2.06
37. Cyclopentane–neopentane	−2.40	−0.93	1.71	0.03	0.03	3.11
38. Cyclopentane–cyclopentane	−3.00	−0.43	2.61	0.67	0.67	3.59
39. Benzene–cyclopentane	−3.58	−0.62	3.04	0.56	0.56	3.96
40. Benzene–neopentane	−2.90	−0.99	2.19	0.19	0.19	3.11
41. Uracil–pentane	−4.85	−0.24	3.06	−0.11	−0.11	4.83
42. Uracil–cyclopentane	−4.14	−0.33	2.90	0.05	0.05	4.40
43. Uracil–neopentane	−3.71	0.13	2.66	0.30	0.30	3.52
44. Ethylene–pentane	−2.01	−0.13	1.55	0.28	0.28	2.44
45. Ethyne–pentane	−1.75	0.07	1.46	0.27	0.27	1.57
46. Peptide–pentane	−4.26	0.49	3.00	0.60	0.60	3.94
47. Benzene–benzene (TS)	−2.88	−0.33	2.07	0.22	0.22	2.74
48. Pyridine–pyridine (TS)	−3.54	0.20	2.31	0.53	0.53	2.53
49. Benzene–pyridine (TS)	−3.33	−0.17	2.17	0.32	0.32	2.69
50. Benzene–ethyne (CH–π)	−2.87	0.79	1.86	0.86	0.86	1.47
51. Ethyne–ethyne (TS)	−1.52	0.71	1.06	0.73	0.73	−0.16
52. Benzene–AcOH (OH–π)	−4.36	0.91	3.11	0.99	0.99	3.91
53. Benzene–AcNH_2_ (NH–π)	−3.28	−0.41	2.76	0.64	0.64	3.56
54. Benzene–water (OH–π)	−4.19	1.38	0.90	−0.47	−0.47	−0.35
55. Benzene–MeOH (OH–π)	−4.71	1.37	1.87	0.96	0.96	1.55
56. Benzene–MeNH_2_ (NH–π)	−3.23	−0.34	1.81	0.09	0.09	2.28
57. Benzene–peptide (NH–π)	−5.28	−0.09	3.00	0.56	0.56	3.17
58. Pyridine–pyridine (CH–N)	−4.15	0.55	1.64	0.71	0.71	0.79
59. Ethyne–water	−2.85	1.39	1.09	0.91	0.91	−1.14
60. Ethyne–AcOH (OH–π)	−4.87	2.56	3.04	2.47	2.47	−0.77
61. Pentane–AcOH	−2.91	0.15	1.59	−0.23	−0.23	2.28
62. Pentane–AcNH_2_	−3.53	0.08	1.99	−0.01	−0.01	2.27
63. Benzene–AcOH	−3.80	−0.15	1.23	−0.30	−0.30	0.60
64. Peptide–ethylene	−3.00	0.63	1.74	0.47	0.47	1.25
65. Pyridine–ethyne	−3.99	2.22	2.74	2.24	2.24	−0.49
66. MeNH_2_–pyridine	−3.97	−0.33	2.68	1.12	1.00	1.80

A survey (see Table 7) of the AUEs for the complexes in the S22 and S66 sets for various methods gave the unexpected result that the errors in PM7 are greater than those of PM6-DH2 and PM6-DH+. There is no obvious reason for this, although one possibility is that the parameters used when modeling intermolecular interactions in PM7 would also be used to minimize errors elsewhere: in both PM6-DH2 and PM6-DH+, the same parameters were only used to reproduce these interactions. Notwithstanding this result, it should be noted that the three methods PM7, PM6-DH2, and PM6-DH+ all have AUEs less than half that of B3LYP.

**Table 7:** Average unsigned errors for the S22 and S66 datasets (kcal/mol^-1^)

Set	PM7	PM6-DH2	PM6-DH+	PM6	B3LYP
S22	0.74	0.38	0.58	3.38	2.75
S66	0.78	0.66	0.64	2.68	2.29

After PM6 was released, a severe error was found in that many noncovalent interactions involving the halogens Cl, Br, and I were far too strong, and their interatomic separations were far too short. Řezáč and Hobza rectified this error by adding a post-SCF correction [38] to the PM6 method. As with S22 and S66 for PM6-DH2, they used the results of very high-level methods as a source of reference data [38] to optimize the parameters in the post-SCF correction. The same data were also used during the development of PM7, which meant that most of the errors in the halogens were corrected; the only significant error is an underestimated Cl–Cl repulsion.

#### Ions involving hydrogen and oxygen

Several forms of protonated water complexes occur in crystals, ranging from the simple hydroxonium ion, [H_3_O]^+^, found in systems such as HClO_4_.H_2_O and several 18-crown-6 complexes, through the Zundel ion, [H_5_O_2_]^+^, in, e.g., HCl.(H_2_O)_2_ and H_2_SO_4_.(H_2_O)_4_, to the Eigen ion, [H_9_O_4_]^+^, in, e.g., HCl.(H_2_O)_6_. By contrast, the anion [OH]^−^ almost always occurs as the simple hydroxide ion; lattice systems involving bridging hydrogen atoms of the type found in CsOH.(H_2_O) are relatively rare.

In a survey of 28 solids containing ions involving hydrogen and oxygen, 27 geometries were predicted with higher accuracy by PM7 than PM6; the single exception being hydroxonium 10-crown-6 clathrate hexafluorotantalate, CSD entry SINSEO, and even in this case, the local environment of the hydroxonium was predicted more accurately by PM7.

The structures of all three protonated water complexes were reproduced by PM7; in contrast, PM6 failed to reproduce the more fragile Eigen ion in hydrogen chloride hexahydrate.

#### Hydrocarbons

Solid hydrocarbons are a good test of purely dispersive interactions. In general, both PM6 and PM7 model gas-phase hydrocarbons with good accuracy, so comparison of the predicted heats of formation of their solids with experiment should give an estimate of the accuracy associated with the modeling of dispersive interactions. Results for a set of solid hydrocarbons are shown in Table 8. For this set, the AUE for PM7 is 5.96 kcal/mol^-1^, and for PM6 it is 22.21 kcal/mol^-1^. The increased accuracy of PM7 over PM6 can be attributed to the dispersion terms developed by Jurečka et al. [15]. Examination of Table 8 shows no obvious pattern in the errors in PM7; systems in which π-stacking might be considered important are reproduced with similar accuracy to those where π-stacking is absent, such as adamantane. This is in agreement with the recent recommendation [39] regarding “π-stacking” and “π–π interactions” that “these terms are misleading and should no longer be used.”

**Table 8:** PM7 and PM6 errors in the heats of formation of solid hydrocarbons

Hydrocarbon	Ref.	PM7	PM7 error	PM6	PM6 error
Naphthalene (NAPHTA32)	18.8	24.0	5.2	35.9	17.1
Adamantane (ADAMAN08)	−46.0	−45.7	0.3	−35.8	10.2
Biphenyl (BIPHEN04)	23.5	26.5	3.0	43.2	19.7
Hexamethylbenzene (HMBENZ04)	−38.8	−48.5	−9.7	−28.2	10.6
Fluorene (FLUREN02)	21.6	22.7	1.1	42.3	20.7
Anthracene (ANTCEN14)	30.0	34.5	4.5	52.8	22.8
Phenanthrene (PHENAN08)	26.2	30.0	3.8	48.3	22.1
9,10-Dihydroanthracene (DITBOX)	15.9	13.0	−2.9	30.4	14.5
*Trans*-stilbene (TSTILB03)	31.8	24.2	−7.6	54.6	22.8
Diadamantane (CONGRS)	−57.8	−52.5	5.3	−43.0	14.8
Cyclotetradecane (CYTDEC)	−88.9	−95.1	−6.2	−68.0	20.9
Fluoranthene (FLUANT02)	45.5	41.4	−4.1	71.5	26.0
Pyrene (PYRENE02)	29.9	32.8	2.9	56.2	26.3
Benzo[*c*]phenanthrene (BZPHAN01)	44.1	32.5	−11.6	69.6	25.5
Chrysene (CRYSEN01)	34.7	34.1	−0.6	62.8	28.1
Tetracene (TETCEN01)	49.4	36.1	−13.3	72.7	23.3
Triphenylene (TRIPHE12)	35.9	28.6	−7.3	63.7	27.8
Dibenzanthracene (SANQII)	42.7	38.6	−4.1	80.1	37.4
Coronene (CORONE)	36.4	33.7	−2.7	77.3	40.9
Dibenzo(g,p)chrysene (TEBNAP)	90.6	67.6	−23.0	103.2	12.6

#### Other strong hydrogen bonds

An exceptionally strong hydrogen bond can form when a proton bonds to two anions so that the three-moiety assembly has a net negative charge. This type of system occurs in acid salts and related systems. In some solids, such as ammonium hydrogen benzoate and sodium hydrogen acetate, the hydrogen atom is essentially bridging; in others, such as potassium hydrogen acetate and potassium hydrogen sulfate, the hydrogen atom is asymmetrically disposed. Whether a hydrogen atom is symmetrically or asymmetrically disposed between the two oxygen atoms in the bond depends on a delicate balance of energies, and therefore provides a sensitive test of hydrogen bonding. For most systems of this type, both PM6 and PM7 reproduce the observed structure, but while PM6 and PM7 both correctly predict the symmetric structure of sodium hydrogen acetate, only PM7 correctly predicts the asymmetric structure of potassium hydrogen acetate.

**Fig. 7:**
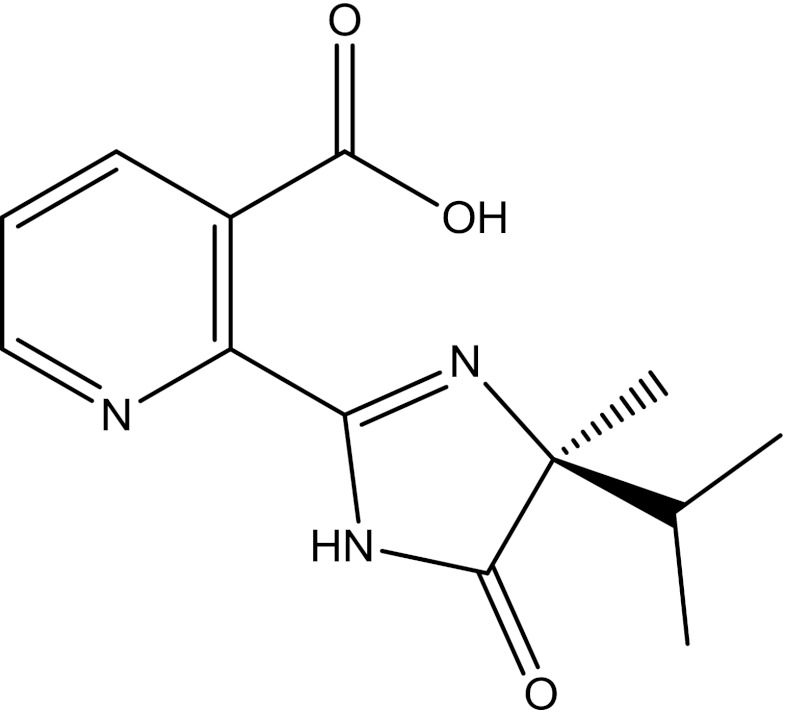
2-(2-(3-Carboxypyridyl))-4-isopropyl-4-methyl-5-oxo-imidazole

Still another type of strong hydrogen bond is found in 2-(2-(3-carboxypyridyl))-4-isopropyl-4-methyl-5-oxo-imidazole [40] (Fig. 7). In the solid state, this compound contains an internal N…H…O hydrogen bond between the carboxylic acid group and a nitrogen atom on the imidazole ring. If it is viewed as a neutral species, then the exceptionally short (2.465 Å) N–O distance would be difficult to explain, but if viewed as a zwitterion containing a cationic imidazolium ion and an anionic carboxylate group, then the close contact could be rationalized in terms of electrostatics. The exceptionally short bond is reproduced when the solid is modeled, with PM7 giving 2.508 Å and PM6 giving 2.517 Å. Interestingly, when the gas-phase system is modeled, both PM6 and PM7 predict the N–O distance to be nearer to that found in the more common, neutral, hydrogen bonds, strongly suggesting that the zwitterionic nature is only manifested when the cooperative effect of the crystal environment is present.

#### Halogen bonds

Average unsigned errors for intermolecular interactions for 20 complexes involving Cl, Br, or I noncovalently bonded to O, N, or a π-system were 2.46 kcal/mol^-1^ (PM6), 1.63 (PM7), and 0.76 (PM6-DH2X). As also seen with the S22 and S66 datasets, the PM7 AUE is greater than that of PM6-DH2X, but now a definite reason for this can be given: the post-method correction in PM6-DH2X was not made in PM7 because the cause of the severe error in PM6 (an absence of reference data involving Br–N and other interactions in the training set) was largely corrected in PM7. Obviously, a further reduction in error could be achieved by applying a specific correction of the type used in PM6-DH2X.

#### Energies of sublimation

Benchmark databases such as S22 and S66 are a good source of reference data for intermolecular interactions involving two molecules. These data are useful both in training sets and in calibration. Another measure of intermolecular interactions is the experimentally determined energies of sublimation; that is, the difference in energy of a chemical in the crystal and gas phases. In its simplest form, this quantity represents the stabilization energy arising from a molecule’s environment; however, other, more complicated phenomena are involved in several species, such as the change from the zwitterionic form of amino acids in the crystal phase to the neutral (i.e., unionized) form in the gas phase.

A comparison of calculated and experimentally determined heats of sublimation is presented in Table 9. Most of the experimental data were abstracted from the Chickos and Acree collection [41]. The error in the PM7 prediction of the heat of sublimation of one solid, hexachlorobenzene, was particularly large (27.5 kcal/mol^-1^); this was traced to an insufficiently large Cl–Cl repulsion. An attempt to correct this specific fault in PM7 failed, and at the present time this should be regarded as a known, correctable, error in PM7. Even with this single severe fault, the AUE for PM7 (5.0 kcal/mol^-1^) was still less than half that for PM6 (10.3 kcal/mol^-1^).

**Table 9:** Comparison of PM6 and PM7 with reference heats of sublimation

Chemical	Ref.	PM7	PM7	PM6	PM6
	Δ*H*_s_	Δ*H*_s_	Error	Δ*H*_s_	Error
Iodine	14.9	13.8	−1.1	48.7	33.8
Water	11.9	20.9	9.0	10.3	−1.6
Carbon dioxide	6.2	4.5	−1.7	1.9	−4.3
Iodoform	16.7	18.1	1.4	34.3	17.6
Methane	2.3	3.1	0.8	0.6	−1.7
Urea	21.7	29.2	7.5	17.5	−4.2
Oxalic acid	23.1	31.0	7.9	19.5	−3.6
Glycine	32.6	36.9	4.3	29.7	−2.9
Alanine	31.8	34.7	2.9	32.6	0.8
Propane	6.8	8.9	2.1	2.2	−4.6
Maleic anhydride	16.7	18.5	1.8	10.5	−6.2
Uracil	31.3	35.4	4.1	19.6	−11.7
2-Aminopyridine	18.3	26.9	8.6	9.2	−9.1
3-Aminopyridine	19.3	21.9	2.6	9.5	−9.8
4-Aminopyridine	20.8	26.3	5.5	10.4	−10.4
Thymine	32.0	37.9	5.9	17.9	−14.1
Methionine	39.2	42.0	2.8	28.2	−11.0
*n*-Pentane	10.0	13.5	3.5	1.9	−8.1
Hexachlorobenzene	21.6	49.1	27.5	2.5	−19.1
Hexafluorobenzene	11.9	16.9	5.0	0.3	−11.6
2,4,6 Tribromoaniline	24.1	35.3	11.2	52.5	28.4
Benzene	10.8	7.8	−3.0	3.2	−7.6
4-Nitroaniline	24.1	29.9	5.8	16.4	−7.7
2-Aminophenol	23.2	18.9	−4.3	17.6	−5.6
3-Aminophenol	24.4	27.3	2.9	10.7	−13.7
4-Aminophenol	24.8	32.6	7.8	19.3	−5.5
Cyclohexane	5.2	11.2	6.0	2.1	−3.1
4-Fluorobenzoic acid	21.8	25.7	3.9	12.0	−9.8
4-Chlorobenzoic acid	24.6	32.7	8.1	13.7	−10.9
Benzoic acid	21.9	26.2	4.3	10.0	−11.9
Tyrosine	52.3	52.2	−0.1	33.9	−18.4
Naphthalene	17.3	15.7	−1.6	4.2	−13.1
Ferrocene	17.5	16.0	−1.5	15.3	−2.2
Adamantane	14.1	17.1	3.0	2.6	−11.5
Hexamethylbenzene	20.3	23.9	3.6	4.3	−16.0
Pyrene	24.0	29.7	5.7	5.2	−18.8

Earlier reports had suggested [10, 42] that PM6 predicted zwitterions to be too stable. The results presented here for the heats of sublimation of simple amino acids suggest that this error was not as serious as first thought.

#### Co-crystal energies

An estimate of the accuracy of prediction of relative intermolecular interaction energies is provided by a comparison of the calculated heats of formation of co-crystals and their precursors (Table 10). In order for a co-crystal to exist it must be more stable (i.e., it must have a lower Δ*H*_f_) than its precursors. In a survey of 21 known co-crystals, PM7 predicted that all but two were more stable than their precursors, whereas PM6 predicted that all but five were more stable.

**Table 10:** Energies of co-crystals

Co-crystal	PM7 Δ*H*_f_ of co-crystal	PM7 Δ*H*_f_ of precursors	PM7 energy of co-crystal	PM6 Δ*H*_f_ of co-crystal	PM6 Δ*H*_f_ of precursors	PM6 energy of co-crystal
4,4′-Biphenol bis(caprolactam) (KEWZUI)	−273.2	−253.1	−20.1	−207.7	−207.2	−0.5
Bis(pyridinium) oxalate oxalic acid (DEFCUM)	−376.5	−364.2	−12.3	−326.7	−294.2	−32.5
Cinnamic acid 3-nitrobenzamide (OPUSOI)	−152.9	−134.9	−18.0	−106.3	−102.7	−3.6
Carbamazepine isonicotinamide (LOFKIB01)	−61.8	−61.2	−0.6	−7.6	−8.7	1.1
Resorcinol bis(caprolactam) (QQQGHM01)	−269.2	−261.4	−7.8	−231.2	−230.6	−0.6
4-(Dimethylamino)pyridinium hydrogen phthalate (GUKVOY)	−189.7	−186.7	−3.0	−150.3	−141.8	−8.5
Oxalic acid dihydrate (OXACDH26)	−350.1	−354.7	4.6	−318.3	−305.0	−13.3
Bis(4-(dimethylamino)pyridinium) fumarate fumaric acid (GUKWIT)	−391.0	−388.4	−2.6	−332.5	−307.6	−24.9
Bis(4-(dimethylamino)pyridinium) terephthalate (GUKWAL)	−186.2	−187.3	1.1	−138.7	−123.1	−15.6
Dipyridinium bis(hydrogen oxalate) oxalic acid (DUVLUB)	−570.9	−561.5	−9.4	−506.7	−470.0	−36.7
Succinic acid bis(urea) (VEJXAJ)	−404.1	−385.2	−18.9	−347.0	−343.1	−3.9
4-(Dimethylamino)pyridinium hydrogen maleate (GUKVUE)	−190.3	−182.3	−8.0	−165.0	−148.7	−16.3
Tris(4,4′-biphenol) bis(2-aminopyridine) (KEXBAR)	−265.0	−260.5	−4.5	−124.1	−124.6	0.5
Bis(pyridine) fumaric acid (GUKWOZ)	−167.2	−165.9	−1.3	−124.8	−119.3	−5.5
Malonic acid bis(isonicotinamide) (ULAWEJ)	−301.3	−297.8	−3.5	−252.0	−244.1	−7.9
Aspirin carbamazepine (TAZRAO)	−205.7	−205.6	−0.1	−151.1	−152.0	0.9
Isonicotinamide 3-hydroxybenzoic acid (LUNMEM)	−193.3	−181.4	−11.9	−145.6	−147.8	2.2
Cyanuric acid–melamine (QACSUI)	−199.3	−180.8	−18.5	−130.9	−123.9	−7.0
4-Aminobenzoic acid 4-nitroaniline (RILJEB)	−125.5	−120.2	−5.3	−86.6	−86.7	0.1
4-Hydroxybenzoic acid isonicotinamide (VAKTOR)	−192.7	−190.1	−2.6	−149.1	−147.8	−1.3
Bis(urea) oxalic acid (UROXAL01)	−376.4	−357.9	−18.5	−313.8	−307.2	−6.6

#### Elements

Solid elements have been a long-standing challenge for semiempirical methods. This has been due, in part, to several properties that are unique to the pure elements. With very few exceptions, such as manganese and boron, all atoms in a solid element are in the same environment, and therefore ionic terms do not contribute to the bonding. By implication, all bonding must necessarily be due to purely covalent interactions between pairs of atoms of the same type. Also, in contrast to mainstream chemistry, most elements exist as metallic solids, implying the existence of a Fermi surface, which in turn makes solving the SCF equations more difficult. Finally, restricted Hartree–Fock methods are normally unsuitable for modeling metallic behavior, and recourse has to be made to using unrestricted Hartree–Fock methods.

When the elements were modeled using PM6, several elements collapsed to form very dense solids, with metallic cobalt being the most extreme (PM6 predicted its density to be 73.5 g/cc). Such nonsensical results were accompanied by an equally absurd predicted Δ*H*_f_; several elements had values that were more negative than −1,000 kcal/mol^-1^.

All these severe faults in PM6 were addressed during the development of PM7, resulting in some improvement, so that the largest error in the Δ*H*_f_ value predicted for an element using PM7 was +83.1 kcal/mol^-1^, for metallic barium. Most metallic structures were reproduced, the most important exceptions being the elements Fe, Ag, Sn, Ta, and Ba.

#### Charges on ions

As expected, almost all compounds containing metal atoms from groups IA and IIA are more or less ionic, with the charge on the metal atom being large and positive. That the charge is less than the formal oxidation state can be regarded as a measure of the covalent character of the bonds between the metal atom and its neighbors. Of greater interest are the partial charges on polyatomic anions and cations such as sulfate and tetramethylammonium. For most of these species, the uncharged species is either not known, or, as in the case of low-temperature ammonium amalgam, only stable under exotic conditions. However, like the elements of groups IA and IIA, these ions are stable in solids and thus are amenable for study. An estimate of the electropositive nature of these ions can be made by comparing the partial charges on the ions with those of the alkali and alkaline earth metal ions; for the ammonium ions, these range from +0.915 to +0.999, making them extremely electropositive ions (see Table 11).

**Table 11:** Partial charges on the cations in various solids

Solid	Charge
Sodium chloride	0.807
Ammonium benzoate	0.915
Cesium fluoride	0.934
Ammonium sulfate	0.943
Bis(pyridinium) oxalate oxalic acid	0.952
Ammonium iodide	0.965
*Trans*-difluoro-bis(ethylenediamine)-chromium(III) chloride	0.971
Tetramethylammonium benzoate monohydrate (ISIHIB)	0.980
Tetramethylammonium nickel(ii) trichloride	0.982
Bis(tetramethylammonium) hexachloro-platinum(IV)	0.984
Ammonium fluoride	0.988
Bis(pyridinium) hexachloro-rhenium(IV)	0.990
Tetramethylammonium diaqua-tetrafluoro-manganese(III)	0.996
Ammonium hexafluorosilicate	0.999
Copper(II) hexaquo dinitrate	1.713
Tris(ethylenediamine)-cobalt(III) trichloride trihydrate	2.881

A similar situation occurs with electronegative species—typically, polyatomic anions are more ionic than their monatomic counterparts (Table 12). A comparison of the entries in these two tables reveals that the partial charge on even an extremely electronegative ion such as F^−^ is determined mainly by the nature of its counterion, so ammonium is more electropositive than cesium.

**Table 12:** Partial charges on the anions in various solids

Solid	Charge
Nitric acid hydrate	−0.755
(Diphenylmethanide)-(18-crown-6)-rubidium	−0.930
(Cyclopentadienyl)-(18-crown-6)-rubidium dimethoxyethane solvate	−0.935
Ammonium perchlorate	−0.940
18-Crown-6 oxonium hexachloro-tantalum(V)	−1.000
18-Crown-6 oxonium clathrate aqua-pentachloro-titanium(IV)	−1.060
Bis(pyridinium) oxalate oxalic acid	−1.640
Ammonium sulfate	−1.886
Ammonium chromate	−1.978
Bis(pyridinium) hexachloro rhenium(IV)	−1.980
Ammonium hexafluorosilicate	−1.998

#### Minerals

Minerals can be regarded as naturally occurring chemicals that share an important characteristic—with few exceptions, minerals represent the most stable assembly of elements comprising that mineral. A good example is provided by the evaporite trona, Na_3_(CO_3_)(HCO_3_)(H_2_O)_2_. This mineral is formed naturally during the evaporation of saline water, and represents the lowest-energy structure for the particular mix of ions present in the liquor. Very few minerals are not in the lowest-energy state, the most famous example being diamond, but such minerals are rare and the energy of such systems is usually only slightly above the absolute minimum. Because their structures represent low-energy configurations, minerals provide a good test of a modeling method, in that if the method predicts a significantly different structure, the method is definitely in error. In contrast, if a synthesized (i.e., non-naturally occurring) solid is modeled, and the calculated structure is different from that observed, the possibility cannot be dismissed that it might represent a valid polymorph, possibly one that has not yet been characterized.

Several hundred minerals were surveyed, with the majority of the reference structures being obtained from the American Mineralogist Crystal Structure Database [43, 44]. Most structures could be used directly; among those that needed to be modified before they could be used, the most common change was the addition of hydrogen atoms (these atomswere present in the formula, but their positions were not located in the X-ray structure). The addition of hydrogen atoms was a straightforward procedure. Likely positions of putative hydrogen atoms were identified, hydrogen atoms were added as needed, and the positions of the hydrogen atoms were then optimized while holding the rest of the crystal fixed at the X-ray structure. The resulting structure was then used as the reference geometry. In every mineral that required hydrogen atoms to be added, there was no ambiguity in the likely positions of the hydrogen atoms, and, after their positions had been optimized, the modified crystal looked chemically sensible.

The ability of PM7 to model minerals of different mechanical hardness was estimated by surveying Moh’s scale. Moh’s scale provides a good test in that the range of interatomic interactions is very wide, from purely covalent (diamond) through mixed covalent and ionic (apatite) to extremely weak (between layers in talc). Of the ten entries, eight were predicted with good accuracy, calcite and fluoride being the exceptions.

Several hundred different chemical environments were represented in the minerals surveyed, and most were correctly modeled by PM7. In general, PM7 was more successful in modeling mechanically hard minerals than the softer minerals. This could be attributed to a feature of the parameter optimization process: most reference data represent systems with strong covalent bonds as opposed to weak noncovalent bonds; therefore, it is not surprising that hard minerals (i.e., minerals where covalent bonds dominate) are modeled with increased accuracy. On the other hand, when the integrity of the mineral depends on weak noncovalent bonds, small errors in energies could result in large errors in geometries. Examples of this type include gibbsite (Al(OH)_3_), where the layers are held together by hydrogen bonds, and talc (Mg_3_Si_4_O_10_(OH)_2_), where the layers are held together by dispersive and weak electrostatic interactions.

### Proteins

An objective when developing PM7 was to improve the accuracy of prediction of barrier heights in enzyme reactions, so evaluating the ability of PM7 to accurately model proteins is of paramount importance. As proteins are macromolecules, their structures are complicated, and, in the case of enzymes, their reactions involve the interplay of several competing, subtle, phenomena, so that the energy difference between reactant and product is often very small. All of these features make the realistic modeling of proteins and their properties extraordinarily difficult.

Normally, in order for semiempirical methods to work, the chemical system being modeled must be as realistic as possible. Even one extra or one missing atom in a system of several thousand atoms can invalidate any results obtained from the resulting model, so extreme care must be exercised during data preparation if the results are intended to be used to gain insight into biochemical processes. Fortunately, this is not the case here, where the sole objective is to determine how accurately PM7 can reproduce known protein structures. The assumption is that if existing structures can be modeled with good accuracy, then perturbed structures of the type found in biochemical processes would also be modeled with good accuracy, and vice versa.

PM7 was used to model the structures of about 70 proteins in the Protein Data Bank (PDB). Because PDB files normally lack hydrogen atoms and have structural and positional disorder, some preconditioning was necessary before they could be used in modeling. For each protein, only the minimum change necessary was made in order to generate a realistic starting structure; that is, all disorder was resolved, hydrogen atoms were added so as to satisfy valence requirements, and their positions were optimized in a preliminary calculation. The resulting preconditioned geometry will be referred to as the “PDB structure.”

Changes in Δ*H*_f_ and RMS geometry for six representative proteins from this set are presented in Table 13.

**Table 13:** Changes in Δ*H*_f_ and RMS distortion upon going from PDB to PM7 structures for proteins

Protein	Change in Δ*H*_f_ (kcal/mol)				RMS distortion (Å)			
	PDB	Opt-10	Opt-3	Opt	PDB	Opt-10	Opt-3	Opt
Chymotrypsin 1AFQ	2105.7	1083.8	898.2	0.0	0.000	0.055	0.109	1.264
Crambin 1CBN	605.4	402.7	343.6	0.0	0.000	0.115	0.172	0.985
Crambin (solvated) 1CBN	280.4	105.7	83.3	0.0	0.000	0.109	0.139	0.728
Green fluorescent protein 1EMA	2709.5	1121.8	969.4	0.0	0.000	0.067	0.126	0.966
Importin 1QGK	8944.2	4396.5	3011.1	0.0	0.000	0.060	0.116	1.300
Potassium channel 1JVM	3279.4	1507.4	1147.3	0.0	0.000	0.060	0.131	1.130

Using the starting structures, a complete PM7 unconstrained optimization was performed, resulting in an optimized structure (called “Opt”). If the starting PDB geometry was completely accurate and if PM7 could accurately model the chemical system, the PDB and Opt geometries would be identical. This implies that the difference between the PDB and Opt structures is a measure of error. Unfortunately, a comparison of the two structures cannot differentiate between errors in the PDB structures arising from experimental limitations and errors caused by faults in the PM7 theoretical method. In an attempt to assign the errors to experiment or theory, two constrained optimizations were run. An energy penalty function of 10 kcal/mol^-1^/Å^2^ was applied to each atom in the system. This had the effect of adding an energy penalty to the system as the atoms moved away from their starting position, thus effectively biasing the optimized geometry (called “Opt-10”) in favor of the starting PDB structure. Another optimization was carried out where the penalty function was 3 kcal/mol^-1^/Å^2^; here, the resulting geometry (called “Opt-3”) was biased towards the PDB structure, but to a lesser extent.

When a penalty of 10 kcal/mol^-1^/Å^2^ is used, errors in PM7 arising from inaccurate modeling of long-range effects of the type that shape tertiary structure are effectively eliminated, and only errors due to nearest and next-nearest neighbor distances are important. Having already shown that PM7 errors in bond lengths are on the order of 0.02 Å (see Table 3), a reasonable conclusion would be that PM7 would have errors of a similar magnitude when applied to proteins. The RMS distortion in the Opt-10 systems is on the order of 0.05–0.10 Å, a value significantly larger than that expected for PM7, which suggests that the positions of the atoms in the starting PDB structure are in error by about that amount.

Upon shifting to a penalty of 3 kcal/mol^-1^/Å^2^, the RMS error increased, as expected. When the penalty function was removed entirely, the RMS distortion increased dramatically, rising to about 1 Å. This error is considerably larger than any reasonable error in the PDB geometry, so it must be caused by either faults in PM7 or by the model used to represent the PDB geometry. Protein crystals normally contain large amounts of water, particularly in the interprotein interstices, but by convention the positions of these water molecules are not reported. Structures in the PDB should therefore be regarded as representing the hydrated species. Comparing the results of the optimization of gas-phase and condensed-phase (solvated) crambin with the PDB structure shows that the change in Δ*H*_f_ upon going from the PDB to the fully optimized structure drops from 605 to 280 kcal/mol^-1^ (i.e., by more than half). This confirms that the PDB structure is better represented by the solvated form than by the gas-phase form (i.e., that the solvated form is more realistic).

### Barrier heights

Enzymes catalyze many types of reaction, ranging from simple bond-making–bond-breaking reactions that occur only on the singlet or doublet electronic surface to subtle ion pumps and reactions involving excited electronic states. In this work, the range of reactions surveyed was restricted to only the simplest type. Several high-level theory benchmark databases of barrier heights for simple reactions [31–36] were used in the construction of reference data for 97 transition states. Because barrier heights are differences in energies, each reaction was represented by two data, one for the optimized PM7 structure of the precursors and one for the refined PM7 transition state. Reference data could then be expressed as the difference in energy between the transition state and its precursor. Because only a small number of reference data were available, the training set was augmented with reference data on related well-behaved ground-state systems. Parameter optimization proceeded without any complications, taking just a few minutes of CPU time. The resulting method (that is, the theoretical framework of PM7 and the parameters optimized to reproduce activation barriers) was called PM7-TS.

Analysis of the results showed that the average unsigned error in barrier heights calculated using PM7-TS was 3.8 kcal/mol^-1^, as compared with the AUEs for PM7 of 11.0 kcal/mol^-1^ and for PM6 of 12.2 kcal/mol^-1^.

## Discussion

### Use of semiempirical methods to model enzyme reactions

Both PM6 and PM7 were designed to improve the accuracy of the modeling of biochemical macromolecules. PM6 was a significant improvement over the earlier PM3, in that the prediction of geometries and heats of formation was much improved. In turn, PM6 had a severe limitation in that the accuracy of prediction of intermolecular interaction energies—quantities of great importance in biochemistry—was low. Using three proposed modifications to the PM6 method, PM6-DH+, PM6-DH2, and PM6-D3H4, as a guide, a post-SCF correction to address this deficiency was added to PM7; this resulted in a reduction in the error in the intermolecular interaction energies of more than 70 %.

Currently, there are no satisfactory criteria for determining the suitability of a method for modeling proteins. A possible criterion would be the RMS error between the calculated and X-ray structures. This criterion is, however, very sensitive to errors in long-range weak interactions, so even minor changes in energy could result in relatively large changes in the orientation of a protein chain. In addition, minor crystal packing forces could, at least in principle, result in significant changes in the protein chain orientation.

An alternative to using proteins directly would be to examine the applicability of a method used to model organic crystals. Individual solids could be selected that illustrate the types of interaction that occur in proteins. Provided all the types of interaction that occur in proteins were represented in the solids, the ability of a method to model those solids would then be a measure of that method’s ability to model proteins. When PM7 was used to model simple organic solids, AUE in Δ*H*_f_ decreased by more than 60 % and errors in geometries decreased by ∼20 % relative to PM6. If the assumption is made that the ability to model solids translates into the ability to model proteins, then the conclusion follows that PM7 represents a significant improvement.

With the development of PM7-TS, activation barrier heights in simple organic reactions can now be modeled with useful accuracy, a feature not available in related methods such as PM6 and PM7. At the present time, a deficiency exists in that the predictive power of PM7-TS is unknown, but as more and more benchmark transition state barriers become available the accuracy of PM7-TS predictions can be determined, and this deficiency will eventually be corrected. In the event that surveys show that PM7-TS is not useful as a predictive tool, the same reference data could be used to re-parameterize PM7-TS in order to obtain a new method that would be more predictive (parameter re-optimization being extremely simple and requiring only minutes of CPU time).

Assuming that the PM7-TS method does have useful predictive powers, it should now be possible to use purely semiempirical methods to model putative mechanisms in enzyme-catalyzed reactions, including predicting activation barriers as well as heats of formation of reactants and products. Without doubt, this is a formidable task, but when represented as a set of individual steps, and provided care is taken to ensure that each step is performed correctly, the process can be carried out with confidence. Although all of the steps have already been published (either here or in other articles), it might be convenient for users to have a summary of the process involved in modeling an enzyme-catalyzed reaction. That will now be presented.

#### Summary of steps performed to calculate barrier heights

1. An obvious first step is to generate a proposed reaction mechanism. This can be a new putative step or the result of modeling a generally accepted step in a metabolic cycle. Whatever the reason, three stages of the step are needed: a reactant, a transition state, and a product.
2. A starting structure is obtained, with the PDB [45] being an obvious source. An appropriate enzyme with an inhibitor in the active site is particularly useful, as the inhibitor could be used as a guide to the transition state structure.
3. Extensive preconditioning is then done. This would involve adding hydrogen atoms as needed in order to satisfy valence requirements, and then optimizing their positions. As conventional matrix algebra methods are inefficient, this and all subsequent operations should be done using the linear scaling MOZYME technique [46]. During this process, some protons might migrate to nearby functional groups to form salt bridges (i.e., ions might spontaneously form). If the ionization is not correct, then individual protons should be added or deleted as necessary.
4. The entire system would then be allowed to relax. If solvation is considered desirable, the relaxation could be performed in an aqueous environment simulated by Klamt’s COSMO technique [47].
5. At this stage, the inhibitor is now no longer needed, and it should be replaced by the appropriate substrate. The fact that the inhibitor mimicked the transition state can be used as a guide to the geometry of the substrate. Once the substitution is complete, the entire system should be relaxed again. The result corresponds to either the reactant or the product geometry.
6. Based on the result of the previous step, the inhibitor would again be replaced by the substrate, but now the parts of the substrate are moved in such a way that when the system is relaxed again, the resulting geometry corresponds to the other end of the reaction.
7. With both the reactant and product geometries now available, the process of ascending the reaction barrier can be started. This would be carried out in a stepwise manner, using the GEO_REF option [48] in MOPAC2012. At each stage in this process, the geometry (either stressed reactant or stressed product) with the lower energy is optimized, subject to a penalty function based on the difference between the current geometry and that of the higher-energy geometry. Provided the reactant and product geometries were correctly prepared, this process is straightforward, and results in a structure that is a good approximation to the transition state.
8. Transition state refinement is then done. This consists of a repeated two-step process [42]. In the first step, a gradient minimization is carried out. Only those atoms in the immediate vicinity of the reaction site are allowed to move, with all other atoms held frozen. The second step involves energy minimization. In this step, all atoms that were allowed to move in the first step are now frozen, and all atoms that were frozen in the first step are now allowed to move. This two-step process is repeated until the change in results is acceptably small; typically three cycles are needed.
9. Once a stationary point has been located, the vibrational frequencies of the transition state need to be calculated, in order to verify that one and only one imaginary frequency exists. Only the Hessian for the atoms that were involved in the gradient minimization is needed for this step. This has two advantages: first, the computational effort required is reduced—if the gradient minimization used 20 atoms, and the enzyme contained 4,000 atoms, then the computational effort required to construct the Hessian would be reduced to 0.5 %; second, by only generating normal modes for vibrations in the active site, all spurious imaginary frequencies that arise from structures (such as rotating methyl groups) are avoided.
10. At this point, all three geometries are now available: the reactant, the transition state for the putative reaction, and the product. An improved estimate of the barrier height could then be obtained by calculating Δ*H*_f_ for each of the three geometries using PM7-TS.
11. To complete the analysis, the intrinsic reaction coordinate should be calculated. One half would be started using the transition state displaced slightly along the vector of the transition state coordinate; the other half would be started using the transition state displaced slightly along the vector of the transition state coordinate but with the opposite sign.

### Why are semiempirical methods so accurate?

By their nature, semiempirical methods are much simpler than ab initio methods, with most of the time-consuming mathematical operations of ab initio methods being replaced by relatively simple approximations. A result of this simplification is that when chemical systems are modeled using semiempirical instead of ab initio methods, considerably less CPU time is needed. It might be assumed that another consequence of the use of approximations is that the accuracy of prediction would suffer, as semiempirical methods are less complete than their more sophisticated theoretical analogs. Given the results presented here, particularly the fact that the AUEs in predicted heats of formation for well-behaved organic compounds are significantly smaller when PM7 is used than when the much more expensive B3LYP method is used, this assumption is clearly invalid. Even the older PM6 method was of sufficient accuracy to allow many faults in the NIST WebBook to be detected [49]; faults which, once identified, were quickly corrected. The obvious question, then, is: why are modern semiempirical methods so accurate?

Semiempirical methods combine a theoretical framework with empirically determined reference data, or with high-accuracy theoretically generated data. Reference data derived from experimental results are—by definition—accurate, and include all possible theoretical considerations, such as zero-point energy, internal energy, one- and two-electron phenomena, instantaneous correlation, relativistic effects, etc., and any other effects that are as yet unknown. Accurately computing theoretical effects ab initio is obviously very difficult, but by looking to nature as a source of reference data, all the hard work can be avoided: the value of a reference datum obtained from an experimental observation obviously encapsulates all of the phenomena represented by that datum.

By optimizing parameters in semiempirical methods to reproduce reference data, all the complexity of ab initio theory is avoided. If this is done correctly, then because semiempirical methods are optimized to reproduce nature (i.e., the known properties of chemical systems), semiempirical methods should intrinsically be highly accurate. In other words, semiempirical methods are designed to reproduce what is already known, and that they can do so with good accuracy should not be regarded as surprising. A reasonable hope is that such methods should also be predictive.

In contrast, if the objective is to predict chemical properties de novo or ab initio, all possible phenomena involved must be taken into account. For moieties involving only light elements, this implies (at a minimum) CCSD(T)/CBS with corrections made for internal and zero point energies, and relativistic effects and other phenomena also need to be taken into account if heavier elements are involved. This is, in principle, very different from semiempirical methods: ab initio methods, by definition, do not use reference data, so every component of every phenomenon must be accurately calculated if such methods are to be predictive. An error at any stage in an ab initio calculation could invalidate a result. This can be illustrated by a calculation of two isomers of C_14_H_28_ using B3LYP with the default 6-31 G(d) basis set and with internal and zero-point energy corrections. The results predict that the difference in the heats of formation of the isomers *n*-tetradecane and octamethylhexane is 45.5 kcal/mol^-1^, whereas the experimental value is known to be 20.0 kcal/mol^-1^: a discrepancy of 25.5 kcal/mol^-1^. Grimme has reported convincing evidence [50], obtained using isomers of C_8_H_18_, that errors in the energy order of alkane isomers (branching should be lower in energy than linear) predicted using DFT methods can be attributed to the neglect of electron correlation; when a dispersion correction is added, the correct order is obtained [51]. This type of error should not occur in modern semiempirical methods, and indeed, for this particular isomerization, PM7 gives 21.3 kcal/mol^-1^.

### Speculation regarding future improvements

Semiempirical methods have improved steadily over the past few decades, with three main improvements dominating: the methods have become more accurate; the range of application, in terms of both the number of elements and the types of phenomena that can be modeled, has increased; and the predictive power has also increased.

In principle, if three types of error could be eliminated, semiempirical methods would be completely accurate. First, the theoretical framework has to be sufficiently realistic and flexible that the resulting semiempirical model is a good reflection of reality; second, the minimum in parameter space must be located with sufficient accuracy that no modification of the values of the parameters could result in a significant decrease in the error function; and third, the set of reference data must be large enough and versatile enough to allow the minimum in parameter space to be defined (i.e., to ensure that all eigenvalues of the associated parameter Hessian are significantly nonzero).

An estimate of the degree to which each of these objectives has been achieved can be obtained by examining the distribution of errors in heats of formation and in geometries. As large amounts of reference data are available for both of these properties, they are particularly suitable for the following analysis.

#### Errors in the theoretical framework

As mentioned above, the average unsigned error in heats of formation obtained when semiempirical methods are applied to well-behaved compounds has decreased steadily, so that, for PM7, the AUE is 4.0 kcal/mol^-1^. For some other compounds that involve uncommon bonding, errors are sometimes quite large. Thus, in diphenyl disulfone, in which the structure –SO_2_–SO_2_– occurs, the PM7 error is +35.6 kcal/mol^-1^; in cubane, where the C–C–C angle is 90°, the error is −23.1 kcal/mol^-1^; and even in a compound as simple as molecular nitrogen, the PM7 error is +33.0 kcal/mol^-1^. There is no obvious reason to question the accuracy of the reference data for the first two systems, and as the Δ*H*_f_ of molecular nitrogen is, by definition, zero, the large values of these errors, combined with the unusual bonding, suggest that there are faults in the theoretical framework.

Errors in theory can only be corrected by making changes to the algebraic form of the approximations. Designing such changes is difficult, but converting the changes into software is straightforward. In this work, very little effort has been expended to correct faults of this type, because the number of large errors in predicted heats of formation that are attributable to errors in theory is very small, and the types of compound that are badly predicted are well known.

Some quantities need to be modeled with increased accuracy. An error that might be tolerable in a Δ*H*_f_ value might be completely unacceptable if it occurred in an intermolecular interaction. Errors in intermolecular interaction energies averaged about 3.0 kcal/mol^-1^ in PM6, severely limiting its suitability for modeling such phenomena. Several attempts were made to rectify this fault by applying post-SCF corrections, with each attempt involving the addition of a relatively simple molecular mechanics correction; that is, a simple algebraic function with a small number of parameters. Each of these corrections appeared to be simple and obvious; however, the effort required to implement them was nontrivial, with the bulk of the effort being unrelated to the actual logic of the modification. This pattern—the effort involved in investigating the effect of a modification being dominated by considerations of quantities other than the modification itself—has existed from the dawn of semiempirical methods. Fortunately, with the development of computational tools and utilities to assist in manipulating data sets and results, and with the increasing availability of reference data, the ease with which ideas can be tested is increasing, so that, in the near future, it will be possible to quickly test the value of suggestions for changes to the theoretical framework.

#### Incomplete parameter optimization

During the development of the earlier NDDO methods [1, 2], the large computational effort required for parameter optimization prevented the minimum in parameter space from being reached. Despite this limitation, as each new method was developed, the average error dropped significantly. With modern hardware, and advances in the techniques of function minimization, parameter optimization can now be performed much more easily and with greater reliability; indeed, the original MNDO optimization, which took several CPU years of effort when it was first performed, can now be run in less than a minute.

Other features of parameter optimization have also improved. When an element was first parameterized, there were problems with selecting the initial values for the parameters to be optimized, and in at least one case (sulfur) the resulting minimum [52] in parameter space was not the global minimum [53]. With the development of optimized values of parameters for all the chemical elements, this problem can be considered solved. Now, when a modification of an existing method is made, there are obvious choices for the starting values of parameters. Of course, there is still the possibility that for one or more elements the current parameters represent a local and not the global minimum, but as the range of chemistry to be modeled increases, the probability of a false minimum staying undetected steadily decreases. If evidence was discovered that indicated that a given minimum was not the global minimum, that same evidence could then be used as a guide to the true (i.e., the global) minimum.

#### Limitations and errors in reference data

A very large amount of reference data is available for molecular geometries. Among the most important collections are the CSD [28] (over 570,000 entries) and the ICSD [29] (over 150,000 entries); together, these databases cover a very wide range of types of chemical interactions. For thermochemistry, readily available collections of heats of formation for both gas and condensed phases include the JANAF [54], Lange’s handbook [55], CRC [56], and the NIST online WebBook [57]. Several reference data collections [16, 17, 37, 58] of high-quality calculated intermolecular interaction energies have been created in recent years, as have important and even more exotic quantities such as the heights of reaction barriers [32, 35]—again, as the result of high-quality calculations.

All this suggests that there is sufficient reference data to allow semiempirical methods to be parameterized and to allow the accuracy of the resulting method to be determined. In practice, this is unfortunately not the case: there are severe limitations in both the types and quantity of reference data, limitations that are most easily understood by reference to the types of data that are needed for the development of a semiempirical method.

Parameters in a method such as PM7 can be divided into two groups: monatomic and diatomic. For each element, the minimum in parameter space for monatomic parameters can be readily defined by using conventional reference data for compounds and ions involving that element. This was the situation when MNDO was developed. Using only a few tens of compounds, the values of monatomic parameters for H, C, N, and O were optimized, and the resulting minimum in parameter space was well defined.

For diatomic parameters, the situation is completely different. As was demonstrated in PM6, the use of diatomic parameters resulted in a dramatic increase in the accuracy of prediction of the heats of formation and geometries used in the training and survey sets. This increase in accuracy was offset by an equally dramatic decrease in predictive power. In order for the minimum in parameter space for diatomic parameters for even one atom pair to be defined, there had to be a minimum of two reference data that involve the relevant atoms in a bonding or near-bonding environment. This condition is readily satisfied in simple organic chemistry where there are large amounts of data involving every possible diatomic combination. Much of these data involve gas-phase species, and are ideal for use as reference data.

Several problems arise when the range of chemistry is increased. Frequently there is a lack of data, and, even when the data exist, they are often in a form that is not suitable for reference data; that is, they cannot be converted into a discrete gas-phase chemical species. Thus, although the Δ*H*_f_ of the mineral magnesite, MgCO_3_, is known (−265.7 kcal/mol^-1^ [56]), this datum is unsuitable for use as a reference datum because it is not possible to relate the observed Δ*H*_f_ to an isolated gas-phase system.

Given the large number of possible atom pairs (there are 3403 atom pairs for the 82 stable elements), it is not surprising that most are not represented in any current database. Most combinations, such as Sc–Ag or Ti–Ne, are of little importance, but whole sets of combinations, for instance F–X and O–X (X: most elements), are of interest. For the purposes of developing and validating semiempirical methods, it is useful to change the perspective from considering what data are available to considering what reference data are needed, and then examining methods of generating such reference data.

One obvious source would be to use modern highly accurate theoretical methods to generate the needed reference data. Thus far, these methods have been used for only limited applications, such as modeling known species (e.g., known small gas phase systems) or species of specific interest (e.g., gas-phase bimolecular complexes for determining intermolecular energies). For the purposes of method development, other species—ones that have no equivalent in nature—would be needed. Examples of such systems would be Cr^III^Al(OH)_6_ (a model for the Cr–Al interaction in ruby) and [Al(H_2_O)_6_]^3+^ (a model for one of the ions in the alums).

Such a collection would be completely novel and of great use for developing new semiempirical methods. PM7 is now the most accurate of the NDDO-type semiempirical methods, but even within PM7, the accuracy of prediction of thermochemical and geometric properties varies widely from element to element. For “popular” elements, such as those involved in simple organic compounds, the accuracy is relatively high. For “unpopular” elements, that is, elements whose properties are not often measured, either because the element is rare (such as Sc) or because they are simply not easy to work with (such as Ag), the accuracy is low. Consider silver: because it forms few gas-phase compounds, there are few thermochemical reference data; as a result, the various types of diatomic parameter minima are not well defined. A consequence is that the accuracy of PM7 when predicting the properties of silver-containing solids is very low. This result—that the accuracy of the prediction of the properties of compounds of popular elements is high, and that of unpopular elements is low—can be generalized to the strong statement that the accuracy of semiempirical methods is mainly determined by the quality of the reference data. A corollary to this would be that a large increase in accuracy and predictive power could be achieved if there were a source of appropriate reference data.

#### An alternative: avoiding the use of diatomic parameters

That the use of diatomic parameters has resulted in a dramatic increase in accuracy is incontrovertible; equally incontrovertible are the facts that it has resulted in an enormous increase in the number of parameters, and that there is increased difficulty in obtaining suitable reference data. If a recipe or formula could be developed that would reproduce the values of the diatomic parameters, the current requirement that each core–core parameter must be independently optimized could be avoided. Not only would this remove the need for reference data for each diatomic pair—a desirable objective in its own right—but the range of chemistry potentially accessible would be dramatically increased: every possible diatomic interaction would instantly become accessible for modeling.

**Fig. 8:**
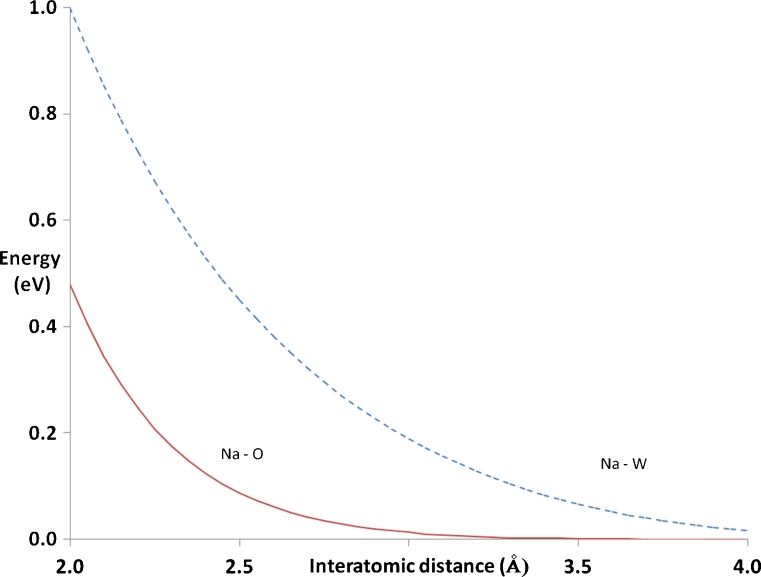
Comparison of potentials arising from W—Na and O—Na unpolarizable core-core terms. Note: nearest interatomic distances in Na_2_WO_4_: Na–O: 2.38 Å, Na–W: 3.79 Å, W–O: 1.82 Å

Examining the values of the diatomic parameters reveals interesting trends. For atoms with small atomic numbers, the diatomic parameters give rise to functions that fall off rapidly as the distance increases, as shown in Fig. 8 for the Na–O interaction, where the function has a value of about 0.1 eV at a Na–O separation of 2.5 Å. For atoms with large atomic numbers, the functions drop off less rapidly, so that for the W–O interaction, the function does not drop below 0.1 eV until the distance increases to 3.3 Å. This behavior suggests that Voityuk’s functions could be identified with atomic size: light atoms are smaller than heavy atoms.

At the present time, there has been no indication of any attempt to develop a formula relating atom pairs to the Voityuk parameters. This inactivity could be attributed to the vagueness or poor definition of the requirements for such an expression. However, with the development of PM6, and now with PM7, there is a guide to the values of such a formula, specifically the collection of values for the various diatomic parameters in these methods. The problem of developing an appropriate equation to reproduce the various core–core interactions would thus devolve to one of fitting two sets of diatomic constants. Presumably such a function would have monatomic parameters and would also depend on atomic number and any number of other quantities. However, regardless of how complicated the function becomes, it is unlikely to have even a significant fraction of the number of terms in the present diatomic interactions.

#### Determining accuracies of methods

As each new semiempirical method is completed, an attempt is made to determine its accuracy relative to similar methods. Of course, if the set of species used to determine accuracy is small, and the new method was optimized to maximize accuracy for that set, then it can always be made to appear to be more accurate than the other methods. If an unbiased measure of accuracy were to be developed then spuriously high accuracies resulting from the use of selected subsets could be avoided.

Somewhat surprisingly, although chemistry is a large field of science, the number of separate data on chemical properties in the various collections is still quite small. Admittedly, for crystals, the present collections of structures are large—in the hundreds of thousands—and are of good quality, but until now, no attempt had been made to determine the accuracy of a theoretical method when it is used to predict the geometries of a large number of solids. For one of the most important properties, Δ*H*_f_ of gas-phase species, there are only a few tens of thousands of experimental data, and for relative energies, such as bimolecular intermolecular interaction energies, experimental data are very limited. In response, the resulting void is now being filled with the results of accurate high-level theoretical calculations.

Because the number of available thermochemical data is quite small, one approach that can be used to prevent the appearance of bias when presenting statistics on the accuracy of semiempirical methods would be to use all available reference data on thermochemistry. With modern hardware, running a survey of even a few tens of thousands of species is not a daunting task, and the resulting statistics would be more credible than those generated using smaller survey sets, where the possibility of biasing the survey set in favor of the *method de jour* could not be discounted.

During the testing of PM7, an attempt was made to include as many thermochemical data as was practical, for both gas-phase species and for solids. This attempt was limited in that only a fraction of the available data from databases was used, and although strong evidence was found that some of the data were inaccurate, no method that was proven to be free from bias was developed to allow faulty data to be excluded. Despite these limitations, the statistical results presented here are believed (at least, by the author) to be more reliable than those of any earlier semiempirical method.

Many of these problems could be eliminated if a single database of thermochemical data of unchallenged accuracy were available as a source of reference data for training sets and for validating the resulting methods. Ideally, such a database would contain experimental results augmented by very high level theoretical results, particularly for properties (such as intermolecular interaction energies) that would be difficult to obtain by any experimental method.

## Conclusions

The use of semiempirical methods as a practical tool for modeling chemical systems has been extended to a wider range of species. A significant increase in accuracy was achieved after relatively minor changes were made to the approximations and after proxy reference data functions representing noncovalent interactions were introduced. The result was that the AUE in the heats of formation of organic solids calculated using PM7 decreased by more than 50 % relative to that for PM6, previously the most accurate of the NDDO methods. At the same time, errors in PM7 geometries have been reduced by over one-third relative to those of PM6. Barrier heights for simple reactions of the type catalyzed by enzymes were reproduced with an AUE that was less than one-third that of PM6.

An examination of the causes of the remaining sources of error suggests that further increases in accuracy could be achieved mainly by improving the training and survey reference data sets. Currently, there is a severe shortage of reference data, resulting in large sections of parameter space that are undefined, or, in chemical terms, causing any resulting method to be severely limited in its range of applicability. There is strong evidence that many existing reference data are also of questionable accuracy, so that a significant fraction of the error in methods can be attributed to sources other than the method itself.

Incomplete parameter optimization, one of the other two possible sources of error in semiempirical methods, can now be eliminated as a significant source of error; this operation has become as reliable and uncomplicated as optimizing a molecular structure in conventional computational chemistry modeling.

The only other source of error lies in the theoretical framework or set of approximations used. With minor exceptions, the current set of approximations has proven to be remarkably robust, allowing a wide range of types of system to be modeled. Future changes in approximations are likely to focus on correcting errors in existing approximations, and not on radically new features. Thus, in recent years, several different approaches to modeling intermolecular interactions have been proposed. The strengths and weaknesses of these ideas have been explored and examined, and this field can be considered to be evolving rapidly in a very healthy way. The specific approximation for intermolecular interactions used in PM7 should be regarded as merely one of a number of competing models, and will almost certainly be replaced by a better model in the future.

## Electronic supplementary material

Below is the link to the electronic supplementary material.

ESM 1 (DOC 452 kb)
