# Supplementary material for: Optimization of parameters for semiempirical methods VI: more modifications to the NDDO approximations and re-optimization of parameters
Source: J Mol Model. 2012 Nov 28;19(1):1–32. doi: 10.1007/s00894-012-1667-x (PMC3536963; doi:10.1007/s00894-012-1667-x)
Supplement: Supplementary file 1 — (DOC 452 kb) [file 894_2012_1667_MOESM1_ESM.doc]

# Supplementary Material

Article title: Optimization of Parameters for Semiempirical Methods VI: More Modifications to the NDDO Approximations and Re-optimization of Parameters

Journal: Journal of Molecular Modeling

Author: James J. P. Stewart
Affiliation: Stewart Computational Chemistry
E-mail: [MrMOPAC@OpenMOPAC.net](mailto:MrMOPAC@OpenMOPAC.net)

# Raw data and Statistical Analysis

All the raw data and statistical analyses presented in this article can be accessed from the web-page <http://openmopac.net/PM7_accuracy/PM7_accuracy.html>

# Definition of the PM7 method

PM7 is similar to the PM6 method (Stewart JJP “Optimization of Parameters for Semiempirical Methods V: Modification of NDDO Approximations and Application to 70 Elements” (2007), J. Mol. Modeling 13:1173:1213). The differences between PM6 and PM7 are the following:

## Two-electron, two-center integrals

### <ss|ss>, <ss|pp>, <pp|p’p’>, <ss|dd>, etc

All two-electron two-center integrals of the type$<aa|bb>$, involving atoms A and B, are modified so that, as the distance between the centers increases, the integral $<aa|bb>$converges on the exact point-charge 1/R_A,B_ value. The modification is to replace the default equation, $<aa|bb>\approx\left(R^{2}+\frac{1}{4}\left(\frac{1}{G_{A}}+\frac{1}{G_{B}}\right)^{2}\right)^{-1/2}$, with$<aa|bb>\approx\frac{1}{R}e^{-0.22(R-7)^{2}}+(1-e^{-0.22(R-7)^{2}})\left(R^{2}+\frac{1}{4}\left(\frac{1}{G_{A}}+\frac{1}{G_{B}}\right)^{2}\right)^{-1/2}$for distances of less than 7 Ångstroms, and with the point-charge at all greater distances.

In these equations, the “≈” symbol refers to the original PM6 NDDO expression only.

For all these sets, the average of the integrals in each set is set equal to the <ss|ss> integral. This only has significance in the region 2 – 7 Ångstroms.

### <ab|cd>

All other integrals are modified so that, as the distance between the centers increases, the integral $<ab|cd>$converges to zero. The modification is to multiply the default equation for the integral by$(1-e^{-0.22(R-7)^{2}})$in the domain < 7 Ångstroms, and to set the integral to zero in the domain > 7 Ångstroms.

## Core-electron attraction integrals, V_μν,B_

Integrals of this type, see Dewar MJS, Thiel W (1977) J. Am. Chem. Soc. 99:4899-4907 for definition, where μ = ν are modified so that the average of the integrals in each set is equal to the <ss|ss> integral. (This modification is necessary in order to ensure no net attraction or repulsion between well-separated neutral atoms, and affects a maximum of 5 integrals per diatomic pair)

## Hydrogen bond s

A modification of Korth’s PM6-DH+ method (Korth M (2010) “Third-Generation Hydrogen-Bonding Corrections for Semiempirical QM Methods and Force Fields” J. Chem. Theory Comp. 6:3808-3816) for modeling hydrogen bonds was used. The changes made were:

*C_element_* for N (-0.16) was replaced with -0.171271, and for O (-0.12) was replaced with -0.098822.

The energy of stabilization in Korth’s Equation 3, *E_H-bond_*, due to hydrogen bonding is given by:

$$E_{H-bond}=\frac{C_{AB}}{R_{AB}^{2}}\cos(\theta_{A})^{2}(1-(1-\cos(\theta_{A})\cos(\varphi_{A})\cos(\psi_{A})\cos(\varphi_{B})\cos(\psi_{B}))^{2})f_{bond}f_{damp}$$

This function avoids the cusp behavior noted by Řezáč and Hobza (Řezáč J, Hobza P (2012) “Advanced Corrections of Hydrogen Bonding and Dispersion for Semiempirical Quantum Mechanical Methods” J. Chem. Theory and Comp. 8:141-151.

$f_{damp}$ is similar to Korth’s , except that when the *R_A-H_* distance differs from the *R_B-H_* distance by less than 0.5 Ångstroms, $f_{damp}$ is set to unity. This only occurs in very strong hydrogen bonded systems.

$f_{damp}$is identical to Korth’s $f_{damp}$.

Extra stabilization is associated with very short O – O distances, *R_O-O_*, of the type that occur in water ions such as [H_5_O_2_]^+^. To allow for this, the hydrogen bond energy, in Kcal/mol, is increased by:

$$(2.5e^{(-80(\max(R_{O-O}-2.67,0)^{2})}\cos(O-H-O)^{4}$$

In this expression, *O-H-O* is the angle between O, H, and O.

## Dispersion

The dispersion functions described by Jurecka, et.al, (Jurecka P, Cerny J, Hobza P, Salahub DR (2007) “Density Functional Theory Augmented with an Empirical Dispersion Term. Interaction Energies and Geometries of 80 Noncovalent Complexes Compared with Ab Initio Quantum Mechanics Calculations” J Comp Chem 28:555-569) as implemented in PM6 by Korth, et.al, (Korth M, Pitonák M, Rezác J, Hobza P (2010) “A Transferable H-bonding Correction For Semiempirical Quantum-Chemical Methods” J Chem Theory and Computation 6:344-352 were used in PM7, with the *Sr* in Korth’s article of 1.04 replaced by 1.226593, R of 20.0 replaced by 15.450118, and S6 of 0.89 replaced by 2.286419. For solids, the dispersion term was modified as described in the PM7 article.

## Parameters used in PM7

Parameter Type Element Parameter

USS H -11.070112

BETAS H -8.389745

ZS H 1.260237

GSS H 14.149656

FN11 H 0.177854

FN21 H 1.428710

FN31 H 0.991324

ALPB_H H 4.051163

XFAC_H H 2.845627

USS HE -31.770969

UPP HE -5.856382

BETAS HE -58.903774

BETAP HE -37.039974

ZS HE 3.313204

ZP HE 3.657133

GSS HE 9.445299

GSP HE 11.201419

GPP HE 9.214548

GP2 HE 13.046115

HSP HE 0.299954

ALPB_H HE 2.989881

XFAC_H HE 2.371199

ALPB_HE HE 3.783559

XFAC_HE HE 3.450900

USS LI -4.804124

UPP LI -2.450842

BETAS LI -2.082310

BETAP LI -27.085547

ZS LI 0.804974

ZP LI 6.027530

GSS LI 9.175811

GSP LI 16.614419

GPP LI 14.193195

GP2 LI 11.289123

HSP LI 3.533317

ALPB_H LI 1.265105

XFAC_H LI 0.488118

ALPB_HE LI 2.982569

XFAC_HE LI 8.316732

ALPB_LI LI 3.213216

XFAC_LI LI 16.832394

USS BE -17.427477

UPP BE -14.843910

BETAS BE -3.965129

BETAP BE -8.623194

ZS BE 1.036199

ZP BE 1.764629

GSS BE 9.590009

GSP BE 9.878338

GPP BE 8.195145

GP2 BE 10.136549

HSP BE 0.966381

ALPB_H BE 2.854611

XFAC_H BE 3.447327

ALPB_HE BE 3.367214

XFAC_HE BE 12.563185

ALPB_LI BE 2.432991

XFAC_LI BE 11.306301

ALPB_BE BE 2.042783

XFAC_BE BE 1.680607

USS B -26.613990

UPP B -23.468278

BETAS B -7.509528

BETAP B -3.775165

ZS B 1.560481

ZP B 1.449712

GSS B 6.418667

GSP B 10.191243

GPP B 5.675076

GP2 B 6.689156

HSP B 0.942923

ALPB_H B 2.314226

XFAC_H B 1.135897

ALPB_HE B 3.163140

XFAC_HE B 1.974170

ALPB_LI B 3.000118

XFAC_LI B 5.549420

ALPB_BE B 1.991820

XFAC_BE B 1.213171

ALPB_B B 2.181999

XFAC_B B 0.964011

USS C -51.372620

UPP C -40.135421

BETAS C -14.414930

BETAP C -7.893717

ZS C 1.942244

ZP C 1.708723

GSS C 12.347323

GSP C 11.932801

GPP C 10.452226

GP2 C 9.385498

HSP C 0.802634

FN11 C 0.045888

FN21 C 5.037055

FN31 C 1.588715

ALPB_H C 1.038716

XFAC_H C 0.204582

ALPB_HE C 3.042705

XFAC_HE C 3.213971

ALPB_LI C 3.061752

XFAC_LI C 7.351225

ALPB_BE C 2.798358

XFAC_BE C 2.803943

ALPB_B C 2.650092

XFAC_B C 1.456708

ALPB_C C 2.655746

XFAC_C C 0.937818

USS N -61.623260

UPP N -48.949816

BETAS N -22.110958

BETAP N -15.464433

ZS N 2.354344

ZP N 2.028288

GSS N 11.881002

GSP N 9.572117

GPP N 12.210140

GP2 N 10.299947

HSP N 2.977433

FN11 N 0.015143

FN21 N 4.734148

FN31 N 1.516714

ALPB_H N 1.049958

XFAC_H N 0.173966

ALPB_HE N 2.814339

XFAC_HE N 1.077861

ALPB_LI N 2.205062

XFAC_LI N 1.070477

ALPB_BE N 2.391056

XFAC_BE N 1.588438

ALPB_B N 2.264882

XFAC_B N 0.794623

ALPB_C N 2.734636

XFAC_C N 0.959163

ALPB_N N 2.786676

XFAC_N N 0.863430

USS O -96.087736

UPP O -71.087738

BETAS O -67.776339

BETAP O -20.267981

ZS O 5.972309

ZP O 2.349017

GSS O 14.955121

GSP O 16.088521

GPP O 12.403337

GP2 O 10.499706

HSP O 5.028656

FN11 O -0.016243

FN21 O 1.871970

FN31 O 1.844360

ALPB_H O 1.508030

XFAC_H O 0.160602

ALPB_HE O 3.595772

XFAC_HE O 6.688705

ALPB_LI O 2.090902

XFAC_LI O 0.499065

ALPB_BE O 3.081366

XFAC_BE O 2.735812

ALPB_B O 2.694696

XFAC_B O 1.106827

ALPB_C O 2.850931

XFAC_C O 0.826848

ALPB_N O 2.841313

XFAC_N O 0.699190

ALPB_O O 2.457135

XFAC_O O 0.356439

USS F -137.395658

UPP F -98.051239

BETAS F -69.725688

BETAP F -29.746124

ZS F 6.070030

ZP F 2.930631

GSS F 13.745134

GSP F 17.991219

GPP F 9.188912

GP2 F 12.322503

HSP F 2.906187

ALPB_H F 3.104012

XFAC_H F 0.587775

ALPB_HE F 2.856543

XFAC_HE F 0.745107

ALPB_LI F 3.036354

XFAC_LI F 0.756703

ALPB_BE F 3.328175

XFAC_BE F 2.273878

ALPB_B F 2.841215

XFAC_B F 0.892467

ALPB_C F 3.230134

XFAC_C F 0.946026

ALPB_N F 3.207271

XFAC_N F 0.894011

ALPB_O F 3.140885

XFAC_O F 0.698033

ALPB_F F 4.492940

XFAC_F F 3.111004

USS NE -2.978729

UPP NE -85.441118

BETAS NE -69.793475

BETAP NE -33.261962

ZS NE 6.000148

ZP NE 3.834528

GSS NE 19.999574

GSP NE 16.896951

GPP NE 8.963560

GP2 NE 16.027799

HSP NE 1.779280

ALPB_H NE 5.999680

XFAC_H NE 5.535021

ALPB_HE NE 3.677758

XFAC_HE NE 1.960924

ALPB_LI NE 2.242969

XFAC_LI NE 0.642933

ALPB_BE NE 0.832530

XFAC_BE NE 0.140208

ALPB_B NE 2.756190

XFAC_B NE 2.764140

ALPB_C NE 3.441188

XFAC_C NE 5.468780

ALPB_N NE 4.426370

XFAC_N NE 29.999609

ALPB_O NE 2.906840

XFAC_O NE 0.753518

ALPB_F NE 3.675611

XFAC_F NE 2.706754

ALPB_NE NE 5.180440

XFAC_NE NE 0.500000

USS NA -5.815476

UPP NA -3.731003

BETAS NA 8.483380

BETAP NA -5.735680

ZS NA 1.666701

ZP NA 1.397571

GSS NA 20.011368

GSP NA 20.020053

GPP NA 12.820792

GP2 NA 19.015416

HSP NA 5.020547

ALPB_H NA 1.619287

XFAC_H NA 1.966963

ALPB_HE NA 2.171840

XFAC_HE NA 4.590369

ALPB_LI NA 0.898897

XFAC_LI NA 0.655446

ALPB_BE NA 1.255480

XFAC_BE NA 3.121620

ALPB_B NA 2.476698

XFAC_B NA 8.164739

ALPB_C NA 2.394648

XFAC_C NA 6.318544

ALPB_N NA 2.482865

XFAC_N NA 5.755473

ALPB_O NA 2.699675

XFAC_O NA 8.556302

ALPB_F NA 3.036873

XFAC_F NA 9.250233

ALPB_NE NA 1.469568

XFAC_NE NA 0.697745

ALPB_NA NA 1.994455

XFAC_NA NA 9.335783

USS MG -14.858681

UPP MG -12.451227

BETAS MG -12.576970

BETAP MG -0.702739

ZS MG 1.170297

ZP MG 1.840439

GSS MG 7.480635

GSP MG 9.602125

GPP MG 8.869755

GP2 MG 6.241718

HSP MG 0.992746

ALPB_H MG 2.423259

XFAC_H MG 6.170068

ALPB_HE MG 2.289485

XFAC_HE MG 3.779366

ALPB_LI MG 1.374791

XFAC_LI MG 2.510632

ALPB_BE MG 1.593445

XFAC_BE MG 2.960809

ALPB_B MG 2.466919

XFAC_B MG 6.072802

ALPB_C MG 2.321772

XFAC_C MG 3.390341

ALPB_N MG 2.025732

XFAC_N MG 2.115961

ALPB_O MG 2.730174

XFAC_O MG 2.888295

ALPB_F MG 3.378507

XFAC_F MG 5.439497

ALPB_NE MG 0.922342

XFAC_NE MG 0.452636

ALPB_NA MG 1.682212

XFAC_NA MG 8.429332

ALPB_MG MG 2.055257

XFAC_MG MG 20.557591

USS AL -32.518856

UPP AL -24.873064

UDD AL -31.418925

BETAS AL 6.109627

BETAP AL -2.986557

BETAD AL -28.937998

ZS AL 1.232599

ZP AL 1.219336

ZD AL 1.617502

ZSN AL 2.346908

ZPN AL 1.529050

ZDN AL 3.682742

ALP AL 5.341685

GSS AL 10.347944

GSP AL 9.180517

GPP AL 7.181623

GP2 AL 5.626787

HSP AL 0.920418

ALPB_H AL 1.610842

XFAC_H AL 1.183718

ALPB_HE AL 2.255830

XFAC_HE AL 2.701400

ALPB_LI AL 1.248327

XFAC_LI AL 0.929842

ALPB_BE AL 1.916502

XFAC_BE AL 4.229824

ALPB_B AL 1.990198

XFAC_B AL 2.676137

ALPB_C AL 2.058949

XFAC_C AL 3.161882

ALPB_N AL 1.477524

XFAC_N AL 0.883919

ALPB_O AL 2.054038

XFAC_O AL 1.619036

ALPB_F AL 2.253927

XFAC_F AL 1.368035

ALPB_NE AL 2.528574

XFAC_NE AL 1.702157

ALPB_NA AL 1.141388

XFAC_NA AL 1.128163

ALPB_MG AL 1.455074

XFAC_MG AL 1.821180

ALPB_AL AL 1.224852

XFAC_AL AL 1.669052

USS SI -41.586357

UPP SI -36.694055

UDD SI -16.775635

BETAS SI -10.755885

BETAP SI -3.922152

BETAD SI -4.736877

ZS SI 1.433994

ZP SI 1.671776

ZD SI 1.221915

ZSN SI 2.002570

ZPN SI 0.818377

ZDN SI 2.591238

GSS SI 8.159128

GSP SI 11.213512

GPP SI 8.521933

GP2 SI 8.493112

HSP SI 0.959479

ALPB_H SI 1.542308

XFAC_H SI 0.688945

ALPB_HE SI 2.028628

XFAC_HE SI 1.976149

ALPB_LI SI 1.911808

XFAC_LI SI 2.989391

ALPB_BE SI 2.162457

XFAC_BE SI 4.322374

ALPB_B SI 1.915795

XFAC_B SI 1.162577

ALPB_C SI 1.673306

XFAC_C SI 0.501779

ALPB_N SI 1.854197

XFAC_N SI 0.671576

ALPB_O SI 1.824047

XFAC_O SI 0.502254

ALPB_F SI 2.160762

XFAC_F SI 0.564372

ALPB_NE SI 2.655346

XFAC_NE SI 12.754805

ALPB_NA SI 1.842304

XFAC_NA SI 9.125996

ALPB_MG SI 1.157990

XFAC_MG SI 0.527802

ALPB_AL SI 1.300963

XFAC_AL SI 1.290056

ALPB_SI SI 1.109923

XFAC_SI SI 0.369696

USS P -66.928393

UPP P -30.435889

UDD P -9.320605

BETAS P -45.578128

BETAP P -12.487910

BETAD P -34.182622

ZS P 2.257933

ZP P 1.555172

ZD P 1.235995

ZSN P 4.925330

ZPN P 1.040649

ZDN P 12.110811

GSS P 3.279518

GSP P 7.561540

GPP P 5.922736

GP2 P 3.990430

HSP P 1.699233

ALPB_H P 1.694074

XFAC_H P 1.829199

ALPB_HE P 2.076667

XFAC_HE P 1.493985

ALPB_LI P 1.727121

XFAC_LI P 5.864987

ALPB_BE P 1.872176

XFAC_BE P 2.310001

ALPB_B P 1.742693

XFAC_B P 2.541187

ALPB_C P 1.770260

XFAC_C P 1.381969

ALPB_N P 1.958580

XFAC_N P 1.312039

ALPB_O P 2.293080

XFAC_O P 1.314436

ALPB_F P 2.513083

XFAC_F P 1.086188

ALPB_NE P 2.243688

XFAC_NE P 0.762937

ALPB_NA P 1.518961

XFAC_NA P 3.713750

ALPB_MG P 1.297069

XFAC_MG P 1.585367

ALPB_AL P 1.375504

XFAC_AL P 3.249399

ALPB_SI P 0.895674

XFAC_SI P 0.616954

ALPB_P P 1.329209

XFAC_P P 2.558579

USS S -51.157757

UPP S -40.352643

UDD S -48.529935

BETAS S -11.422550

BETAP S -7.191896

BETAD S -10.695329

ZS S 2.046153

ZP S 1.807678

ZD S 3.510309

ZSN S 1.131343

ZPN S 0.823803

ZDN S 2.296065

GSS S 8.728478

GSP S 6.483871

GPP S 7.357401

GP2 S 6.875448

HSP S 3.012199

ALPB_H S 2.182464

XFAC_H S 0.703252

ALPB_HE S 1.959149

XFAC_HE S 0.437618

ALPB_LI S 1.737806

XFAC_LI S 0.566769

ALPB_BE S 2.575836

XFAC_BE S 3.179465

ALPB_B S 2.363313

XFAC_B S 1.177082

ALPB_C S 2.429136

XFAC_C S 0.843145

ALPB_N S 2.653791

XFAC_N S 1.197307

ALPB_O S 2.508022

XFAC_O S 0.729340

ALPB_F S 2.533157

XFAC_F S 0.534080

ALPB_NE S 2.787058

XFAC_NE S 3.296160

ALPB_NA S 2.614090

XFAC_NA S 6.263298

ALPB_MG S 1.442313

XFAC_MG S 0.578881

ALPB_AL S 1.706655

XFAC_AL S 1.677290

ALPB_SI S 1.647931

XFAC_SI S 0.553963

ALPB_P S 1.596824

XFAC_P S 1.189185

ALPB_S S 1.985120

XFAC_S S 0.509363

USS CL -68.847511

UPP CL -56.857991

UDD CL -49.258143

BETAS CL -2.893931

BETAP CL -13.528255

BETAD CL 1.888153

ZS CL 2.223076

ZP CL 2.264466

ZD CL 0.949994

ZSN CL 1.992900

ZPN CL 1.874460

ZDN CL 5.469221

GSS CL 11.540894

GSP CL 9.032229

GPP CL 8.457262

GP2 CL 7.988115

HSP CL 5.000141

ALPB_H CL 2.548456

XFAC_H CL 0.721003

ALPB_HE CL 1.671634

XFAC_HE CL 0.500002

ALPB_LI CL 2.838217

XFAC_LI CL 2.531354

ALPB_BE CL 2.716560

XFAC_BE CL 2.638266

ALPB_B CL 2.228737

XFAC_B CL 0.742613

ALPB_C CL 2.450283

XFAC_C CL 0.690352

ALPB_N CL 2.385624

XFAC_N CL 0.659831

ALPB_O CL 2.288635

XFAC_O CL 0.437776

ALPB_F CL 2.575402

XFAC_F CL 0.503186

ALPB_NE CL 1.732740

XFAC_NE CL 0.499482

ALPB_NA CL 2.536945

XFAC_NA CL 10.364642

ALPB_MG CL 2.292455

XFAC_MG CL 2.207847

ALPB_AL CL 1.678498

XFAC_AL CL 1.079875

ALPB_SI CL 1.818389

XFAC_SI CL 0.590060

ALPB_P CL 1.297513

XFAC_P CL 0.496813

ALPB_S CL 2.167945

XFAC_S CL 0.624384

ALPB_CL CL 2.469990

XFAC_CL CL 0.900377

USS AR -7.797931

UPP AR -83.211487

BETAS AR -8.839842

BETAP AR -28.427303

ZS AR 6.000272

ZP AR 5.949170

GSS AR 17.858776

GSP AR 4.168451

GPP AR 11.852500

GP2 AR 15.669543

HSP AR 4.574549

ALPB_H AR 4.056167

XFAC_H AR 3.933445

ALPB_HE AR 2.716562

XFAC_HE AR 1.177211

ALPB_LI AR 3.001334

XFAC_LI AR 2.193788

ALPB_BE AR 3.227598

XFAC_BE AR 2.700296

ALPB_B AR 2.674207

XFAC_B AR 2.017996

ALPB_C AR 1.471309

XFAC_C AR 0.122309

ALPB_N AR 2.326805

XFAC_N AR 0.562581

ALPB_O AR 2.228209

XFAC_O AR 0.367713

ALPB_F AR 3.920658

XFAC_F AR 9.269715

ALPB_NE AR 2.963747

XFAC_NE AR 1.304697

ALPB_NA AR 2.667734

XFAC_NA AR 5.946915

ALPB_MG AR 1.996514

XFAC_MG AR 2.030224

ALPB_AL AR 2.716128

XFAC_AL AR 1.838228

ALPB_SI AR 1.935869

XFAC_SI AR 1.288907

ALPB_P AR 3.998905

XFAC_P AR 0.173766

ALPB_S AR 2.049398

XFAC_S AR 0.653769

ALPB_CL AR 2.554449

XFAC_CL AR 2.256094

ALPB_AR AR 2.306432

XFAC_AR AR 0.972699

USS K -4.888065

UPP K -3.763457

BETAS K 10.013029

BETAP K -2.882668

ZS K 5.422018

ZP K 1.471023

GSS K 19.497974

GSP K 4.674636

GPP K 4.339481

GP2 K 5.981455

HSP K 1.092988

ALPB_H K 2.304518

XFAC_H K 29.964954

ALPB_HE K 2.140614

XFAC_HE K 6.673621

ALPB_LI K 1.108062

XFAC_LI K 4.364297

ALPB_BE K 3.000365

XFAC_BE K 6.514383

ALPB_B K 2.507524

XFAC_B K 28.190857

ALPB_C K 1.769643

XFAC_C K 2.489951

ALPB_N K 1.907394

XFAC_N K 3.943077

ALPB_O K 2.151119

XFAC_O K 4.281570

ALPB_F K 3.065393

XFAC_F K 17.321092

ALPB_NE K 1.653125

XFAC_NE K 1.093188

ALPB_NA K 0.944935

XFAC_NA K 6.450008

ALPB_MG K 1.272102

XFAC_MG K 2.832505

ALPB_AL K 1.849469

XFAC_AL K 27.774025

ALPB_SI K 1.674691

XFAC_SI K 8.047633

ALPB_P K 1.415563

XFAC_P K 4.258021

ALPB_S K 2.428403

XFAC_S K 30.000181

ALPB_CL K 2.346443

XFAC_CL K 12.630753

ALPB_AR K 2.436124

XFAC_AR K 8.318024

ALPB_K K 1.492751

XFAC_K K 6.173527

USS CA -13.503503

UPP CA -10.559344

BETAS CA -11.696053

BETAP CA 4.968210

ZS CA 1.477988

ZP CA 2.220194

GSS CA 7.914200

GSP CA 6.712903

GPP CA 4.997910

GP2 CA 4.995881

HSP CA 1.170905

ALPB_H CA 1.997037

XFAC_H CA 5.125996

ALPB_HE CA 2.150217

XFAC_HE CA 5.381385

ALPB_B CA 1.700010

XFAC_B CA 1.700010

ALPB_C CA 3.376881

XFAC_C CA 45.518388

ALPB_N CA 2.335548

XFAC_N CA 3.063067

ALPB_O CA 3.347983

XFAC_O CA 8.353090

ALPB_F CA 3.871263

XFAC_F CA 14.692101

ALPB_NE CA 1.247453

XFAC_NE CA 0.493997

ALPB_NA CA 2.172223

XFAC_NA CA 10.049083

ALPB_MG CA 1.612133

XFAC_MG CA 5.062878

ALPB_AL CA 1.612565

XFAC_AL CA 4.188555

ALPB_SI CA 1.730018

XFAC_SI CA 4.282139

ALPB_P CA 1.922605

XFAC_P CA 15.033250

ALPB_S CA 1.481189

XFAC_S CA 0.561550

ALPB_CL CA 2.785624

XFAC_CL CA 8.996518

ALPB_AR CA 1.544903

XFAC_AR CA 0.699868

ALPB_K CA 1.210391

XFAC_K CA 1.755307

ALPB_CA CA 1.477787

XFAC_CA CA 5.134189

USS SC -19.383239

UPP SC -15.936628

UDD SC -20.365590

BETAS SC -16.127750

BETAP SC -4.714646

BETAD SC -8.631714

ZS SC 1.794897

ZP SC 2.174934

ZD SC 5.992860

ZSN SC 1.314009

ZPN SC 1.020629

ZDN SC 1.437857

ALP SC 0.991198

GSS SC 7.183554

GSP SC 6.188166

GPP SC 6.079855

GP2 SC 5.329586

HSP SC 1.340355

POC SC 1.070880

F0SD SC 8.096837

G2SD SC 3.531412

ALPB_H SC 2.630734

XFAC_H SC 5.354101

ALPB_C SC 2.774943

XFAC_C SC 13.452840

ALPB_N SC 2.081124

XFAC_N SC 1.980291

ALPB_O SC 2.238586

XFAC_O SC 1.567669

ALPB_F SC 3.226175

XFAC_F SC 7.919620

ALPB_AL SC 1.003550

XFAC_AL SC 0.500620

ALPB_SI SC 1.849600

XFAC_SI SC 2.767826

ALPB_P SC 1.919608

XFAC_P SC 4.663061

ALPB_S SC 1.111949

XFAC_S SC 0.498540

ALPB_CL SC 2.094163

XFAC_CL SC 2.355302

ALPB_SC SC 2.106571

XFAC_SC SC 30.002441

USS TI -26.608414

UPP TI -23.616842

UDD TI -28.876758

BETAS TI -5.411644

BETAP TI -4.838856

BETAD TI 0.774574

ZS TI 1.448579

ZP TI 1.940695

ZD TI 1.093648

ZSN TI 1.078295

ZPN TI 4.663707

ZDN TI 0.954258

GSS TI 5.894930

GSP TI 7.330203

GPP TI 27.781556

GP2 TI 24.353243

HSP TI 0.044555

F0SD TI 6.384127

G2SD TI 3.488564

ALPB_H TI 1.447725

XFAC_H TI 0.603333

ALPB_LI TI 1.514050

XFAC_LI TI 0.502488

ALPB_B TI 1.628710

XFAC_B TI 0.649360

ALPB_C TI 1.798067

XFAC_C TI 0.562296

ALPB_N TI 1.638936

XFAC_N TI 0.543706

ALPB_O TI 1.962314

XFAC_O TI 0.872204

ALPB_F TI 2.186657

XFAC_F TI 0.836131

ALPB_NA TI 1.124786

XFAC_NA TI 1.987793

ALPB_MG TI 1.900606

XFAC_MG TI 6.889073

ALPB_AL TI 1.833384

XFAC_AL TI 8.952566

ALPB_SI TI 1.373954

XFAC_SI TI 0.561089

ALPB_P TI 1.610003

XFAC_P TI 3.074680

ALPB_S TI 2.309450

XFAC_S TI 1.781817

ALPB_CL TI 1.953656

XFAC_CL TI 0.831301

ALPB_CA TI 1.268314

XFAC_CA TI 0.513504

ALPB_TI TI 2.445684

XFAC_TI TI 29.795082

USS V -32.598954

UPP V -20.496422

UDD V -43.169867

BETAS V -4.628385

BETAP V -3.039568

BETAD V -3.704203

ZS V 6.051795

ZP V 2.249871

ZD V 1.087345

ZSN V 1.215500

ZPN V 0.877260

ZDN V 1.512555

GSS V 6.645015

GSP V 5.436952

GPP V 5.225810

GP2 V 4.580932

HSP V 1.092636

F0SD V 6.560730

G2SD V 1.196816

ALPB_H V 1.454900

XFAC_H V 0.350807

ALPB_C V 1.904429

XFAC_C V 0.489034

ALPB_N V 2.139547

XFAC_N V 0.964593

ALPB_O V 2.076717

XFAC_O V 0.789091

ALPB_F V 2.483525

XFAC_F V 1.056377

ALPB_NA V 2.548904

XFAC_NA V 8.346697

ALPB_P V 2.205190

XFAC_P V 6.763663

ALPB_S V 2.407934

XFAC_S V 1.374332

ALPB_CL V 2.395745

XFAC_CL V 1.590959

ALPB_K V 1.361275

XFAC_K V 1.893631

ALPB_V V 1.859935

XFAC_V V 0.953942

USS CR -41.077064

UPP CR -19.350873

UDD CR -80.190851

BETAS CR -13.781066

BETAP CR 0.735757

BETAD CR -6.372908

ZS CR 2.838413

ZP CR 1.379560

ZD CR 1.188729

ZSN CR 2.174521

ZPN CR 4.770642

ZDN CR 2.141579

GSS CR 11.887886

GSP CR 14.518298

GPP CR 28.418564

GP2 CR 24.911643

HSP CR 1.187458

F0SD CR 7.511007

G2SD CR 2.622589

ALPB_H CR 1.710489

XFAC_H CR 0.451845

ALPB_LI CR 1.554282

XFAC_LI CR 1.523425

ALPB_C CR 2.200250

XFAC_C CR 0.723497

ALPB_N CR 1.978476

XFAC_N CR 0.431966

ALPB_O CR 2.226688

XFAC_O CR 0.603066

ALPB_F CR 2.545695

XFAC_F CR 0.581501

ALPB_NA CR 1.742438

XFAC_NA CR 7.141413

ALPB_MG CR 1.949255

XFAC_MG CR 9.004042

ALPB_SI CR 1.632536

XFAC_SI CR 1.831750

ALPB_P CR 0.965663

XFAC_P CR 0.488071

ALPB_S CR 2.022399

XFAC_S CR 0.610052

ALPB_CL CR 2.494604

XFAC_CL CR 0.987014

ALPB_K CR 1.827441

XFAC_K CR 14.122878

ALPB_CA CR 1.748419

XFAC_CA CR 3.971766

ALPB_CR CR 2.859778

XFAC_CR CR 21.294482

USS MN -42.374682

UPP MN -18.304981

UDD MN -54.430991

BETAS MN -19.986721

BETAP MN -51.153604

BETAD MN -28.049908

ZS MN 1.666440

ZP MN 2.078735

ZD MN 2.897070

ZSN MN 1.299761

ZPN MN 4.059245

ZDN MN 1.146085

GSS MN 7.105662

GSP MN 8.807648

GPP MN 24.180795

GP2 MN 21.196825

HSP MN 0.221872

F0SD MN 4.784190

G2SD MN 2.008311

ALPB_H MN 1.815287

XFAC_H MN 1.334984

ALPB_C MN 2.122570

XFAC_C MN 1.646822

ALPB_N MN 2.625097

XFAC_N MN 2.366982

ALPB_O MN 3.225970

XFAC_O MN 3.636943

ALPB_F MN 3.508953

XFAC_F MN 2.404476

ALPB_AL MN 1.231200

XFAC_AL MN 1.130368

ALPB_SI MN 1.881580

XFAC_SI MN 3.934609

ALPB_P MN 1.879268

XFAC_P MN 5.259289

ALPB_S MN 2.205580

XFAC_S MN 2.583375

ALPB_CL MN 2.275167

XFAC_CL MN 2.025304

ALPB_K MN 1.328545

XFAC_K MN 1.921563

ALPB_CA MN 1.298445

XFAC_CA MN 0.520488

ALPB_TI MN 1.633575

XFAC_TI MN 4.212201

ALPB_MN MN 2.502150

XFAC_MN MN 23.014869

USS FE -74.715611

UPP FE -56.758188

UDD FE -90.918476

BETAS FE -4.365430

BETAP FE -4.256080

BETAD FE -12.531631

ZS FE 1.157576

ZP FE 2.737621

ZD FE 1.860792

ZSN FE 2.223065

ZPN FE 1.314405

ZDN FE 1.769722

GSS FE 12.153271

GSP FE 8.511068

GPP FE 7.829869

GP2 FE 6.863644

HSP FE 1.267977

POC FE 0.993526

F0SD FE 9.314037

G2SD FE 1.970401

ALPB_H FE 2.325000

XFAC_H FE 0.797044

ALPB_C FE 2.439391

XFAC_C FE 0.840113

ALPB_N FE 2.710121

XFAC_N FE 1.307687

ALPB_O FE 2.977229

XFAC_O FE 1.669098

ALPB_F FE 3.266034

XFAC_F FE 1.572783

ALPB_SI FE 2.261269

XFAC_SI FE 1.302779

ALPB_P FE 1.425836

XFAC_P FE 0.597968

ALPB_S FE 2.922342

XFAC_S FE 3.055008

ALPB_CL FE 2.803764

XFAC_CL FE 1.475990

ALPB_K FE 0.914983

XFAC_K FE 0.471163

ALPB_TI FE 2.152071

XFAC_TI FE 1.718797

ALPB_CR FE 2.320197

XFAC_CR FE 1.605266

ALPB_FE FE 3.253806

XFAC_FE FE 25.101048

USS CO -37.720682

UPP CO -0.230340

UDD CO -85.185900

BETAS CO -11.175136

BETAP CO -18.331339

BETAD CO -5.935777

ZS CO 1.789441

ZP CO 1.531664

ZD CO 1.951497

ZSN CO 1.710796

ZPN CO 0.928007

ZDN CO 1.563753

GSS CO 9.352749

GSP CO 6.087093

GPP CO 5.528108

GP2 CO 4.845926

HSP CO 0.763102

POC CO 1.433458

F0SD CO 3.045500

G2SD CO 1.015102

ALPB_H CO 2.212022

XFAC_H CO 0.781287

ALPB_LI CO 1.930303

XFAC_LI CO 0.523612

ALPB_B CO 3.200000

XFAC_B CO 1.000000

ALPB_C CO 1.369735

XFAC_C CO 0.101941

ALPB_N CO 2.018692

XFAC_N CO 0.371117

ALPB_O CO 2.512985

XFAC_O CO 0.617937

ALPB_F CO 3.169014

XFAC_F CO 1.042929

ALPB_NA CO 1.130004

XFAC_NA CO 0.525429

ALPB_SI CO 2.247195

XFAC_SI CO 1.130253

ALPB_P CO 2.298868

XFAC_P CO 3.189088

ALPB_S CO 2.144853

XFAC_S CO 0.522339

ALPB_CL CO 2.604673

XFAC_CL CO 0.979572

ALPB_K CO 1.347379

XFAC_K CO 1.363649

ALPB_CR CO 1.965685

XFAC_CR CO 0.907585

ALPB_CO CO 1.072023

XFAC_CO CO 0.082968

USS NI -55.503570

UPP NI -30.601744

UDD NI -68.610896

BETAS NI -15.417178

BETAP NI -21.305796

BETAD NI -4.094535

ZS NI 1.708340

ZP NI 2.000099

ZD NI 5.698724

ZSN NI 1.177087

ZPN NI 1.013217

ZDN NI 1.017987

GSS NI 6.435016

GSP NI 5.921995

GPP NI 6.035702

GP2 NI 5.290881

HSP NI 1.379687

POC NI 2.208500

F0SD NI 5.492550

G2SD NI 2.469437

ALPB_H NI 1.921141

XFAC_H NI 0.694497

ALPB_B NI 2.332207

XFAC_B NI 0.529685

ALPB_C NI 2.135123

XFAC_C NI 0.429059

ALPB_N NI 2.259589

XFAC_N NI 0.403691

ALPB_O NI 2.452312

XFAC_O NI 0.284888

ALPB_F NI 3.145389

XFAC_F NI 0.559407

ALPB_SI NI 2.260625

XFAC_SI NI 3.024544

ALPB_P NI 1.646184

XFAC_P NI 0.793563

ALPB_S NI 2.360866

XFAC_S NI 0.923582

ALPB_CL NI 2.771621

XFAC_CL NI 1.509842

ALPB_K NI 1.110139

XFAC_K NI 0.642360

ALPB_CR NI 2.774356

XFAC_CR NI 29.999969

ALPB_NI NI 1.626235

XFAC_NI NI 0.339558

USS CU -55.174441

UPP CU 3.200458

UDD CU -118.258961

BETAS CU -11.801588

BETAP CU -37.165178

BETAD CU -14.652492

ZS CU 1.735325

ZP CU 3.219976

ZD CU 6.013523

ZSN CU 2.419271

ZPN CU 0.302125

ZDN CU 1.678203

GSS CU 13.225910

GSP CU 2.055274

GPP CU 1.799749

GP2 CU 1.577656

HSP CU 0.000420

F0SD CU 5.160900

G2SD CU 2.792359

ALPB_H CU 2.941555

XFAC_H CU 1.781622

ALPB_C CU 3.018944

XFAC_C CU 1.413488

ALPB_N CU 2.566300

XFAC_N CU 0.429906

ALPB_O CU 1.911057

XFAC_O CU 0.098068

ALPB_F CU 3.176529

XFAC_F CU 0.411293

ALPB_NA CU 1.306695

XFAC_NA CU 0.785487

ALPB_AL CU 2.320517

XFAC_AL CU 12.995965

ALPB_P CU 0.858794

XFAC_P CU 5.035151

ALPB_S CU 2.053844

XFAC_S CU 0.296518

ALPB_CL CU 2.475894

XFAC_CL CU 0.372668

ALPB_K CU 2.087357

XFAC_K CU 7.795310

ALPB_CU CU 3.103277

XFAC_CU CU 3.391704

USS ZN -16.700035

UPP ZN -14.844247

BETAS ZN -16.770975

BETAP ZN 2.907797

ZS ZN 1.560140

ZP ZN 1.915631

GSS ZN 6.421475

GSP ZN 10.243652

GPP ZN 20.001326

GP2 ZN 16.126802

HSP ZN 0.983644

ALPB_H ZN 1.874800

XFAC_H ZN 1.696831

ALPB_C ZN 2.171605

XFAC_C ZN 2.386580

ALPB_N ZN 1.805998

XFAC_N ZN 0.900539

ALPB_O ZN 2.079887

XFAC_O ZN 1.116990

ALPB_F ZN 1.859561

XFAC_F ZN 0.499581

ALPB_NA ZN 1.588584

XFAC_NA ZN 5.694720

ALPB_SI ZN 1.890360

XFAC_SI ZN 6.865738

ALPB_P ZN 1.398572

XFAC_P ZN 1.863594

ALPB_S ZN 1.379514

XFAC_S ZN 0.533478

ALPB_CL ZN 1.588143

XFAC_CL ZN 0.547720

ALPB_CA ZN 0.974041

XFAC_CA ZN 1.296565

ALPB_V ZN 1.513777

XFAC_V ZN 1.442270

ALPB_CR ZN 2.071878

XFAC_CR ZN 2.312424

ALPB_ZN ZN 1.998115

XFAC_ZN ZN 19.124599

USS GA -30.812730

UPP GA -22.498885

BETAS GA -15.082480

BETAP GA -0.938845

ZS GA 1.913326

ZP GA 1.811217

GSS GA 9.436450

GSP GA 9.189262

GPP GA 5.480436

GP2 GA 6.991064

HSP GA 0.970992

ALPB_H GA 2.170771

XFAC_H GA 2.091955

ALPB_C GA 2.188866

XFAC_C GA 1.617568

ALPB_N GA 1.949999

XFAC_N GA 0.867734

ALPB_O GA 2.408216

XFAC_O GA 1.379976

ALPB_F GA 3.055971

XFAC_F GA 2.319957

ALPB_SI GA 2.169690

XFAC_SI GA 5.031330

ALPB_P GA 1.600000

XFAC_P GA 4.000000

ALPB_S GA 2.514000

XFAC_S GA 4.204343

ALPB_CL GA 2.104228

XFAC_CL GA 1.129276

ALPB_GA GA 2.390223

XFAC_GA GA 11.941483

USS GE -35.694620

UPP GE -29.273804

BETAS GE -18.071730

BETAP GE -1.563157

ZS GE 2.762845

ZP GE 1.531131

GSS GE 4.991616

GSP GE 9.108444

GPP GE 6.693916

GP2 GE 5.914950

HSP GE 0.801760

ALPB_H GE 2.470301

XFAC_H GE 2.398259

ALPB_C GE 2.351577

XFAC_C GE 1.605487

ALPB_N GE 2.239698

XFAC_N GE 1.028521

ALPB_O GE 2.217395

XFAC_O GE 0.690557

ALPB_F GE 1.727325

XFAC_F GE 0.165644

ALPB_SI GE 2.053934

XFAC_SI GE 3.121907

ALPB_P GE 1.831652

XFAC_P GE 4.212771

ALPB_S GE 2.358433

XFAC_S GE 1.947726

ALPB_CL GE 2.506796

XFAC_CL GE 1.783333

ALPB_MN GE 1.937769

XFAC_MN GE 2.470135

ALPB_CO GE 2.852610

XFAC_CO GE 2.151850

ALPB_GE GE 2.215455

XFAC_GE GE 5.884206

USS AS -41.523302

UPP AS -36.959219

UDD AS -35.859071

BETAS AS -17.440295

BETAP AS -6.603566

BETAD AS -2.445554

ZS AS 3.213850

ZP AS 1.628384

ZD AS 3.314358

ZSN AS 0.916221

ZPN AS 1.115722

ZDN AS 2.137809

GSS AS 8.088350

GSP AS 7.457692

GPP AS 8.918517

GP2 AS 7.102449

HSP AS 0.969630

ALPB_H AS 1.749762

XFAC_H AS 0.763924

ALPB_C AS 1.805305

XFAC_C AS 0.604465

ALPB_N AS 2.035339

XFAC_N AS 0.784041

ALPB_O AS 2.387990

XFAC_O AS 1.076670

ALPB_F AS 2.783517

XFAC_F AS 1.196884

ALPB_NA AS 1.763497

XFAC_NA AS 2.673425

ALPB_AL AS 1.332670

XFAC_AL AS 1.322056

ALPB_SI AS 1.771030

XFAC_SI AS 1.384298

ALPB_S AS 1.826372

XFAC_S AS 0.732648

ALPB_CL AS 1.947927

XFAC_CL AS 0.747436

ALPB_K AS 1.267957

XFAC_K AS 2.276204

ALPB_TI AS 1.711955

XFAC_TI AS 1.371503

ALPB_CO AS 1.514923

XFAC_CO AS 2.030232

ALPB_ZN AS 1.618734

XFAC_ZN AS 2.700385

ALPB_GA AS 1.534812

XFAC_GA AS 1.196640

ALPB_AS AS 1.707277

XFAC_AS AS 1.325873

USS SE -47.218303

UPP SE -35.588294

BETAS SE -9.614158

BETAP SE -6.121302

ZS SE 2.751130

ZP SE 1.901764

GSS SE 4.895424

GSP SE 6.792977

GPP SE 5.775063

GP2 SE 5.578480

HSP SE 3.152775

ALPB_H SE 2.547234

XFAC_H SE 1.229099

ALPB_C SE 2.186857

XFAC_C SE 0.654796

ALPB_N SE 1.980885

XFAC_N SE 0.448537

ALPB_O SE 2.612643

XFAC_O SE 0.860233

ALPB_F SE 2.463196

XFAC_F SE 0.473969

ALPB_NA SE 1.115555

XFAC_NA SE 0.902628

ALPB_SI SE 2.318601

XFAC_SI SE 2.051717

ALPB_P SE 1.865719

XFAC_P SE 2.359419

ALPB_S SE 1.492756

XFAC_S SE 0.530796

ALPB_CL SE 2.170000

XFAC_CL SE 0.869163

ALPB_K SE 1.680151

XFAC_K SE 3.871380

ALPB_MN SE 1.981410

XFAC_MN SE 2.170787

ALPB_CO SE 2.523450

XFAC_CO SE 2.202410

ALPB_ZN SE 1.163289

XFAC_ZN SE 0.367711

ALPB_GE SE 1.604107

XFAC_GE SE 0.556002

ALPB_AS SE 1.514823

XFAC_AS SE 0.541956

ALPB_SE SE 1.524158

XFAC_SE SE 0.334506

USS BR -49.141354

UPP BR -48.274409

UDD BR 2.677328

BETAS BR -32.458894

BETAP BR -10.270309

BETAD BR -19.977175

ZS BR 3.725480

ZP BR 2.242318

ZD BR 1.591034

ZSN BR 10.522069

ZPN BR 9.531017

ZDN BR 5.776829

GSS BR 8.131665

GSP BR 4.285572

GPP BR 8.056519

GP2 BR 7.520115

HSP BR 1.567275

ALPB_H BR 2.339252

XFAC_H BR 1.270390

ALPB_HE BR 2.127598

XFAC_HE BR 1.062013

ALPB_LI BR 2.143819

XFAC_LI BR 2.241404

ALPB_BE BR 2.283569

XFAC_BE BR 2.659130

ALPB_B BR 2.307098

XFAC_B BR 1.849590

ALPB_C BR 2.252349

XFAC_C BR 0.968921

ALPB_N BR 3.015469

XFAC_N BR 4.148435

ALPB_O BR 2.739280

XFAC_O BR 1.425004

ALPB_F BR 2.756179

XFAC_F BR 0.915922

ALPB_NE BR 2.483203

XFAC_NE BR 1.001506

ALPB_NA BR 2.327183

XFAC_NA BR 11.511433

ALPB_MG BR 2.350023

XFAC_MG BR 7.183008

ALPB_AL BR 1.514344

XFAC_AL BR 1.504277

ALPB_SI BR 1.715815

XFAC_SI BR 1.126731

ALPB_P BR 1.664343

XFAC_P BR 1.746224

ALPB_S BR 2.099922

XFAC_S BR 1.004759

ALPB_CL BR 1.906403

XFAC_CL BR 0.581542

ALPB_AR BR 2.454724

XFAC_AR BR 3.261699

ALPB_K BR 1.887799

XFAC_K BR 7.523969

ALPB_CA BR 2.558257

XFAC_CA BR 12.875179

ALPB_SC BR 1.531278

XFAC_SC BR 1.063920

ALPB_TI BR 1.760015

XFAC_TI BR 1.534465

ALPB_V BR 1.909502

XFAC_V BR 1.394543

ALPB_CR BR 1.781866

XFAC_CR BR 0.746857

ALPB_MN BR 2.183298

XFAC_MN BR 2.530861

ALPB_FE BR 2.388196

XFAC_FE BR 1.415413

ALPB_CO BR 2.124301

XFAC_CO BR 0.759690

ALPB_NI BR 2.543159

XFAC_NI BR 1.656698

ALPB_CU BR 3.040037

XFAC_CU BR 2.655465

ALPB_ZN BR 1.594962

XFAC_ZN BR 1.241996

ALPB_GA BR 1.934418

XFAC_GA BR 1.722754

ALPB_GE BR 2.062366

XFAC_GE BR 2.031652

ALPB_AS BR 1.750449

XFAC_AS BR 0.949379

ALPB_SE BR 1.788806

XFAC_SE BR 0.682982

ALPB_BR BR 2.147378

XFAC_BR BR 1.599562

USS KR 8.535384

UPP KR -80.484321

BETAS KR -2.727088

BETAP KR -16.142951

ZS KR 1.312248

ZP KR 4.491371

GSS KR 19.999857

GSP KR 1.175304

GPP KR 9.174784

GP2 KR 14.926948

HSP KR 0.299867

ALPB_H KR 3.770453

XFAC_H KR 5.125897

ALPB_HE KR 1.996943

XFAC_HE KR 0.627701

ALPB_LI KR 3.004783

XFAC_LI KR 8.377143

ALPB_BE KR 3.289764

XFAC_BE KR 10.264026

ALPB_B KR 2.559201

XFAC_B KR 2.931148

ALPB_C KR 2.076738

XFAC_C KR 0.652623

ALPB_N KR 1.644052

XFAC_N KR 0.199606

ALPB_O KR 0.297001

ALPB_F KR 3.452321

XFAC_F KR 4.134407

ALPB_NE KR 2.813679

XFAC_NE KR 1.433722

ALPB_NA KR 2.562062

XFAC_NA KR 9.817859

ALPB_MG KR 1.296221

XFAC_MG KR 1.119449

ALPB_AL KR 2.493834

XFAC_AL KR 5.076857

ALPB_SI KR 1.545354

XFAC_SI KR 0.639030

ALPB_CL KR 1.884662

XFAC_CL KR 0.520353

ALPB_AR KR 1.995125

XFAC_AR KR 0.554874

ALPB_K KR 2.296640

XFAC_K KR 8.532309

ALPB_CA KR 1.559229

XFAC_CA KR 1.305808

ALPB_BR KR 1.608300

XFAC_BR KR 0.499653

ALPB_KR KR 1.913342

XFAC_KR KR 0.252431

USS RB -4.120962

UPP RB -0.633300

BETAS RB -8.442947

BETAP RB 4.853952

ZS RB 1.314831

ZP RB 6.015581

GSS RB 11.892047

GSP RB 3.477383

GPP RB 6.000901

GP2 RB 6.008182

HSP RB 0.998126

ALPB_H RB 1.890495

XFAC_H RB 4.316836

ALPB_HE RB 1.543436

XFAC_HE RB 1.804024

ALPB_B RB 2.989999

XFAC_B RB 10.280532

ALPB_C RB 2.287377

XFAC_C RB 24.603216

ALPB_N RB 2.205212

XFAC_N RB 19.919815

ALPB_O RB 1.572166

XFAC_O RB 0.791546

ALPB_F RB 3.131045

XFAC_F RB 2.629683

ALPB_NE RB 2.429710

XFAC_NE RB 7.683406

ALPB_AL RB 0.931060

XFAC_AL RB 19.138062

ALPB_P RB 0.922429

XFAC_P RB 0.526907

ALPB_S RB 1.285680

XFAC_S RB 1.380751

ALPB_CL RB 1.349244

XFAC_CL RB 0.714916

ALPB_AR RB 2.581073

XFAC_AR RB 18.431817

ALPB_K RB 1.719577

XFAC_K RB 1.174003

ALPB_V RB 2.024277

XFAC_V RB 12.360809

ALPB_CR RB 1.569973

XFAC_CR RB 5.846691

ALPB_BR RB 1.737339

XFAC_BR RB 7.887658

ALPB_KR RB 2.413547

XFAC_KR RB 15.315026

ALPB_RB RB 0.684616

XFAC_RB RB 4.280624

USS SR -10.693066

UPP SR -8.539218

BETAS SR -4.904378

BETAP SR 8.809297

ZS SR 2.092264

ZP SR 3.314082

GSS SR 6.494973

GSP SR 4.045506

GPP SR 2.547611

GP2 SR 4.121201

HSP SR 1.102790

ALPB_H SR 2.918082

XFAC_H SR 18.457472

ALPB_C SR 2.802150

XFAC_C SR 11.414713

ALPB_N SR 3.025117

XFAC_N SR 14.103810

ALPB_O SR 3.921499

XFAC_O SR 17.067149

ALPB_F SR 3.309755

XFAC_F SR 3.758667

ALPB_SI SR 2.416117

XFAC_SI SR 29.996030

ALPB_P SR 2.273841

XFAC_P SR 23.946650

ALPB_S SR 3.325295

XFAC_S SR 41.563327

ALPB_CL SR 3.501974

XFAC_CL SR 39.960719

ALPB_TI SR 2.880030

XFAC_TI SR 2.817250

ALPB_GA SR 1.489463

XFAC_GA SR 2.800419

ALPB_BR SR 3.086374

XFAC_BR SR 19.218824

ALPB_SR SR 2.194036

XFAC_SR SR 31.817350

USS Y -17.035117

UPP Y -16.168689

UDD Y -16.354811

BETAS Y -10.513848

BETAP Y -11.341408

BETAD Y -10.701025

ZS Y 1.605083

ZP Y 2.131069

ZD Y 6.021645

ZSN Y 1.186263

ZPN Y 2.244351

ZDN Y 0.911477

GSS Y 5.318448

GSP Y 6.318665

GPP Y 11.005171

GP2 Y 9.590777

HSP Y 0.637634

POC Y 2.019557

F0SD Y 6.855595

G2SD Y 5.889822

ALPB_H Y 2.322175

XFAC_H Y 6.935667

ALPB_LI Y 1.212009

XFAC_LI Y 0.577598

ALPB_C Y 2.541211

XFAC_C Y 19.957240

ALPB_N Y 2.084245

XFAC_N Y 3.253368

ALPB_O Y 2.086475

XFAC_O Y 1.424444

ALPB_F Y 3.245964

XFAC_F Y 9.257528

ALPB_AL Y 1.003500

XFAC_AL Y 0.500670

ALPB_SI Y 2.016820

XFAC_SI Y 3.219030

ALPB_P Y 1.172165

XFAC_P Y 1.726458

ALPB_S Y 1.345475

XFAC_S Y 0.961448

ALPB_CL Y 1.882700

XFAC_CL Y 2.186706

ALPB_K Y 0.947193

XFAC_K Y 1.143281

ALPB_BR Y 1.359600

XFAC_BR Y 1.090173

ALPB_Y Y 1.533049

XFAC_Y Y 15.620872

USS ZR -18.679203

UPP ZR -0.049727

UDD ZR -23.299157

BETAS ZR 1.688872

BETAP ZR 2.330045

BETAD ZR -4.552268

ZS ZR 1.373517

ZP ZR 1.141705

ZD ZR 1.618769

ZSN ZR 1.082243

ZPN ZR 2.978817

ZDN ZR 1.417227

GSS ZR 4.852089

GSP ZR 5.870299

GPP ZR 14.606623

GP2 ZR 12.729368

HSP ZR 0.151363

F0SD ZR 4.737734

G2SD ZR 2.141620

ALPB_H ZR 1.536594

XFAC_H ZR 0.414278

ALPB_C ZR 1.738320

XFAC_C ZR 0.715392

ALPB_N ZR 1.986255

XFAC_N ZR 1.386137

ALPB_O ZR 2.093741

XFAC_O ZR 1.293822

ALPB_F ZR 2.406399

XFAC_F ZR 1.606098

ALPB_AL ZR 1.270620

XFAC_AL ZR 0.874060

ALPB_SI ZR 1.605795

XFAC_SI ZR 1.566491

ALPB_P ZR 0.963910

XFAC_P ZR 1.020015

ALPB_S ZR 0.957666

XFAC_S ZR 0.200522

ALPB_CL ZR 2.352409

XFAC_CL ZR 2.273630

ALPB_BR ZR 1.617591

XFAC_BR ZR 1.243772

ALPB_ZR ZR 2.714671

XFAC_ZR ZR 29.768192

USS NB -33.497110

UPP NB -34.762698

UDD NB -44.819149

BETAS NB -23.566737

BETAP NB -1.623945

BETAD NB -7.421668

ZS NB 2.761686

ZP NB 5.999062

ZD NB 1.611677

ZSN NB 1.429235

ZPN NB 2.911794

ZDN NB 1.950434

GSS NB 6.407780

GSP NB 7.659444

GPP NB 14.277976

GP2 NB 12.442959

HSP NB 0.619230

F0SD NB 6.393769

G2SD NB 1.759636

ALPB_H NB 2.321651

XFAC_H NB 6.958727

ALPB_C NB 2.277928

XFAC_C NB 1.991488

ALPB_N NB 2.810017

XFAC_N NB 4.374260

ALPB_O NB 2.715670

XFAC_O NB 2.793681

ALPB_F NB 3.115376

XFAC_F NB 3.297982

ALPB_NA NB 2.551010

XFAC_NA NB 8.276020

ALPB_P NB 1.922968

XFAC_P NB 6.219347

ALPB_S NB 2.279550

XFAC_S NB 3.225637

ALPB_CL NB 2.757523

XFAC_CL NB 6.452483

ALPB_K NB 4.521360

XFAC_K NB 2.026590

ALPB_BR NB 2.531918

XFAC_BR NB 8.316457

ALPB_NB NB 2.030464

XFAC_NB NB 10.027153

USS MO -51.662768

UPP MO 46.059429

UDD MO -57.269405

BETAS MO 6.685073

BETAP MO 5.485123

BETAD MO -13.146960

ZS MO 1.595399

ZP MO 1.426575

ZD MO 1.787748

ZSN MO 1.903541

ZPN MO 1.592195

ZDN MO 1.889678

GSS MO 8.534266

GSP MO 7.704937

GPP MO 7.807325

GP2 MO 6.803921

HSP MO 1.787407

F0SD MO 9.654475

G2SD MO 2.314954

ALPB_H MO 2.139004

XFAC_H MO 1.177934

ALPB_LI MO 2.201335

XFAC_LI MO 5.209247

ALPB_C MO 2.140063

XFAC_C MO 1.042667

ALPB_N MO 2.293955

XFAC_N MO 1.330858

ALPB_O MO 2.197353

XFAC_O MO 0.864597

ALPB_F MO 2.593518

XFAC_F MO 1.107779

ALPB_NA MO 2.440770

XFAC_NA MO 8.286550

ALPB_P MO 1.850441

XFAC_P MO 1.522846

ALPB_S MO 2.343350

XFAC_S MO 1.822187

ALPB_CL MO 2.358706

XFAC_CL MO 1.570824

ALPB_K MO 1.594941

XFAC_K MO 10.522232

ALPB_CR MO 1.873232

XFAC_CR MO 0.484096

ALPB_FE MO 2.239581

XFAC_FE MO 3.257115

ALPB_BR MO 1.934589

XFAC_BR MO 1.291525

ALPB_RB MO 2.971399

XFAC_RB MO 0.874676

ALPB_MO MO 1.078447

XFAC_MO MO 0.223956

USS TC -48.916740

UPP TC -21.908166

UDD TC -53.807590

BETAS TC -17.096185

BETAP TC -17.740652

BETAD TC -7.241592

ZS TC 2.104672

ZP TC 2.669984

ZD TC 3.030496

ZSN TC 2.061082

ZPN TC 0.888524

ZDN TC 1.575315

GSS TC 9.240580

GSP TC 4.795964

GPP TC 4.356876

GP2 TC 3.796926

HSP TC 0.248174

F0SD TC 7.521148

G2SD TC 3.106149

ALPB_H TC 2.576199

XFAC_H TC 5.418951

ALPB_C TC 2.815972

XFAC_C TC 3.999428

ALPB_N TC 2.177956

XFAC_N TC 0.980071

ALPB_O TC 2.535619

XFAC_O TC 1.303538

ALPB_F TC 3.385092

XFAC_F TC 3.880884

ALPB_P TC 0.930051

XFAC_P TC 0.470758

ALPB_S TC 2.141702

XFAC_S TC 1.449910

ALPB_CL TC 2.360242

XFAC_CL TC 1.744657

ALPB_GE TC 2.852820

XFAC_GE TC 2.152060

ALPB_SE TC 2.523660

XFAC_SE TC 2.202620

ALPB_BR TC 2.688330

XFAC_BR TC 6.426037

ALPB_TC TC 2.153000

XFAC_TC TC 2.572063

USS RU -41.151429

UPP RU -42.965344

UDD RU -45.714719

BETAS RU -4.989393

BETAP RU -10.778690

BETAD RU 1.216566

ZS RU 1.605646

ZP RU 4.580820

ZD RU 1.244578

ZSN RU 1.172546

ZPN RU 1.373361

ZDN RU 1.018114

GSS RU 5.256950

GSP RU 5.631870

GPP RU 6.734273

GP2 RU 5.868779

HSP RU 1.326657

F0SD RU 4.898881

G2SD RU 2.648488

ALPB_H RU 3.031003

XFAC_H RU 0.462490

ALPB_C RU 2.661734

XFAC_C RU 0.434352

ALPB_N RU 1.951233

XFAC_N RU 0.271221

ALPB_O RU 1.928484

XFAC_O RU 0.339590

ALPB_F RU 2.719488

XFAC_F RU 0.680978

ALPB_SI RU 2.775910

XFAC_SI RU 0.849430

ALPB_P RU 1.440298

XFAC_P RU 0.482587

ALPB_S RU 3.002139

XFAC_S RU 0.788319

ALPB_CL RU 3.340740

XFAC_CL RU 1.986295

ALPB_GE RU 2.852320

XFAC_GE RU 2.151560

ALPB_SE RU 2.523160

XFAC_SE RU 2.202120

ALPB_BR RU 2.611647

XFAC_BR RU 3.893512

ALPB_RU RU 2.341541

XFAC_RU RU 0.984874

USS RH -24.613157

UPP RH 6.621039

UDD RH -81.764165

BETAS RH -9.488908

BETAP RH -6.699556

BETAD RH -7.997845

ZS RH 1.591465

ZP RH 4.546046

ZD RH 2.685918

ZSN RH 2.079986

ZPN RH 9.641003

ZDN RH 1.787794

GSS RH 9.325333

GSP RH 11.318440

GPP RH 47.274639

GP2 RH 41.198863

HSP RH 0.017585

F0SD RH 2.230584

G2SD RH 1.492841

ALPB_H RH 2.716287

XFAC_H RH 1.728302

ALPB_B RH 2.400000

XFAC_B RH 2.000000

ALPB_C RH 3.007700

XFAC_C RH 0.562962

ALPB_N RH 3.028135

XFAC_N RH 1.013618

ALPB_O RH 3.452408

XFAC_O RH 1.534037

ALPB_F RH 3.083507

XFAC_F RH 0.772245

ALPB_SI RH 2.776490

XFAC_SI RH 0.850010

ALPB_P RH 2.236601

XFAC_P RH 0.738916

ALPB_S RH 3.005420

XFAC_S RH 0.970563

ALPB_CL RH 3.542676

XFAC_CL RH 0.628186

ALPB_GE RH 2.852900

XFAC_GE RH 2.152140

ALPB_SE RH 2.523740

XFAC_SE RH 2.202700

ALPB_BR RH 2.893677

XFAC_BR RH 1.441509

ALPB_RH RH 3.281577

XFAC_RH RH 17.154616

USS PD -90.670356

UPP PD 45.018147

UDD PD -94.618031

BETAS PD -18.862423

BETAP PD -18.107010

BETAD PD -3.592862

ZS PD 5.790768

ZP PD 2.169788

ZD PD 1.327661

ZSN PD 1.985663

ZPN PD 0.621281

ZDN PD 1.768258

GSS PD 8.902449

GSP PD 3.376439

GPP PD 3.046450

GP2 PD 2.654918

HSP PD 0.043028

F0SD PD 9.251409

G2SD PD 1.948722

ALPB_H PD 3.052992

XFAC_H PD 0.675244

ALPB_C PD 1.449994

XFAC_C PD 0.040769

ALPB_N PD 2.319285

XFAC_N PD 0.327063

ALPB_O PD 2.362481

XFAC_O PD 0.394849

ALPB_F PD 3.117188

XFAC_F PD 0.610235

ALPB_AL PD 1.572720

XFAC_AL PD 1.057290

ALPB_SI PD 2.714212

XFAC_SI PD 1.381243

ALPB_P PD 0.876896

XFAC_P PD 0.223289

ALPB_S PD 3.134436

XFAC_S PD 0.568359

ALPB_CL PD 2.966363

XFAC_CL PD 0.764165

ALPB_BR PD 2.087790

XFAC_BR PD 0.491172

ALPB_PD PD 1.712149

XFAC_PD PD 0.297913

USS AG -92.280499

UPP AG 29.229985

UDD AG -82.344865

BETAS AG -9.850776

BETAP AG -29.894728

BETAD AG -63.636331

ZS AG 1.793032

ZP AG 2.528721

ZD AG 3.524808

ZSN AG 1.619764

ZPN AG 0.439729

ZDN AG 1.210202

GSS AG 7.261991

GSP AG 2.391732

GPP AG 2.156210

GP2 AG 1.879092

HSP AG 0.014435

F0SD AG 8.987758

G2SD AG 4.716654

ALPB_H AG 1.866268

XFAC_H AG 0.669745

ALPB_B AG 1.454270

XFAC_B AG 2.733745

ALPB_C AG 2.401775

XFAC_C AG 1.108319

ALPB_N AG 2.835438

XFAC_N AG 1.090232

ALPB_O AG 2.453629

XFAC_O AG 0.372450

ALPB_F AG 3.119532

XFAC_F AG 0.897783

ALPB_AL AG 1.683750

XFAC_AL AG 1.093559

ALPB_P AG 1.305572

XFAC_P AG 0.482631

ALPB_S AG 2.575670

XFAC_S AG 1.817701

ALPB_CL AG 3.198107

XFAC_CL AG 3.386746

ALPB_K AG 2.092259

XFAC_K AG 5.619211

ALPB_CR AG 2.700428

XFAC_CR AG 21.083639

ALPB_BR AG 3.259287

XFAC_BR AG 6.111850

ALPB_PD AG 4.000000

XFAC_PD AG 2.000000

ALPB_AG AG 1.489404

XFAC_AG AG 0.178879

USS CD -18.127987

UPP CD -13.777839

BETAS CD -23.781665

BETAP CD -11.892060

ZS CD 3.670047

ZP CD 1.857036

GSS CD 8.904816

GSP CD 9.232666

GPP CD 11.103045

GP2 CD 10.905897

HSP CD 0.981926

ALPB_H CD 1.875490

XFAC_H CD 3.377913

ALPB_C CD 1.940388

XFAC_C CD 3.855359

ALPB_N CD 1.769441

XFAC_N CD 1.481460

ALPB_O CD 2.668165

XFAC_O CD 5.349517

ALPB_F CD 3.174783

XFAC_F CD 8.351869

ALPB_NA CD 2.000000

XFAC_NA CD 6.000000

ALPB_SI CD 1.286882

XFAC_SI CD 2.345912

ALPB_S CD 1.735391

XFAC_S CD 2.929257

ALPB_CL CD 1.870170

XFAC_CL CD 2.254752

ALPB_K CD 1.033580

XFAC_K CD 2.093242

ALPB_SE CD 1.881368

XFAC_SE CD 6.139995

ALPB_BR CD 1.918455

XFAC_BR CD 5.550415

ALPB_CD CD 1.428097

XFAC_CD CD 10.662907

USS IN -26.891944

UPP IN -28.519053

BETAS IN -0.447307

BETAP IN -4.269337

ZS IN 1.902085

ZP IN 1.940127

GSS IN 6.493621

GSP IN 12.576468

GPP IN 10.282533

GP2 IN 10.903195

HSP IN 2.133796

ALPB_H IN 1.852461

XFAC_H IN 1.773147

ALPB_B IN 1.735480

XFAC_B IN 1.951651

ALPB_C IN 1.810115

XFAC_C IN 1.041540

ALPB_N IN 2.052217

XFAC_N IN 1.529722

ALPB_O IN 2.178110

XFAC_O IN 1.467957

ALPB_F IN 2.319418

XFAC_F IN 1.018315

ALPB_S IN 2.430104

XFAC_S IN 4.796933

ALPB_CL IN 2.211880

XFAC_CL IN 2.224354

ALPB_GA IN 1.596053

XFAC_GA IN 2.473577

ALPB_AS IN 1.520977

XFAC_AS IN 1.375570

ALPB_SE IN 1.362364

XFAC_SE IN 0.598029

ALPB_BR IN 1.862313

XFAC_BR IN 2.138634

ALPB_RB IN 0.859259

XFAC_RB IN 4.688357

ALPB_IN IN 2.601789

XFAC_IN IN 24.204383

USS SN -33.880164

UPP SN -39.128186

BETAS SN 0.443105

BETAP SN -8.486074

ZS SN 1.959238

ZP SN 1.976146

GSS SN 6.196917

GSP SN 10.595744

GPP SN 14.691065

GP2 SN 13.501111

HSP SN 1.234523

ALPB_H SN 1.855042

XFAC_H SN 1.459969

ALPB_C SN 1.818782

XFAC_C SN 0.961947

ALPB_N SN 1.783560

XFAC_N SN 0.731228

ALPB_O SN 1.959102

XFAC_O SN 0.723272

ALPB_F SN 2.593459

XFAC_F SN 1.477352

ALPB_AL SN 1.597939

XFAC_AL SN 2.367990

ALPB_S SN 2.065722

XFAC_S SN 1.909070

ALPB_CL SN 1.887044

XFAC_CL SN 0.944374

ALPB_K SN 2.238329

XFAC_K SN 3.440153

ALPB_GE SN 2.016055

XFAC_GE SN 3.500376

ALPB_SE SN 1.393411

XFAC_SE SN 0.413851

ALPB_BR SN 1.594297

XFAC_BR SN 0.954605

ALPB_SN SN 1.045406

XFAC_SN SN 0.300460

USS SB -42.835901

UPP SB -19.996258

UDD SB -20.317174

BETAS SB -13.037071

BETAP SB -6.166480

BETAD SB -9.740725

ZS SB 1.998600

ZP SB 1.887062

ZD SB 1.475516

ZSN SB 2.179206

ZPN SB 0.862318

ZDN SB 4.147596

GSS SB 9.994149

GSP SB 1.434008

GPP SB 7.208157

GP2 SB 6.212730

HSP SB 3.566032

ALPB_H SB 1.091035

XFAC_H SB 0.408876

ALPB_C SB 1.240714

XFAC_C SB 0.327493

ALPB_N SB 0.846645

XFAC_N SB 0.137604

ALPB_O SB 1.462059

XFAC_O SB 0.346536

ALPB_F SB 1.622505

XFAC_F SB 0.283768

ALPB_NA SB 1.106800

XFAC_NA SB 0.547287

ALPB_AL SB 1.085906

XFAC_AL SB 1.291895

ALPB_SI SB 2.519702

XFAC_SI SB 8.707039

ALPB_S SB 1.016407

XFAC_S SB 0.211102

ALPB_CL SB 1.170710

XFAC_CL SB 0.217072

ALPB_MN SB 1.698753

XFAC_MN SB 2.384408

ALPB_CO SB 2.204630

XFAC_CO SB 2.276050

ALPB_BR SB 1.227775

XFAC_BR SB 0.567204

ALPB_TC SB 2.204850

XFAC_TC SB 2.276260

ALPB_RU SB 2.968084

XFAC_RU SB 2.509269

ALPB_RH SB 2.204930

XFAC_RH SB 2.276340

ALPB_IN SB 1.011173

XFAC_IN SB 0.470521

ALPB_SB SB 0.657753

XFAC_SB SB 0.219843

USS TE -97.416118

UPP TE -50.000552

BETAS TE -70.028904

BETAP TE -11.183348

ZS TE 3.024819

ZP TE 2.598283

GSS TE 18.350494

GSP TE 11.255114

GPP TE 8.695261

GP2 TE 7.622556

HSP TE 3.626912

ALPB_H TE 2.879705

XFAC_H TE 7.645321

ALPB_B TE 2.443355

XFAC_B TE 2.926026

ALPB_C TE 2.858205

XFAC_C TE 7.513380

ALPB_N TE 2.548060

XFAC_N TE 2.356842

ALPB_O TE 2.359294

XFAC_O TE 1.147602

ALPB_F TE 3.109030

XFAC_F TE 2.199214

ALPB_AL TE 1.783994

XFAC_AL TE 9.305330

ALPB_P TE 1.482343

XFAC_P TE 1.459960

ALPB_S TE 2.969323

XFAC_S TE 14.279019

ALPB_CL TE 1.475730

XFAC_CL TE 0.514830

ALPB_K TE 1.257635

XFAC_K TE 2.073198

ALPB_ZN TE 1.704782

XFAC_ZN TE 4.125260

ALPB_GE TE 2.049526

XFAC_GE TE 7.601044

ALPB_AS TE 1.275249

XFAC_AS TE 0.866529

ALPB_SE TE 1.585819

XFAC_SE TE 1.322800

ALPB_BR TE 2.316655

XFAC_BR TE 4.158560

ALPB_CD TE 1.759718

XFAC_CD TE 8.405812

ALPB_IN TE 1.913212

XFAC_IN TE 9.943252

ALPB_SN TE 2.265433

XFAC_SN TE 11.004064

ALPB_SB TE 1.634994

XFAC_SB TE 0.575666

ALPB_TE TE 3.032862

XFAC_TE TE 29.604279

USS I -63.618928

UPP I -45.760969

UDD I 6.109119

BETAS I -37.373318

BETAP I -10.174482

BETAD I -11.807267

ZS I 3.316202

ZP I 2.449124

ZD I 1.716121

ZSN I 4.000764

ZPN I 3.993847

ZDN I 3.946706

GSS I 7.658717

GSP I 8.237228

GPP I 5.667030

GP2 I 5.661068

HSP I 2.688576

ALPB_H I 2.303917

XFAC_H I 2.456367

ALPB_HE I 2.264096

XFAC_HE I 2.613098

ALPB_LI I 1.392191

XFAC_LI I 1.220335

ALPB_BE I 2.137694

XFAC_BE I 4.012926

ALPB_B I 1.949150

XFAC_B I 1.926808

ALPB_C I 2.148076

XFAC_C I 1.536813

ALPB_N I 2.204300

XFAC_N I 1.197247

ALPB_O I 2.031236

XFAC_O I 0.673908

ALPB_F I 2.168508

XFAC_F I 0.518622

ALPB_NE I 2.572520

XFAC_NE I 1.449278

ALPB_NA I 1.999781

XFAC_NA I 12.909796

ALPB_MG I 1.832289

XFAC_MG I 4.415343

ALPB_AL I 1.515624

XFAC_AL I 2.691541

ALPB_SI I 1.472015

XFAC_SI I 1.272495

ALPB_P I 1.560276

XFAC_P I 2.308251

ALPB_S I 2.108468

XFAC_S I 1.287638

ALPB_CL I 1.674480

XFAC_CL I 0.582734

ALPB_AR I 1.583967

XFAC_AR I 0.297828

ALPB_K I 1.527318

XFAC_K I 6.255639

ALPB_CA I 1.931292

XFAC_CA I 5.485613

ALPB_SC I 1.888645

XFAC_SC I 4.305507

ALPB_TI I 1.569430

XFAC_TI I 2.273746

ALPB_V I 1.204771

XFAC_V I 0.566891

ALPB_CR I 1.505878

XFAC_CR I 0.754833

ALPB_MN I 1.920970

XFAC_MN I 2.239969

ALPB_FE I 1.995455

XFAC_FE I 1.244120

ALPB_CO I 2.394155

XFAC_CO I 3.145732

ALPB_NI I 2.491283

XFAC_NI I 3.452112

ALPB_CU I 3.049738

XFAC_CU I 5.342329

ALPB_ZN I 1.785943

XFAC_ZN I 4.270507

ALPB_GA I 1.903558

XFAC_GA I 3.519264

ALPB_GE I 1.431330

XFAC_GE I 0.946363

ALPB_AS I 1.454624

XFAC_AS I 0.863506

ALPB_SE I 1.464103

XFAC_SE I 0.509254

ALPB_BR I 1.793757

XFAC_BR I 1.192163

ALPB_KR I 1.242469

XFAC_KR I 0.195416

ALPB_RB I 0.893509

XFAC_RB I 0.753057

ALPB_SR I 2.702289

XFAC_SR I 32.561240

ALPB_Y I 1.443236

XFAC_Y I 2.307839

ALPB_ZR I 1.402802

XFAC_ZR I 1.833851

ALPB_NB I 2.001333

XFAC_NB I 4.678302

ALPB_MO I 2.042051

XFAC_MO I 3.618240

ALPB_TC I 2.576693

XFAC_TC I 9.860653

ALPB_RU I 1.432008

XFAC_RU I 0.552218

ALPB_RH I 2.347687

XFAC_RH I 1.588054

ALPB_PD I 1.720521

XFAC_PD I 0.587118

ALPB_AG I 2.959757

XFAC_AG I 9.538157

ALPB_CD I 1.751947

XFAC_CD I 6.820820

ALPB_IN I 1.830626

XFAC_IN I 4.302750

ALPB_SN I 2.479003

XFAC_SN I 24.450811

ALPB_SB I 1.114193

XFAC_SB I 0.767547

ALPB_TE I 2.102109

XFAC_TE I 4.751442

ALPB_I I 1.619225

XFAC_I I 1.278518

USS XE -18.964330

UPP XE -108.181436

BETAS XE -2.718707

BETAP XE -44.936370

ZS XE 3.208788

ZP XE 2.727979

GSS XE 17.906443

GSP XE 4.106228

GPP XE 1.716979

GP2 XE 18.971469

HSP XE 4.990194

ALPB_H XE 1.356861

XFAC_H XE 0.701016

ALPB_HE XE 2.497832

XFAC_HE XE 2.599471

ALPB_LI XE 1.697716

XFAC_LI XE 4.467048

ALPB_BE XE 6.000011

XFAC_BE XE 0.654334

ALPB_B XE 3.233962

XFAC_B XE 1.995594

ALPB_C XE 1.704440

XFAC_C XE 0.826727

ALPB_N XE 1.932952

XFAC_N XE 0.925624

ALPB_O XE 2.566313

XFAC_O XE 1.623526

ALPB_F XE 2.837749

XFAC_F XE 2.086480

ALPB_NE XE 1.330202

XFAC_NE XE 0.293862

ALPB_NA XE 1.291138

XFAC_NA XE 5.076100

ALPB_MG XE 2.756089

XFAC_MG XE 9.774960

ALPB_AL XE 2.420691

XFAC_AL XE 7.358944

ALPB_SI XE 2.796986

XFAC_SI XE 16.526889

ALPB_CL XE 1.389615

XFAC_CL XE 0.593028

ALPB_AR XE 0.591520

XFAC_AR XE 0.049266

ALPB_K XE 0.886811

XFAC_K XE 1.526138

ALPB_CA XE 1.698890

XFAC_CA XE 2.050654

ALPB_BR XE 1.400900

XFAC_BR XE 0.711370

ALPB_KR XE 0.551561

XFAC_KR XE 0.049793

ALPB_RB XE 1.345397

XFAC_RB XE 1.856289

ALPB_I XE 1.187975

XFAC_I XE 0.555791

ALPB_XE XE 1.912510

XFAC_XE XE 9.565337

USS CS -3.996308

UPP CS -2.569885

BETAS CS -11.167340

BETAP CS 9.691485

ZS CS 1.776064

ZP CS 6.025310

GSS CS 18.164131

GSP CS 6.920824

GPP CS 16.792426

GP2 CS 8.175881

HSP CS 4.590034

ALPB_H CS 1.719572

XFAC_H CS 2.711386

ALPB_B CS 3.000034

XFAC_B CS 10.289233

ALPB_C CS 2.251416

XFAC_C CS 17.858749

ALPB_N CS 2.465681

XFAC_N CS 28.270100

ALPB_O CS 1.517551

XFAC_O CS 0.871027

ALPB_F CS 1.636155

XFAC_F CS 0.551707

ALPB_P CS 0.917812

XFAC_P CS 0.499881

ALPB_S CS 1.348833

XFAC_S CS 1.767711

ALPB_CL CS 1.241351

XFAC_CL CS 0.942491

ALPB_K CS 1.722882

XFAC_K CS 1.188658

ALPB_V CS 2.002665

XFAC_V CS 11.159719

ALPB_BR CS 1.949820

XFAC_BR CS 13.999636

ALPB_Y CS 0.929803

XFAC_Y CS 1.057035

ALPB_IN CS 0.852581

XFAC_IN CS 4.457697

ALPB_I CS 1.239277

XFAC_I CS 3.226708

ALPB_CS CS 1.267283

XFAC_CS CS 29.382256

USS BA -11.571532

UPP BA -9.917993

BETAS BA -10.914737

BETAP BA 9.727920

ZS BA 1.750490

ZP BA 1.968788

GSS BA 7.843618

GSP BA 19.900648

GPP BA 20.004643

GP2 BA 19.020523

HSP BA 0.979914

ALPB_H BA 3.120384

XFAC_H BA 27.058819

ALPB_C BA 1.318794

XFAC_C BA 0.549254

ALPB_N BA 2.188957

XFAC_N BA 4.679835

ALPB_O BA 2.337452

XFAC_O BA 4.174798

ALPB_F BA 2.539909

XFAC_F BA 3.008132

ALPB_MG BA 1.432600

XFAC_MG BA 10.342497

ALPB_AL BA 2.891358

XFAC_AL BA 15.460538

ALPB_SI BA 0.996995

XFAC_SI BA 0.887820

ALPB_P BA 1.646819

XFAC_P BA 8.719637

ALPB_S BA 1.637082

XFAC_S BA 1.742576

ALPB_CL BA 1.987384

XFAC_CL BA 2.636334

ALPB_CA BA 1.342035

XFAC_CA BA 2.833561

ALPB_TI BA 1.702345

XFAC_TI BA 4.943061

ALPB_CU BA 1.699850

XFAC_CU BA 1.896329

ALPB_BR BA 1.806723

XFAC_BR BA 2.830984

ALPB_SB BA 1.329425

XFAC_SB BA 12.262981

ALPB_I BA 1.370665

XFAC_I BA 2.112756

ALPB_BA BA 1.860576

XFAC_BA BA 57.199345

USS LA -15.586927

UPP LA 58.477136

UDD LA -19.818759

BETAS LA -18.460416

BETAP LA -19.708547

BETAD LA 0.849478

ZS LA 3.398968

ZP LA 1.811983

ZD LA 1.894574

ZSN LA 1.187188

ZPN LA 2.542482

ZDN LA 2.306744

GSS LA 4.516349

GSP LA 5.344112

GPP LA 10.610725

GP2 LA 9.202959

HSP LA 0.286106

POC LA 1.846287

F0SD LA 7.808849

G2SD LA 5.958952

ALPB_H LA 1.073406

XFAC_H LA 0.399521

ALPB_C LA 2.129683

XFAC_C LA 4.650201

ALPB_N LA 2.329214

XFAC_N LA 2.192625

ALPB_O LA 1.940554

XFAC_O LA 1.648001

ALPB_F LA 2.228378

XFAC_F LA 1.892928

ALPB_AL LA 1.003510

XFAC_AL LA 0.500540

ALPB_SI LA 2.872867

XFAC_SI LA 1.218295

ALPB_P LA 1.991054

XFAC_P LA 18.284518

ALPB_S LA 1.158196

XFAC_S LA 0.486832

ALPB_CL LA 1.835651

XFAC_CL LA 1.631876

ALPB_BR LA 1.253581

XFAC_BR LA 0.731795

ALPB_I LA 1.612519

XFAC_I LA 3.278712

ALPB_LA LA 2.066209

XFAC_LA LA 29.272376

ALPB_H GD 0.390870

XFAC_H GD 0.135810

ALPB_C GD 0.446870

XFAC_C GD 0.053040

ALPB_N GD 1.159410

XFAC_N GD 0.205050

ALPB_O GD 0.862040

XFAC_O GD 0.175800

ALPB_F GD 1.497980

XFAC_F GD 0.334630

ALPB_AL GD 1.003510

XFAC_AL GD 0.500540

ALPB_SI GD 2.112525

XFAC_SI GD 3.203995

ALPB_P GD 0.954450

XFAC_P GD 0.541660

ALPB_S GD 2.003930

XFAC_S GD 2.655400

ALPB_CL GD 0.806810

XFAC_CL GD 0.089970

ALPB_BR GD 0.715810

XFAC_BR GD 0.240740

ALPB_I GD 0.585360

XFAC_I GD 0.278240

ALPB_GD GD 3.348180

XFAC_GD GD 2.670400

USS LU -21.914035

UPP LU 54.132176

UDD LU -24.661582

BETAS LU -26.143720

BETAP LU -9.506888

BETAD LU 3.472080

ZS LU 2.327039

ZP LU 6.000335

ZD LU 1.208414

ZSN LU 0.449170

ZPN LU 2.469444

ZDN LU 2.216418

GSS LU 1.708751

GSP LU 2.037073

GPP LU 10.305910

GP2 LU 8.938585

HSP LU 0.000293

POC LU 5.824175

F0SD LU 9.700135

G2SD LU 6.013887

ALPB_H LU 2.089118

XFAC_H LU 7.421490

ALPB_C LU 2.386830

XFAC_C LU 6.432873

ALPB_N LU 1.647895

XFAC_N LU 0.783123

ALPB_O LU 1.889190

XFAC_O LU 0.868896

ALPB_F LU 1.888274

XFAC_F LU 1.730185

ALPB_P LU 1.345992

XFAC_P LU 8.048165

ALPB_CL LU 2.558367

XFAC_CL LU 8.330639

ALPB_BR LU 1.381701

XFAC_BR LU 0.992835

ALPB_I LU 1.436788

XFAC_I LU 4.313665

ALPB_LU LU 1.403653

XFAC_LU LU 21.889048

USS HF -25.690382

UPP HF -9.479410

UDD HF -39.077741

BETAS HF -4.866355

BETAP HF -21.264221

BETAD HF -12.878794

ZS HF 2.854938

ZP HF 3.079458

ZD HF 2.067146

ZSN HF 3.099683

ZPN HF 3.333027

ZDN HF 3.025020

GSS HF 11.791941

GSP HF 12.198754

GPP HF 13.909964

GP2 HF 12.064475

HSP HF 3.057466

F0SD HF 4.020384

G2SD HF 4.323408

ALPB_H HF 2.088799

XFAC_H HF 3.833288

ALPB_B HF 1.617370

XFAC_B HF 0.588837

ALPB_C HF 2.294622

XFAC_C HF 4.159075

ALPB_N HF 2.521801

XFAC_N HF 5.468404

ALPB_O HF 2.446232

XFAC_O HF 2.857484

ALPB_F HF 2.979096

XFAC_F HF 4.736067

ALPB_NA HF 1.840619

XFAC_NA HF 8.832085

ALPB_MG HF 1.911350

XFAC_MG HF 4.330250

ALPB_AL HF 0.949150

XFAC_AL HF 0.622520

ALPB_SI HF 2.189300

XFAC_SI HF 3.382300

ALPB_P HF 2.099591

XFAC_P HF 5.936976

ALPB_S HF 2.327110

XFAC_S HF 1.666760

ALPB_CL HF 1.953166

XFAC_CL HF 1.685929

ALPB_CA HF 2.054500

XFAC_CA HF 4.319510

ALPB_AS HF 1.799500

XFAC_AS HF 1.280820

ALPB_BR HF 2.237896

XFAC_BR HF 6.312154

ALPB_I HF 2.354639

XFAC_I HF 18.443532

ALPB_BA HF 2.264830

XFAC_BA HF 9.022520

ALPB_HF HF 2.216588

XFAC_HF HF 29.394192

USS TA -34.075891

UPP TA -5.504664

UDD TA -35.650460

BETAS TA -15.943219

BETAP TA 8.985389

BETAD TA -11.508162

ZS TA 4.116264

ZP TA 3.380936

ZD TA 1.755408

ZSN TA 1.011432

ZPN TA 2.139168

ZDN TA 1.685479

GSS TA 3.847731

GSP TA 4.550506

GPP TA 8.927545

GP2 TA 7.743093

HSP TA 0.256277

F0SD TA 7.257766

G2SD TA 1.619809

ALPB_H TA 1.786631

XFAC_H TA 1.893110

ALPB_C TA 1.450720

XFAC_C TA 0.581370

ALPB_N TA 2.013737

XFAC_N TA 1.152896

ALPB_O TA 2.494885

XFAC_O TA 2.316225

ALPB_F TA 2.732769

XFAC_F TA 2.163084

ALPB_NA TA 2.551120

XFAC_NA TA 8.276130

ALPB_P TA 2.513800

XFAC_P TA 6.261880

ALPB_S TA 2.091335

XFAC_S TA 3.201126

ALPB_CL TA 2.003584

XFAC_CL TA 1.661719

ALPB_K TA 4.521470

XFAC_K TA 2.026700

ALPB_BR TA 1.962327

XFAC_BR TA 3.310460

ALPB_I TA 1.500797

XFAC_I TA 1.995370

ALPB_TA TA 0.982767

XFAC_TA TA 0.831956

USS W -52.048404

UPP W -39.590059

UDD W -53.556920

BETAS W -63.148771

BETAP W -2.737119

BETAD W 1.132748

ZS W 3.881177

ZP W 2.044717

ZD W 1.928901

ZSN W 3.461491

ZPN W 1.904387

ZDN W 2.180340

GSS W 13.168346

GSP W 8.485482

GPP W 7.947717

GP2 W 6.893262

HSP W 0.826801

F0SD W 10.002668

G2SD W 3.417555

ALPB_H W 2.665390

XFAC_H W 5.441909

ALPB_C W 2.600118

XFAC_C W 4.729842

ALPB_N W 2.505903

XFAC_N W 3.877751

ALPB_O W 2.343168

XFAC_O W 1.878859

ALPB_F W 2.411756

XFAC_F W 1.368205

ALPB_NA W 1.090156

XFAC_NA W 0.686226

ALPB_MG W 1.434249

XFAC_MG W 1.904971

ALPB_P W 1.715627

XFAC_P W 4.472129

ALPB_S W 2.045564

XFAC_S W 2.401567

ALPB_CL W 1.907817

XFAC_CL W 1.349560

ALPB_K W 1.521243

XFAC_K W 2.096182

ALPB_CA W 1.870733

XFAC_CA W 8.590544

ALPB_FE W 1.787925

XFAC_FE W 1.977394

ALPB_NI W 1.775099

XFAC_NI W 1.430746

ALPB_ZN W 1.928464

XFAC_ZN W 5.376323

ALPB_BR W 2.143627

XFAC_BR W 3.993357

ALPB_RB W 0.900113

XFAC_RB W 4.075269

ALPB_ZR W 2.023641

XFAC_ZR W 19.994079

ALPB_I W 1.997307

XFAC_I W 5.825642

ALPB_CS W 0.899625

XFAC_CS W 4.061044

ALPB_BA W 1.566159

XFAC_BA W 1.861828

ALPB_W W 2.141401

XFAC_W W 13.807246

USS RE -41.679545

UPP RE 43.429894

UDD RE -54.761512

BETAS RE 8.467231

BETAP RE -6.468335

BETAD RE -11.136390

ZS RE 2.452162

ZP RE 1.583194

ZD RE 2.414839

ZSN RE 2.433415

ZPN RE 0.838026

ZDN RE 1.921708

GSS RE 9.257297

GSP RE 3.796290

GPP RE 3.497395

GP2 RE 3.033382

HSP RE 0.046330

F0SD RE 5.229236

G2SD RE 1.821985

ALPB_H RE 1.748317

XFAC_H RE 0.497281

ALPB_C RE 2.109510

XFAC_C RE 0.646616

ALPB_N RE 2.474230

XFAC_N RE 1.438570

ALPB_O RE 2.403640

XFAC_O RE 1.075351

ALPB_F RE 2.790322

XFAC_F RE 1.384171

ALPB_SI RE 2.775930

XFAC_SI RE 0.849450

ALPB_P RE 1.316878

XFAC_P RE 0.761808

ALPB_S RE 2.637193

XFAC_S RE 3.055234

ALPB_CL RE 2.857608

XFAC_CL RE 3.265852

ALPB_GE RE 2.852340

XFAC_GE RE 2.151580

ALPB_SE RE 2.523170

XFAC_SE RE 2.202140

ALPB_BR RE 2.195052

XFAC_BR RE 1.575571

ALPB_SB RE 2.204360

XFAC_SB RE 2.275780

ALPB_I RE 2.239594

XFAC_I RE 3.240592

ALPB_RE RE 2.195649

XFAC_RE RE 1.776660

USS OS -65.963764

UPP OS 37.736568

UDD OS -89.718816

BETAS OS -43.486712

BETAP OS -25.607006

BETAD OS -1.430819

ZS OS 3.094808

ZP OS 2.845232

ZD OS 1.986395

ZSN OS 2.613281

ZPN OS 2.062936

ZDN OS 2.944917

GSS OS 9.941551

GSP OS 8.617668

GPP OS 8.609401

GP2 OS 7.467158

HSP OS 1.886033

F0SD OS 8.758980

G2SD OS 4.717871

ALPB_H OS 2.399448

XFAC_H OS 3.609773

ALPB_C OS 1.938959

XFAC_C OS 0.616916

ALPB_N OS 2.139750

XFAC_N OS 0.730399

ALPB_O OS 2.539022

XFAC_O OS 1.230187

ALPB_F OS 2.210417

XFAC_F OS 0.562952

ALPB_NA OS 2.550740

XFAC_NA OS 8.275750

ALPB_P OS 2.060122

XFAC_P OS 4.267629

ALPB_S OS 2.809500

XFAC_S OS 4.186050

ALPB_CL OS 2.080978

XFAC_CL OS 1.177666

ALPB_K OS 1.351484

XFAC_K OS 0.875486

ALPB_BR OS 2.225810

XFAC_BR OS 2.709104

ALPB_I OS 2.189487

XFAC_I OS 4.869377

ALPB_OS OS 1.661052

XFAC_OS OS 0.928334

USS IR -40.856798

UPP IR -2.270208

UDD IR -68.020682

BETAS IR -11.770307

BETAP IR -13.487742

BETAD IR -5.642629

ZS IR 1.924564

ZP IR 3.510744

ZD IR 2.437796

ZSN IR 2.108777

ZPN IR 0.618406

ZDN IR 1.826929

GSS IR 8.022296

GSP IR 2.803574

GPP IR 2.580839

GP2 IR 2.238429

HSP IR 0.013100

F0SD IR 3.726074

G2SD IR 2.747207

ALPB_H IR 1.634365

XFAC_H IR 0.406470

ALPB_C IR 1.604977

XFAC_C IR 0.185955

ALPB_N IR 2.997358

XFAC_N IR 1.790021

ALPB_O IR 3.116069

XFAC_O IR 2.303902

ALPB_F IR 2.612609

XFAC_F IR 0.714245

ALPB_NA IR 2.550820

XFAC_NA IR 8.275830

ALPB_P IR 2.714060

XFAC_P IR 6.284670

ALPB_S IR 3.009199

XFAC_S IR 2.680449

ALPB_CL IR 2.575683

XFAC_CL IR 0.858848

ALPB_K IR 4.521170

XFAC_K IR 2.026400

ALPB_BR IR 2.058351

XFAC_BR IR 0.804901

ALPB_I IR 2.031222

XFAC_I IR 1.787121

ALPB_CS IR 1.559526

XFAC_CS IR 1.027369

ALPB_IR IR 1.465795

XFAC_IR IR 0.190914

USS PT -55.878758

UPP PT 52.660706

UDD PT -92.789895

BETAS PT -10.270452

BETAP PT 10.016048

BETAD PT -7.705919

ZS PT 2.922551

ZP PT 0.725689

ZD PT 2.158085

ZSN PT 3.083320

ZPN PT 19.427280

ZDN PT 2.233704

GSS PT 11.729692

GSP PT 13.983535

GPP PT 81.077279

GP2 PT 70.320441

HSP PT 0.000643

F0SD PT 4.725137

G2SD PT 3.459127

ALPB_H PT 3.062604

XFAC_H PT 2.051954

ALPB_C PT 2.296772

XFAC_C PT 0.370388

ALPB_N PT 2.347134

XFAC_N PT 0.447775

ALPB_O PT 2.680367

XFAC_O PT 0.827827

ALPB_F PT 3.157007

XFAC_F PT 1.031240

ALPB_AL PT 1.572360

XFAC_AL PT 1.056930

ALPB_SI PT 0.999990

XFAC_SI PT 0.099990

ALPB_P PT 1.307810

XFAC_P PT 0.485582

ALPB_S PT 2.919597

XFAC_S PT 2.008326

ALPB_CL PT 3.034813

XFAC_CL PT 1.610994

ALPB_K PT 1.495407

XFAC_K PT 2.058817

ALPB_BR PT 2.596546

XFAC_BR PT 1.409311

ALPB_AG PT 1.387422

XFAC_AG PT 5.456551

ALPB_I PT 2.228284

XFAC_I PT 1.174520

ALPB_PT PT 3.276872

XFAC_PT PT 8.178033

USS AU -94.841695

UPP AU -61.195249

UDD AU -114.242383

BETAS AU -13.460355

BETAP AU -24.921790

BETAD AU -63.835796

ZS AU 1.904923

ZP AU 2.408005

ZD AU 4.377691

ZSN AU 2.228930

ZPN AU 4.555019

ZDN AU 2.406645

GSS AU 8.479387

GSP AU 10.011584

GPP AU 19.009792

GP2 AU 16.487689

HSP AU 0.645273

F0SD AU 9.054233

G2SD AU 5.690708

ALPB_H AU 2.006469

XFAC_H AU 0.748516

ALPB_C AU 2.119485

XFAC_C AU 0.603200

ALPB_N AU 2.395362

XFAC_N AU 0.620935

ALPB_O AU 2.323131

XFAC_O AU 0.355344

ALPB_F AU 3.153884

XFAC_F AU 0.880186

ALPB_AL AU 1.572570

XFAC_AL AU 1.057140

ALPB_P AU 1.360881

XFAC_P AU 0.477023

ALPB_S AU 1.908644

XFAC_S AU 0.423252

ALPB_CL AU 2.495913

XFAC_CL AU 1.153029

ALPB_K AU 1.098797

XFAC_K AU 0.777289

ALPB_SE AU 1.840962

XFAC_SE AU 1.396436

ALPB_BR AU 1.633736

XFAC_BR AU 0.259683

ALPB_PD AU 1.311827

XFAC_PD AU 0.663311

ALPB_I AU 2.017017

XFAC_I AU 1.635086

ALPB_AU AU 1.539843

XFAC_AU AU 0.352184

USS HG -18.205464

UPP HG -15.280873

BETAS HG -9.919880

BETAP HG 2.134696

ZS HG 2.575831

ZP HG 1.955505

GSS HG 7.687301

GSP HG 8.355361

GPP HG 5.135379

GP2 HG 9.529761

HSP HG 0.954727

ALPB_H HG 1.953060

XFAC_H HG 3.306359

ALPB_C HG 1.702331

XFAC_C HG 0.911944

ALPB_N HG 1.715039

XFAC_N HG 1.016140

ALPB_O HG 2.151298

XFAC_O HG 2.032727

ALPB_F HG 1.836494

XFAC_F HG 0.631905

ALPB_NA HG 1.459803

XFAC_NA HG 2.437893

ALPB_SI HG 2.770860

XFAC_SI HG 3.680740

ALPB_P HG 0.891179

XFAC_P HG 1.351633

ALPB_S HG 1.900145

XFAC_S HG 1.772158

ALPB_CL HG 1.838378

XFAC_CL HG 1.142381

ALPB_TI HG 3.414630

XFAC_TI HG 2.957200

ALPB_SE HG 1.607270

XFAC_SE HG 1.015811

ALPB_BR HG 1.705395

XFAC_BR HG 1.997568

ALPB_TE HG 1.536568

XFAC_TE HG 4.486299

ALPB_I HG 1.476731

XFAC_I HG 2.489683

ALPB_HG HG 2.288223

XFAC_HG HG 29.334203

USS TL -31.112183

UPP TL -18.547083

UDD TL 10.294626

BETAS TL -2.456566

BETAP TL -4.949902

BETAD TL 0.079835

ZS TL 1.903342

ZP TL 2.838647

ZD TL 5.015677

GSS TL 11.438997

GSP TL 6.598450

GPP TL 6.054580

GP2 TL 5.507624

HSP TL 0.894757

ALPB_H TL 2.098110

XFAC_H TL 2.104104

ALPB_B TL 1.558857

XFAC_B TL 8.505888

ALPB_C TL 2.721075

XFAC_C TL 5.320930

ALPB_N TL 1.975560

XFAC_N TL 1.168533

ALPB_O TL 3.327926

XFAC_O TL 14.162059

ALPB_F TL 3.188782

XFAC_F TL 5.857103

ALPB_AL TL 1.458567

XFAC_AL TL 7.820754

ALPB_S TL 2.648560

XFAC_S TL 7.355971

ALPB_CL TL 3.127130

XFAC_CL TL 15.106797

ALPB_BR TL 2.596395

XFAC_BR TL 9.792163

ALPB_RB TL 1.512698

XFAC_RB TL 19.748653

ALPB_I TL 2.616072

XFAC_I TL 30.000873

ALPB_TL TL 2.597707

XFAC_TL TL 32.531404

USS PB -39.446347

UPP PB -29.348301

UDD PB -72.584748

BETAS PB -64.174888

BETAP PB -4.631384

BETAD PB -5.319005

ZS PB 4.706006

ZP PB 2.591455

GSS PB 8.368048

GSP PB 8.606930

GPP PB 6.431147

GP2 PB 6.550076

HSP PB 0.984819

ALPB_H PB 2.827636

XFAC_H PB 11.387111

ALPB_LI PB 0.947660

XFAC_LI PB 1.191773

ALPB_B PB 1.718658

XFAC_B PB 2.379671

ALPB_C PB 2.608618

XFAC_C PB 6.379969

ALPB_N PB 1.830414

XFAC_N PB 1.025862

ALPB_O PB 2.980453

XFAC_O PB 4.952015

ALPB_F PB 3.676637

XFAC_F PB 9.548494

ALPB_SI PB 1.284024

XFAC_SI PB 1.429987

ALPB_P PB 1.955648

XFAC_P PB 13.812691

ALPB_S PB 2.362854

XFAC_S PB 5.436090

ALPB_CL PB 1.499678

XFAC_CL PB 0.736101

ALPB_CA PB 1.691921

XFAC_CA PB 6.709141

ALPB_V PB 1.501708

XFAC_V PB 3.819119

ALPB_CR PB 1.305185

XFAC_CR PB 0.974428

ALPB_ZN PB 1.434109

XFAC_ZN PB 2.865002

ALPB_AS PB 1.792215

XFAC_AS PB 4.943875

ALPB_SE PB 2.893161

XFAC_SE PB 29.986812

ALPB_BR PB 2.364003

XFAC_BR PB 6.777519

ALPB_NB PB 1.500000

XFAC_NB PB 1.000000

ALPB_MO PB 1.759074

XFAC_MO PB 5.265939

ALPB_TE PB 3.242448

XFAC_TE PB 176.768383

ALPB_I PB 2.179090

XFAC_I PB 8.112077

ALPB_W PB 1.517042

XFAC_W PB 1.512242

ALPB_PB PB 2.529682

XFAC_PB PB 38.479040

USS BI -36.561343

UPP BI -30.823167

UDD BI -19.667431

BETAS BI -63.673960

BETAP BI -6.931981

BETAD BI -8.868066

ZS BI 5.465413

ZP BI 2.037481

ZD BI 2.855400

ZSN BI 4.275828

ZPN BI 3.018252

ZDN BI 4.889868

GSS BI 3.438678

GSP BI 3.987429

GPP BI 8.221978

GP2 BI 8.183927

HSP BI 1.610989

ALPB_H BI 1.727556

XFAC_H BI 1.225129

ALPB_LI BI 1.221685

XFAC_LI BI 2.187383

ALPB_C BI 1.970985

XFAC_C BI 1.397988

ALPB_N BI 1.976984

XFAC_N BI 1.315182

ALPB_O BI 2.337898

XFAC_O BI 1.621567

ALPB_F BI 2.029420

XFAC_F BI 0.490733

ALPB_NA BI 1.532800

XFAC_NA BI 2.410886

ALPB_S BI 1.866193

XFAC_S BI 1.624988

ALPB_CL BI 1.405944

XFAC_CL BI 0.496440

ALPB_K BI 1.417970

XFAC_K BI 2.123183

ALPB_SE BI 1.609528

XFAC_SE BI 1.139985

ALPB_BR BI 1.750597

XFAC_BR BI 1.792203

ALPB_RB BI 1.528441

XFAC_RB BI 2.435372

ALPB_I BI 1.592333

XFAC_I BI 2.364966

ALPB_CS BI 1.567880

XFAC_CS BI 2.314878

ALPB_BI BI 1.756620

XFAC_BI BI 7.710187

## Parameters used in PM7-TS

Parameter Type Element Parameter

USS H -11.261775

BETAS H -8.152970

ZS H 1.216290

GSS H 14.297348

FN11 H 0.313713

FN21 H 1.344998

FN31 H 0.944327

ALPB_H H 5.434919

XFAC_H H 1.449515

USS HE -31.770969

UPP HE -5.856382

BETAS HE -58.903774

BETAP HE -37.039974

ZS HE 3.313204

ZP HE 3.657133

GSS HE 9.445299

GSP HE 11.201419

GPP HE 9.214548

GP2 HE 13.046115

HSP HE 0.299954

ALPB_H HE 2.989881

XFAC_H HE 2.371199

ALPB_HE HE 3.783559

XFAC_HE HE 3.450900

USS LI -4.804124

UPP LI -2.450842

BETAS LI -2.082310

BETAP LI -27.085547

ZS LI 0.804974

ZP LI 6.027530

GSS LI 9.175811

GSP LI 16.614419

GPP LI 14.193195

GP2 LI 11.289123

HSP LI 3.533317

ALPB_H LI 1.265105

XFAC_H LI 0.488118

ALPB_HE LI 2.982569

XFAC_HE LI 8.316732

ALPB_LI LI 3.213216

XFAC_LI LI 16.832394

USS BE -17.427477

UPP BE -14.843910

BETAS BE -3.965129

BETAP BE -8.623194

ZS BE 1.036199

ZP BE 1.764629

GSS BE 9.590009

GSP BE 9.878338

GPP BE 8.195145

GP2 BE 10.136549

HSP BE 0.966381

ALPB_H BE 2.854611

XFAC_H BE 3.447327

ALPB_HE BE 3.367214

XFAC_HE BE 12.563185

ALPB_LI BE 2.432991

XFAC_LI BE 11.306301

ALPB_BE BE 2.042783

XFAC_BE BE 1.680607

USS B -26.613990

UPP B -23.468278

BETAS B -7.509528

BETAP B -3.775165

ZS B 1.560481

ZP B 1.449712

GSS B 6.418667

GSP B 10.191243

GPP B 5.675076

GP2 B 6.689156

HSP B 0.942923

ALPB_H B 2.314226

XFAC_H B 1.135897

ALPB_HE B 3.163140

XFAC_HE B 1.974170

ALPB_LI B 3.000118

XFAC_LI B 5.549420

ALPB_BE B 1.991820

XFAC_BE B 1.213171

ALPB_B B 2.181999

XFAC_B B 0.964011

USS C -50.960019

UPP C -40.435130

BETAS C -14.717624

BETAP C -7.349032

ZS C 1.595550

ZP C 1.758854

GSS C 12.596831

GSP C 12.098962

GPP C 9.682694

GP2 C 9.302980

HSP C 0.000000

FN11 C 0.038893

FN21 C 8.422140

FN31 C 1.609247

ALPB_H C 1.185200

XFAC_H C 0.229917

ALPB_HE C 3.042705

XFAC_HE C 3.213971

ALPB_LI C 3.061752

XFAC_LI C 7.351225

ALPB_BE C 2.798358

XFAC_BE C 2.803943

ALPB_B C 2.650092

XFAC_B C 1.456708

ALPB_C C 2.550719

XFAC_C C 0.886228

USS N -61.478292

UPP N -49.372270

BETAS N -21.619844

BETAP N -16.781578

ZS N 2.748036

ZP N 2.221220

GSS N 10.191875

GSP N 10.113670

GPP N 11.599644

GP2 N 9.465723

HSP N 3.302388

FN11 N 0.055353

FN21 N 8.986627

FN31 N 1.704364

ALPB_H N 2.126511

XFAC_H N 0.452656

ALPB_HE N 2.814339

XFAC_HE N 1.077861

ALPB_LI N 2.205062

XFAC_LI N 1.070477

ALPB_BE N 2.391056

XFAC_BE N 1.588438

ALPB_B N 2.264882

XFAC_B N 0.794623

ALPB_C N 3.026848

XFAC_C N 1.247224

ALPB_N N 2.500120

XFAC_N N 0.585763

USS O -95.875282

UPP O -71.249369

BETAS O -67.540697

BETAP O -19.352174

ZS O 5.451637

ZP O 2.961501

GSS O 12.060136

GSP O 15.854464

GPP O 14.431363

GP2 O 10.604669

HSP O 5.901024

FN11 O -0.400950

FN21 O 1.354623

FN31 O 0.709774

ALPB_H O 1.742437

XFAC_H O 0.167623

ALPB_HE O 3.595772

XFAC_HE O 6.688705

ALPB_LI O 2.090902

XFAC_LI O 0.499065

ALPB_BE O 3.081366

XFAC_BE O 2.735812

ALPB_B O 2.694696

XFAC_B O 1.106827

ALPB_C O 3.004017

XFAC_C O 1.009223

ALPB_N O 2.585050

XFAC_N O 0.492684

ALPB_O O 2.647743

XFAC_O O 0.722294

USS F -137.257117

UPP F -98.208856

BETAS F -70.000231

BETAP F -29.886198

ZS F 5.784543

ZP F 2.965266

GSS F 15.546585

GSP F 18.508384

GPP F 10.636570

GP2 F 11.861586

HSP F 2.207295

ALPB_H F 3.546514

XFAC_H F 0.880187

ALPB_HE F 2.856543

XFAC_HE F 0.745107

ALPB_LI F 3.036354

XFAC_LI F 0.756703

ALPB_BE F 3.328175

XFAC_BE F 2.273878

ALPB_B F 2.772369

XFAC_B F 0.948178

ALPB_C F 3.553237

XFAC_C F 1.595498

ALPB_N F 3.207271

XFAC_N F 0.894011

ALPB_O F 4.086118

XFAC_O F 0.499992

ALPB_F F 4.628249

XFAC_F F 4.152435

USS NE -2.978729

UPP NE -85.441118

BETAS NE -69.793475

BETAP NE -33.261962

ZS NE 6.000148

ZP NE 3.834528

GSS NE 19.999574

GSP NE 16.896951

GPP NE 8.963560

GP2 NE 16.027799

HSP NE 1.779280

ALPB_H NE 5.999680

XFAC_H NE 5.535021

ALPB_HE NE 3.677758

XFAC_HE NE 1.960924

ALPB_LI NE 2.242969

XFAC_LI NE 0.642933

ALPB_BE NE 0.832530

XFAC_BE NE 0.140208

ALPB_B NE 2.756190

XFAC_B NE 2.764140

ALPB_C NE 3.441188

XFAC_C NE 5.468780

ALPB_N NE 4.426370

XFAC_N NE 29.999609

ALPB_O NE 2.906840

XFAC_O NE 0.753518

ALPB_F NE 3.675611

XFAC_F NE 2.706754

ALPB_NE NE 5.180440

XFAC_NE NE 0.500000

USS NA -5.815476

UPP NA -3.731003

BETAS NA 8.483380

BETAP NA -5.735680

ZS NA 1.666701

ZP NA 1.397571

GSS NA 20.011368

GSP NA 20.020053

GPP NA 12.820792

GP2 NA 19.015416

HSP NA 5.020547

ALPB_H NA 1.619287

XFAC_H NA 1.966963

ALPB_HE NA 2.171840

XFAC_HE NA 4.590369

ALPB_LI NA 0.898897

XFAC_LI NA 0.655446

ALPB_BE NA 1.255480

XFAC_BE NA 3.121620

ALPB_B NA 2.476698

XFAC_B NA 8.164739

ALPB_C NA 2.394648

XFAC_C NA 6.318544

ALPB_N NA 2.482865

XFAC_N NA 5.755473

ALPB_O NA 2.699675

XFAC_O NA 8.556302

ALPB_F NA 3.036873

XFAC_F NA 9.250233

ALPB_NE NA 1.469568

XFAC_NE NA 0.697745

ALPB_NA NA 1.994455

XFAC_NA NA 9.335783

USS MG -14.858681

UPP MG -12.451227

BETAS MG -12.576970

BETAP MG -0.702739

ZS MG 1.170297

ZP MG 1.840439

GSS MG 7.480635

GSP MG 9.602125

GPP MG 8.869755

GP2 MG 6.241718

HSP MG 0.992746

ALPB_H MG 2.423259

XFAC_H MG 6.170068

ALPB_HE MG 2.289485

XFAC_HE MG 3.779366

ALPB_LI MG 1.374791

XFAC_LI MG 2.510632

ALPB_BE MG 1.593445

XFAC_BE MG 2.960809

ALPB_B MG 2.466919

XFAC_B MG 6.072802

ALPB_C MG 2.321772

XFAC_C MG 3.390341

ALPB_N MG 2.025732

XFAC_N MG 2.115961

ALPB_O MG 2.730174

XFAC_O MG 2.888295

ALPB_F MG 3.378507

XFAC_F MG 5.439497

ALPB_NE MG 0.922342

XFAC_NE MG 0.452636

ALPB_NA MG 1.682212

XFAC_NA MG 8.429332

ALPB_MG MG 2.055257

XFAC_MG MG 20.557591

USS AL -32.518856

UPP AL -24.873064

UDD AL -31.418925

BETAS AL 6.109627

BETAP AL -2.986557

BETAD AL -28.937998

ZS AL 1.232599

ZP AL 1.219336

ZD AL 1.617502

ZSN AL 2.346908

ZPN AL 1.529050

ZDN AL 3.682742

ALP AL 5.341685

GSS AL 10.347944

GSP AL 9.180517

GPP AL 7.181623

GP2 AL 5.626787

HSP AL 0.920418

ALPB_H AL 1.610842

XFAC_H AL 1.183718

ALPB_HE AL 2.255830

XFAC_HE AL 2.701400

ALPB_LI AL 1.248327

XFAC_LI AL 0.929842

ALPB_BE AL 1.916502

XFAC_BE AL 4.229824

ALPB_B AL 1.990198

XFAC_B AL 2.676137

ALPB_C AL 2.058949

XFAC_C AL 3.161882

ALPB_N AL 1.477524

XFAC_N AL 0.883919

ALPB_O AL 2.054038

XFAC_O AL 1.619036

ALPB_F AL 2.253927

XFAC_F AL 1.368035

ALPB_NE AL 2.528574

XFAC_NE AL 1.702157

ALPB_NA AL 1.141388

XFAC_NA AL 1.128163

ALPB_MG AL 1.455074

XFAC_MG AL 1.821180

ALPB_AL AL 1.224852

XFAC_AL AL 1.669052

USS SI -41.586357

UPP SI -36.694055

UDD SI -16.775635

BETAS SI -10.755885

BETAP SI -3.922152

BETAD SI -4.736877

ZS SI 1.433994

ZP SI 1.671776

ZD SI 1.221915

ZSN SI 2.002570

ZPN SI 0.818377

ZDN SI 2.591238

GSS SI 8.159128

GSP SI 11.213512

GPP SI 8.521933

GP2 SI 8.493112

HSP SI 0.959479

ALPB_H SI 1.542308

XFAC_H SI 0.688945

ALPB_HE SI 2.028628

XFAC_HE SI 1.976149

ALPB_LI SI 1.911808

XFAC_LI SI 2.989391

ALPB_BE SI 2.162457

XFAC_BE SI 4.322374

ALPB_B SI 1.915795

XFAC_B SI 1.162577

ALPB_C SI 1.673306

XFAC_C SI 0.501779

ALPB_N SI 1.854197

XFAC_N SI 0.671576

ALPB_O SI 1.824047

XFAC_O SI 0.502254

ALPB_F SI 2.160762

XFAC_F SI 0.564372

ALPB_NE SI 2.655346

XFAC_NE SI 12.754805

ALPB_NA SI 1.842304

XFAC_NA SI 9.125996

ALPB_MG SI 1.157990

XFAC_MG SI 0.527802

ALPB_AL SI 1.300963

XFAC_AL SI 1.290056

ALPB_SI SI 1.109923

XFAC_SI SI 0.369696

USS P -66.851313

UPP P -30.452730

UDD P -9.386661

BETAS P -45.727463

BETAP P -12.437420

BETAD P -34.163510

ZS P 3.095543

ZP P 1.287530

ZD P 0.672700

ZSN P 4.812508

ZPN P 1.095716

ZDN P 12.117992

GSS P 4.108409

GSP P 7.832870

GPP P 6.080710

GP2 P 4.180298

HSP P 1.734680

ALPB_H P 1.467875

XFAC_H P 1.449388

ALPB_HE P 2.076667

XFAC_HE P 1.493985

ALPB_LI P 1.727121

XFAC_LI P 5.864987

ALPB_BE P 1.872176

XFAC_BE P 2.310001

ALPB_B P 1.742693

XFAC_B P 2.541187

ALPB_C P 2.122583

XFAC_C P 1.077228

ALPB_N P 1.594467

XFAC_N P 1.588417

ALPB_O P 2.143607

XFAC_O P 0.512206

ALPB_F P 2.756139

XFAC_F P 0.913167

ALPB_NE P 2.243688

XFAC_NE P 0.762937

ALPB_NA P 1.518961

XFAC_NA P 3.713750

ALPB_MG P 1.297069

XFAC_MG P 1.585367

ALPB_AL P 1.375504

XFAC_AL P 3.249399

ALPB_SI P 0.895674

XFAC_SI P 0.616954

ALPB_P P 1.358793

XFAC_P P 2.560912

USS S -51.157757

UPP S -40.352643

UDD S -48.529935

BETAS S -11.422550

BETAP S -7.191896

BETAD S -10.695329

ZS S 2.046153

ZP S 1.807678

ZD S 3.510309

ZSN S 1.131343

ZPN S 0.823803

ZDN S 2.296065

GSS S 8.728478

GSP S 6.483871

GPP S 7.357401

GP2 S 6.875448

HSP S 3.012199

ALPB_H S 2.182464

XFAC_H S 0.703252

ALPB_HE S 1.959149

XFAC_HE S 0.437618

ALPB_LI S 1.737806

XFAC_LI S 0.566769

ALPB_BE S 2.575836

XFAC_BE S 3.179465

ALPB_B S 2.363313

XFAC_B S 1.177082

ALPB_C S 2.429136

XFAC_C S 0.843145

ALPB_N S 2.653791

XFAC_N S 1.197307

ALPB_O S 2.508022

XFAC_O S 0.729340

ALPB_F S 2.533157

XFAC_F S 0.534080

ALPB_NE S 2.787058

XFAC_NE S 3.296160

ALPB_NA S 2.614090

XFAC_NA S 6.263298

ALPB_MG S 1.442313

XFAC_MG S 0.578881

ALPB_AL S 1.706655

XFAC_AL S 1.677290

ALPB_SI S 1.647931

XFAC_SI S 0.553963

ALPB_P S 1.596824

XFAC_P S 1.189185

ALPB_S S 1.985120

XFAC_S S 0.509363

USS CL -68.736411

UPP CL -57.994735

UDD CL -49.225659

BETAS CL -2.876904

BETAP CL -14.469119

BETAD CL 1.958261

ZS CL 2.324992

ZP CL 2.432096

ZD CL 2.645212

ZSN CL 2.353207

ZPN CL 2.099376

ZDN CL 5.523670

GSS CL 9.804995

GSP CL 7.172184

GPP CL 7.864837

GP2 CL 9.017278

HSP CL 5.858548

ALPB_H CL 2.448633

XFAC_H CL 0.498763

ALPB_HE CL 1.671634

XFAC_HE CL 0.500002

ALPB_LI CL 2.838217

XFAC_LI CL 2.531354

ALPB_BE CL 2.716560

XFAC_BE CL 2.638266

ALPB_B CL 2.228737

XFAC_B CL 0.742613

ALPB_C CL 2.446801

XFAC_C CL 0.499496

ALPB_N CL 2.385624

XFAC_N CL 0.659831

ALPB_O CL 2.352582

XFAC_O CL 0.340364

ALPB_F CL 2.772246

XFAC_F CL 0.637890

ALPB_NE CL 1.732740

XFAC_NE CL 0.499482

ALPB_NA CL 2.536945

XFAC_NA CL 10.364642

ALPB_MG CL 2.292455

XFAC_MG CL 2.207847

ALPB_AL CL 1.678498

XFAC_AL CL 1.079875

ALPB_SI CL 1.818389

XFAC_SI CL 0.590060

ALPB_P CL 1.297513

XFAC_P CL 0.496813

ALPB_S CL 2.167945

XFAC_S CL 0.624384

ALPB_CL CL 2.052525

XFAC_CL CL 1.120660

USS AR -7.797931

UPP AR -83.211487

BETAS AR -8.839842

BETAP AR -28.427303

ZS AR 6.000272

ZP AR 5.949170

GSS AR 17.858776

GSP AR 4.168451

GPP AR 11.852500

GP2 AR 15.669543

HSP AR 4.574549

ALPB_H AR 4.056167

XFAC_H AR 3.933445

ALPB_HE AR 2.716562

XFAC_HE AR 1.177211

ALPB_LI AR 3.001334

XFAC_LI AR 2.193788

ALPB_BE AR 3.227598

XFAC_BE AR 2.700296

ALPB_B AR 2.674207

XFAC_B AR 2.017996

ALPB_C AR 1.471309

XFAC_C AR 0.122309

ALPB_N AR 2.326805

XFAC_N AR 0.562581

ALPB_O AR 2.228209

XFAC_O AR 0.367713

ALPB_F AR 3.920658

XFAC_F AR 9.269715

ALPB_NE AR 2.963747

XFAC_NE AR 1.304697

ALPB_NA AR 2.667734

XFAC_NA AR 5.946915

ALPB_MG AR 1.996514

XFAC_MG AR 2.030224

ALPB_AL AR 2.716128

XFAC_AL AR 1.838228

ALPB_SI AR 1.935869

XFAC_SI AR 1.288907

ALPB_P AR 3.998905

XFAC_P AR 0.173766

ALPB_S AR 2.049398

XFAC_S AR 0.653769

ALPB_CL AR 2.554449

XFAC_CL AR 2.256094

ALPB_AR AR 2.306432

XFAC_AR AR 0.972699

USS K -4.888065

UPP K -3.763457

BETAS K 10.013029

BETAP K -2.882668

ZS K 5.422018

ZP K 1.471023

GSS K 19.497974

GSP K 4.674636

GPP K 4.339481

GP2 K 5.981455

HSP K 1.092988

ALPB_H K 2.304518

XFAC_H K 29.964954

ALPB_HE K 2.140614

XFAC_HE K 6.673621

ALPB_LI K 1.108062

XFAC_LI K 4.364297

ALPB_BE K 3.000365

XFAC_BE K 6.514383

ALPB_B K 2.507524

XFAC_B K 28.190857

ALPB_C K 1.769643

XFAC_C K 2.489951

ALPB_N K 1.907394

XFAC_N K 3.943077

ALPB_O K 2.151119

XFAC_O K 4.281570

ALPB_F K 3.065393

XFAC_F K 17.321092

ALPB_NE K 1.653125

XFAC_NE K 1.093188

ALPB_NA K 0.944935

XFAC_NA K 6.450008

ALPB_MG K 1.272102

XFAC_MG K 2.832505

ALPB_AL K 1.849469

XFAC_AL K 27.774025

ALPB_SI K 1.674691

XFAC_SI K 8.047633

ALPB_P K 1.415563

XFAC_P K 4.258021

ALPB_S K 2.428403

XFAC_S K 30.000181

ALPB_CL K 2.346443

XFAC_CL K 12.630753

ALPB_AR K 2.436124

XFAC_AR K 8.318024

ALPB_K K 1.492751

XFAC_K K 6.173527

USS CA -13.503503

UPP CA -10.559344

BETAS CA -11.696053

BETAP CA 4.968210

ZS CA 1.477988

ZP CA 2.220194

GSS CA 7.914200

GSP CA 6.712903

GPP CA 4.997910

GP2 CA 4.995881

HSP CA 1.170905

ALPB_H CA 1.997037

XFAC_H CA 5.125996

ALPB_HE CA 2.150217

XFAC_HE CA 5.381385

ALPB_B CA 1.700010

XFAC_B CA 1.700010

ALPB_C CA 3.376881

XFAC_C CA 45.518388

ALPB_N CA 2.335548

XFAC_N CA 3.063067

ALPB_O CA 3.347983

XFAC_O CA 8.353090

ALPB_F CA 3.871263

XFAC_F CA 14.692101

ALPB_NE CA 1.247453

XFAC_NE CA 0.493997

ALPB_NA CA 2.172223

XFAC_NA CA 10.049083

ALPB_MG CA 1.612133

XFAC_MG CA 5.062878

ALPB_AL CA 1.612565

XFAC_AL CA 4.188555

ALPB_SI CA 1.730018

XFAC_SI CA 4.282139

ALPB_P CA 1.922605

XFAC_P CA 15.033250

ALPB_S CA 1.481189

XFAC_S CA 0.561550

ALPB_CL CA 2.785624

XFAC_CL CA 8.996518

ALPB_AR CA 1.544903

XFAC_AR CA 0.699868

ALPB_K CA 1.210391

XFAC_K CA 1.755307

ALPB_CA CA 1.477787

XFAC_CA CA 5.134189

USS SC -19.383239

UPP SC -15.936628

UDD SC -20.365590

BETAS SC -16.127750

BETAP SC -4.714646

BETAD SC -8.631714

ZS SC 1.794897

ZP SC 2.174934

ZD SC 5.992860

ZSN SC 1.314009

ZPN SC 1.020629

ZDN SC 1.437857

ALP SC 0.991198

GSS SC 7.183554

GSP SC 6.188166

GPP SC 6.079855

GP2 SC 5.329586

HSP SC 1.340355

POC SC 1.070880

F0SD SC 8.096837

G2SD SC 3.531412

ALPB_H SC 2.630734

XFAC_H SC 5.354101

ALPB_C SC 2.774943

XFAC_C SC 13.452840

ALPB_N SC 2.081124

XFAC_N SC 1.980291

ALPB_O SC 2.238586

XFAC_O SC 1.567669

ALPB_F SC 3.226175

XFAC_F SC 7.919620

ALPB_AL SC 1.003550

XFAC_AL SC 0.500620

ALPB_SI SC 1.849600

XFAC_SI SC 2.767826

ALPB_P SC 1.919608

XFAC_P SC 4.663061

ALPB_S SC 1.111949

XFAC_S SC 0.498540

ALPB_CL SC 2.094163

XFAC_CL SC 2.355302

ALPB_SC SC 2.106571

XFAC_SC SC 30.002441

USS TI -26.608414

UPP TI -23.616842

UDD TI -28.876758

BETAS TI -5.411644

BETAP TI -4.838856

BETAD TI 0.774574

ZS TI 1.448579

ZP TI 1.940695

ZD TI 1.093648

ZSN TI 1.078295

ZPN TI 4.663707

ZDN TI 0.954258

GSS TI 5.894930

GSP TI 7.330203

GPP TI 27.781556

GP2 TI 24.353243

HSP TI 0.044555

F0SD TI 6.384127

G2SD TI 3.488564

ALPB_H TI 1.447725

XFAC_H TI 0.603333

ALPB_LI TI 1.514050

XFAC_LI TI 0.502488

ALPB_B TI 1.628710

XFAC_B TI 0.649360

ALPB_C TI 1.798067

XFAC_C TI 0.562296

ALPB_N TI 1.638936

XFAC_N TI 0.543706

ALPB_O TI 1.962314

XFAC_O TI 0.872204

ALPB_F TI 2.186657

XFAC_F TI 0.836131

ALPB_NA TI 1.124786

XFAC_NA TI 1.987793

ALPB_MG TI 1.900606

XFAC_MG TI 6.889073

ALPB_AL TI 1.833384

XFAC_AL TI 8.952566

ALPB_SI TI 1.373954

XFAC_SI TI 0.561089

ALPB_P TI 1.610003

XFAC_P TI 3.074680

ALPB_S TI 2.309450

XFAC_S TI 1.781817

ALPB_CL TI 1.953656

XFAC_CL TI 0.831301

ALPB_CA TI 1.268314

XFAC_CA TI 0.513504

ALPB_TI TI 2.445684

XFAC_TI TI 29.795082

USS V -32.598954

UPP V -20.496422

UDD V -43.169867

BETAS V -4.628385

BETAP V -3.039568

BETAD V -3.704203

ZS V 6.051795

ZP V 2.249871

ZD V 1.087345

ZSN V 1.215500

ZPN V 0.877260

ZDN V 1.512555

GSS V 6.645015

GSP V 5.436952

GPP V 5.225810

GP2 V 4.580932

HSP V 1.092636

F0SD V 6.560730

G2SD V 1.196816

ALPB_H V 1.454900

XFAC_H V 0.350807

ALPB_C V 1.904429

XFAC_C V 0.489034

ALPB_N V 2.139547

XFAC_N V 0.964593

ALPB_O V 2.076717

XFAC_O V 0.789091

ALPB_F V 2.483525

XFAC_F V 1.056377

ALPB_NA V 2.548904

XFAC_NA V 8.346697

ALPB_P V 2.205190

XFAC_P V 6.763663

ALPB_S V 2.407934

XFAC_S V 1.374332

ALPB_CL V 2.395745

XFAC_CL V 1.590959

ALPB_K V 1.361275

XFAC_K V 1.893631

ALPB_V V 1.859935

XFAC_V V 0.953942

USS CR -41.077064

UPP CR -19.350873

UDD CR -80.190851

BETAS CR -13.781066

BETAP CR 0.735757

BETAD CR -6.372908

ZS CR 2.838413

ZP CR 1.379560

ZD CR 1.188729

ZSN CR 2.174521

ZPN CR 4.770642

ZDN CR 2.141579

GSS CR 11.887886

GSP CR 14.518298

GPP CR 28.418564

GP2 CR 24.911643

HSP CR 1.187458

F0SD CR 7.511007

G2SD CR 2.622589

ALPB_H CR 1.710489

XFAC_H CR 0.451845

ALPB_LI CR 1.554282

XFAC_LI CR 1.523425

ALPB_C CR 2.200250

XFAC_C CR 0.723497

ALPB_N CR 1.978476

XFAC_N CR 0.431966

ALPB_O CR 2.226688

XFAC_O CR 0.603066

ALPB_F CR 2.545695

XFAC_F CR 0.581501

ALPB_NA CR 1.742438

XFAC_NA CR 7.141413

ALPB_MG CR 1.949255

XFAC_MG CR 9.004042

ALPB_SI CR 1.632536

XFAC_SI CR 1.831750

ALPB_P CR 0.965663

XFAC_P CR 0.488071

ALPB_S CR 2.022399

XFAC_S CR 0.610052

ALPB_CL CR 2.494604

XFAC_CL CR 0.987014

ALPB_K CR 1.827441

XFAC_K CR 14.122878

ALPB_CA CR 1.748419

XFAC_CA CR 3.971766

ALPB_CR CR 2.859778

XFAC_CR CR 21.294482

USS MN -42.374682

UPP MN -18.304981

UDD MN -54.430991

BETAS MN -19.986721

BETAP MN -51.153604

BETAD MN -28.049908

ZS MN 1.666440

ZP MN 2.078735

ZD MN 2.897070

ZSN MN 1.299761

ZPN MN 4.059245

ZDN MN 1.146085

GSS MN 7.105662

GSP MN 8.807648

GPP MN 24.180795

GP2 MN 21.196825

HSP MN 0.221872

F0SD MN 4.784190

G2SD MN 2.008311

ALPB_H MN 1.815287

XFAC_H MN 1.334984

ALPB_C MN 2.122570

XFAC_C MN 1.646822

ALPB_N MN 2.625097

XFAC_N MN 2.366982

ALPB_O MN 3.225970

XFAC_O MN 3.636943

ALPB_F MN 3.508953

XFAC_F MN 2.404476

ALPB_AL MN 1.231200

XFAC_AL MN 1.130368

ALPB_SI MN 1.881580

XFAC_SI MN 3.934609

ALPB_P MN 1.879268

XFAC_P MN 5.259289

ALPB_S MN 2.205580

XFAC_S MN 2.583375

ALPB_CL MN 2.275167

XFAC_CL MN 2.025304

ALPB_K MN 1.328545

XFAC_K MN 1.921563

ALPB_CA MN 1.298445

XFAC_CA MN 0.520488

ALPB_TI MN 1.633575

XFAC_TI MN 4.212201

ALPB_MN MN 2.502150

XFAC_MN MN 23.014869

USS FE -74.715611

UPP FE -56.758188

UDD FE -90.918476

BETAS FE -4.365430

BETAP FE -4.256080

BETAD FE -12.531631

ZS FE 1.157576

ZP FE 2.737621

ZD FE 1.860792

ZSN FE 2.223065

ZPN FE 1.314405

ZDN FE 1.769722

GSS FE 12.153271

GSP FE 8.511068

GPP FE 7.829869

GP2 FE 6.863644

HSP FE 1.267977

POC FE 0.993526

F0SD FE 9.314037

G2SD FE 1.970401

ALPB_H FE 2.325000

XFAC_H FE 0.797044

ALPB_C FE 2.439391

XFAC_C FE 0.840113

ALPB_N FE 2.710121

XFAC_N FE 1.307687

ALPB_O FE 2.977229

XFAC_O FE 1.669098

ALPB_F FE 3.266034

XFAC_F FE 1.572783

ALPB_SI FE 2.261269

XFAC_SI FE 1.302779

ALPB_P FE 1.425836

XFAC_P FE 0.597968

ALPB_S FE 2.922342

XFAC_S FE 3.055008

ALPB_CL FE 2.803764

XFAC_CL FE 1.475990

ALPB_K FE 0.914983

XFAC_K FE 0.471163

ALPB_TI FE 2.152071

XFAC_TI FE 1.718797

ALPB_CR FE 2.320197

XFAC_CR FE 1.605266

ALPB_FE FE 3.253806

XFAC_FE FE 25.101048

USS CO -37.720682

UPP CO -0.230340

UDD CO -85.185900

BETAS CO -11.175136

BETAP CO -18.331339

BETAD CO -5.935777

ZS CO 1.789441

ZP CO 1.531664

ZD CO 1.951497

ZSN CO 1.710796

ZPN CO 0.928007

ZDN CO 1.563753

GSS CO 9.352749

GSP CO 6.087093

GPP CO 5.528108

GP2 CO 4.845926

HSP CO 0.763102

POC CO 1.433458

F0SD CO 3.045500

G2SD CO 1.015102

ALPB_H CO 2.212022

XFAC_H CO 0.781287

ALPB_LI CO 1.930303

XFAC_LI CO 0.523612

ALPB_B CO 3.200000

XFAC_B CO 1.000000

ALPB_C CO 1.369735

XFAC_C CO 0.101941

ALPB_N CO 2.018692

XFAC_N CO 0.371117

ALPB_O CO 2.512985

XFAC_O CO 0.617937

ALPB_F CO 3.169014

XFAC_F CO 1.042929

ALPB_NA CO 1.130004

XFAC_NA CO 0.525429

ALPB_SI CO 2.247195

XFAC_SI CO 1.130253

ALPB_P CO 2.298868

XFAC_P CO 3.189088

ALPB_S CO 2.144853

XFAC_S CO 0.522339

ALPB_CL CO 2.604673

XFAC_CL CO 0.979572

ALPB_K CO 1.347379

XFAC_K CO 1.363649

ALPB_CR CO 1.965685

XFAC_CR CO 0.907585

ALPB_CO CO 1.072023

XFAC_CO CO 0.082968

USS NI -55.503570

UPP NI -30.601744

UDD NI -68.610896

BETAS NI -15.417178

BETAP NI -21.305796

BETAD NI -4.094535

ZS NI 1.708340

ZP NI 2.000099

ZD NI 5.698724

ZSN NI 1.177087

ZPN NI 1.013217

ZDN NI 1.017987

GSS NI 6.435016

GSP NI 5.921995

GPP NI 6.035702

GP2 NI 5.290881

HSP NI 1.379687

POC NI 2.208500

F0SD NI 5.492550

G2SD NI 2.469437

ALPB_H NI 1.921141

XFAC_H NI 0.694497

ALPB_B NI 2.332207

XFAC_B NI 0.529685

ALPB_C NI 2.135123

XFAC_C NI 0.429059

ALPB_N NI 2.259589

XFAC_N NI 0.403691

ALPB_O NI 2.452312

XFAC_O NI 0.284888

ALPB_F NI 3.145389

XFAC_F NI 0.559407

ALPB_SI NI 2.260625

XFAC_SI NI 3.024544

ALPB_P NI 1.646184

XFAC_P NI 0.793563

ALPB_S NI 2.360866

XFAC_S NI 0.923582

ALPB_CL NI 2.771621

XFAC_CL NI 1.509842

ALPB_K NI 1.110139

XFAC_K NI 0.642360

ALPB_CR NI 2.774356

XFAC_CR NI 29.999969

ALPB_NI NI 1.626235

XFAC_NI NI 0.339558

USS CU -55.174441

UPP CU 3.200458

UDD CU -118.258961

BETAS CU -11.801588

BETAP CU -37.165178

BETAD CU -14.652492

ZS CU 1.735325

ZP CU 3.219976

ZD CU 6.013523

ZSN CU 2.419271

ZPN CU 0.302125

ZDN CU 1.678203

GSS CU 13.225910

GSP CU 2.055274

GPP CU 1.799749

GP2 CU 1.577656

HSP CU 0.000420

F0SD CU 5.160900

G2SD CU 2.792359

ALPB_H CU 2.941555

XFAC_H CU 1.781622

ALPB_C CU 3.018944

XFAC_C CU 1.413488

ALPB_N CU 2.566300

XFAC_N CU 0.429906

ALPB_O CU 1.911057

XFAC_O CU 0.098068

ALPB_F CU 3.176529

XFAC_F CU 0.411293

ALPB_NA CU 1.306695

XFAC_NA CU 0.785487

ALPB_AL CU 2.320517

XFAC_AL CU 12.995965

ALPB_P CU 0.858794

XFAC_P CU 5.035151

ALPB_S CU 2.053844

XFAC_S CU 0.296518

ALPB_CL CU 2.475894

XFAC_CL CU 0.372668

ALPB_K CU 2.087357

XFAC_K CU 7.795310

ALPB_CU CU 3.103277

XFAC_CU CU 3.391704

USS ZN -16.700035

UPP ZN -14.844247

BETAS ZN -16.770975

BETAP ZN 2.907797

ZS ZN 1.560140

ZP ZN 1.915631

GSS ZN 6.421475

GSP ZN 10.243652

GPP ZN 20.001326

GP2 ZN 16.126802

HSP ZN 0.983644

ALPB_H ZN 1.874800

XFAC_H ZN 1.696831

ALPB_C ZN 2.171605

XFAC_C ZN 2.386580

ALPB_N ZN 1.805998

XFAC_N ZN 0.900539

ALPB_O ZN 2.079887

XFAC_O ZN 1.116990

ALPB_F ZN 1.859561

XFAC_F ZN 0.499581

ALPB_NA ZN 1.588584

XFAC_NA ZN 5.694720

ALPB_SI ZN 1.890360

XFAC_SI ZN 6.865738

ALPB_P ZN 1.398572

XFAC_P ZN 1.863594

ALPB_S ZN 1.379514

XFAC_S ZN 0.533478

ALPB_CL ZN 1.588143

XFAC_CL ZN 0.547720

ALPB_CA ZN 0.974041

XFAC_CA ZN 1.296565

ALPB_V ZN 1.513777

XFAC_V ZN 1.442270

ALPB_CR ZN 2.071878

XFAC_CR ZN 2.312424

ALPB_ZN ZN 1.998115

XFAC_ZN ZN 19.124599

USS GA -30.812730

UPP GA -22.498885

BETAS GA -15.082480

BETAP GA -0.938845

ZS GA 1.913326

ZP GA 1.811217

GSS GA 9.436450

GSP GA 9.189262

GPP GA 5.480436

GP2 GA 6.991064

HSP GA 0.970992

ALPB_H GA 2.170771

XFAC_H GA 2.091955

ALPB_C GA 2.188866

XFAC_C GA 1.617568

ALPB_N GA 1.949999

XFAC_N GA 0.867734

ALPB_O GA 2.408216

XFAC_O GA 1.379976

ALPB_F GA 3.055971

XFAC_F GA 2.319957

ALPB_SI GA 2.169690

XFAC_SI GA 5.031330

ALPB_P GA 1.600000

XFAC_P GA 4.000000

ALPB_S GA 2.514000

XFAC_S GA 4.204343

ALPB_CL GA 2.104228

XFAC_CL GA 1.129276

ALPB_GA GA 2.390223

XFAC_GA GA 11.941483

USS GE -35.694620

UPP GE -29.273804

BETAS GE -18.071730

BETAP GE -1.563157

ZS GE 2.762845

ZP GE 1.531131

GSS GE 4.991616

GSP GE 9.108444

GPP GE 6.693916

GP2 GE 5.914950

HSP GE 0.801760

ALPB_H GE 2.470301

XFAC_H GE 2.398259

ALPB_C GE 2.351577

XFAC_C GE 1.605487

ALPB_N GE 2.239698

XFAC_N GE 1.028521

ALPB_O GE 2.217395

XFAC_O GE 0.690557

ALPB_F GE 1.727325

XFAC_F GE 0.165644

ALPB_SI GE 2.053934

XFAC_SI GE 3.121907

ALPB_P GE 1.831652

XFAC_P GE 4.212771

ALPB_S GE 2.358433

XFAC_S GE 1.947726

ALPB_CL GE 2.506796

XFAC_CL GE 1.783333

ALPB_MN GE 1.937769

XFAC_MN GE 2.470135

ALPB_CO GE 2.852610

XFAC_CO GE 2.151850

ALPB_GE GE 2.215455

XFAC_GE GE 5.884206

USS AS -41.523302

UPP AS -36.959219

UDD AS -35.859071

BETAS AS -17.440295

BETAP AS -6.603566

BETAD AS -2.445554

ZS AS 3.213850

ZP AS 1.628384

ZD AS 3.314358

ZSN AS 0.916221

ZPN AS 1.115722

ZDN AS 2.137809

GSS AS 8.088350

GSP AS 7.457692

GPP AS 8.918517

GP2 AS 7.102449

HSP AS 0.969630

ALPB_H AS 1.749762

XFAC_H AS 0.763924

ALPB_C AS 1.805305

XFAC_C AS 0.604465

ALPB_N AS 2.035339

XFAC_N AS 0.784041

ALPB_O AS 2.387990

XFAC_O AS 1.076670

ALPB_F AS 2.783517

XFAC_F AS 1.196884

ALPB_NA AS 1.763497

XFAC_NA AS 2.673425

ALPB_AL AS 1.332670

XFAC_AL AS 1.322056

ALPB_SI AS 1.771030

XFAC_SI AS 1.384298

ALPB_S AS 1.826372

XFAC_S AS 0.732648

ALPB_CL AS 1.947927

XFAC_CL AS 0.747436

ALPB_K AS 1.267957

XFAC_K AS 2.276204

ALPB_TI AS 1.711955

XFAC_TI AS 1.371503

ALPB_CO AS 1.514923

XFAC_CO AS 2.030232

ALPB_ZN AS 1.618734

XFAC_ZN AS 2.700385

ALPB_GA AS 1.534812

XFAC_GA AS 1.196640

ALPB_AS AS 1.707277

XFAC_AS AS 1.325873

USS SE -47.218303

UPP SE -35.588294

BETAS SE -9.614158

BETAP SE -6.121302

ZS SE 2.751130

ZP SE 1.901764

GSS SE 4.895424

GSP SE 6.792977

GPP SE 5.775063

GP2 SE 5.578480

HSP SE 3.152775

ALPB_H SE 2.547234

XFAC_H SE 1.229099

ALPB_C SE 2.186857

XFAC_C SE 0.654796

ALPB_N SE 1.980885

XFAC_N SE 0.448537

ALPB_O SE 2.612643

XFAC_O SE 0.860233

ALPB_F SE 2.463196

XFAC_F SE 0.473969

ALPB_NA SE 1.115555

XFAC_NA SE 0.902628

ALPB_SI SE 2.318601

XFAC_SI SE 2.051717

ALPB_P SE 1.865719

XFAC_P SE 2.359419

ALPB_S SE 1.492756

XFAC_S SE 0.530796

ALPB_CL SE 2.170000

XFAC_CL SE 0.869163

ALPB_K SE 1.680151

XFAC_K SE 3.871380

ALPB_MN SE 1.981410

XFAC_MN SE 2.170787

ALPB_CO SE 2.523450

XFAC_CO SE 2.202410

ALPB_ZN SE 1.163289

XFAC_ZN SE 0.367711

ALPB_GE SE 1.604107

XFAC_GE SE 0.556002

ALPB_AS SE 1.514823

XFAC_AS SE 0.541956

ALPB_SE SE 1.524158

XFAC_SE SE 0.334506

USS BR -49.141354

UPP BR -48.274409

UDD BR 2.677328

BETAS BR -32.458894

BETAP BR -10.270309

BETAD BR -19.977175

ZS BR 3.725480

ZP BR 2.242318

ZD BR 1.591034

ZSN BR 10.522069

ZPN BR 9.531017

ZDN BR 5.776829

GSS BR 8.131665

GSP BR 4.285572

GPP BR 8.056519

GP2 BR 7.520115

HSP BR 1.567275

ALPB_H BR 2.339252

XFAC_H BR 1.270390

ALPB_HE BR 2.127598

XFAC_HE BR 1.062013

ALPB_LI BR 2.143819

XFAC_LI BR 2.241404

ALPB_BE BR 2.283569

XFAC_BE BR 2.659130

ALPB_B BR 2.307098

XFAC_B BR 1.849590

ALPB_C BR 2.252349

XFAC_C BR 0.968921

ALPB_N BR 3.015469

XFAC_N BR 4.148435

ALPB_O BR 2.739280

XFAC_O BR 1.425004

ALPB_F BR 2.753002

XFAC_F BR 0.915809

ALPB_NE BR 2.483203

XFAC_NE BR 1.001506

ALPB_NA BR 2.327183

XFAC_NA BR 11.511433

ALPB_MG BR 2.350023

XFAC_MG BR 7.183008

ALPB_AL BR 1.514344

XFAC_AL BR 1.504277

ALPB_SI BR 1.715815

XFAC_SI BR 1.126731

ALPB_P BR 1.664343

XFAC_P BR 1.746224

ALPB_S BR 2.099922

XFAC_S BR 1.004759

ALPB_CL BR 1.906403

XFAC_CL BR 0.581542

ALPB_AR BR 2.454724

XFAC_AR BR 3.261699

ALPB_K BR 1.887799

XFAC_K BR 7.523969

ALPB_CA BR 2.558257

XFAC_CA BR 12.875179

ALPB_SC BR 1.531278

XFAC_SC BR 1.063920

ALPB_TI BR 1.760015

XFAC_TI BR 1.534465

ALPB_V BR 1.909502

XFAC_V BR 1.394543

ALPB_CR BR 1.781866

XFAC_CR BR 0.746857

ALPB_MN BR 2.183298

XFAC_MN BR 2.530861

ALPB_FE BR 2.388196

XFAC_FE BR 1.415413

ALPB_CO BR 2.124301

XFAC_CO BR 0.759690

ALPB_NI BR 2.543159

XFAC_NI BR 1.656698

ALPB_CU BR 3.040037

XFAC_CU BR 2.655465

ALPB_ZN BR 1.594962

XFAC_ZN BR 1.241996

ALPB_GA BR 1.934418

XFAC_GA BR 1.722754

ALPB_GE BR 2.062366

XFAC_GE BR 2.031652

ALPB_AS BR 1.750449

XFAC_AS BR 0.949379

ALPB_SE BR 1.788806

XFAC_SE BR 0.682982

ALPB_BR BR 2.147378

XFAC_BR BR 1.599562

USS KR 8.535384

UPP KR -80.484321

BETAS KR -2.727088

BETAP KR -16.142951

ZS KR 1.312248

ZP KR 4.491371

GSS KR 19.999857

GSP KR 1.175304

GPP KR 9.174784

GP2 KR 14.926948

HSP KR 0.299867

ALPB_H KR 3.770453

XFAC_H KR 5.125897

ALPB_HE KR 1.996943

XFAC_HE KR 0.627701

ALPB_LI KR 3.004783

XFAC_LI KR 8.377143

ALPB_BE KR 3.289764

XFAC_BE KR 10.264026

ALPB_B KR 2.559201

XFAC_B KR 2.931148

ALPB_C KR 2.076738

XFAC_C KR 0.652623

ALPB_N KR 1.644052

XFAC_N KR 0.199606

ALPB_O KR 0.297001

ALPB_F KR 3.452321

XFAC_F KR 4.134407

ALPB_NE KR 2.813679

XFAC_NE KR 1.433722

ALPB_NA KR 2.562062

XFAC_NA KR 9.817859

ALPB_MG KR 1.296221

XFAC_MG KR 1.119449

ALPB_AL KR 2.493834

XFAC_AL KR 5.076857

ALPB_SI KR 1.545354

XFAC_SI KR 0.639030

ALPB_CL KR 1.884662

XFAC_CL KR 0.520353

ALPB_AR KR 1.995125

XFAC_AR KR 0.554874

ALPB_K KR 2.296640

XFAC_K KR 8.532309

ALPB_CA KR 1.559229

XFAC_CA KR 1.305808

ALPB_BR KR 1.608300

XFAC_BR KR 0.499653

ALPB_KR KR 1.913342

XFAC_KR KR 0.252431

USS RB -4.120962

UPP RB -0.633300

BETAS RB -8.442947

BETAP RB 4.853952

ZS RB 1.314831

ZP RB 6.015581

GSS RB 11.892047

GSP RB 3.477383

GPP RB 6.000901

GP2 RB 6.008182

HSP RB 0.998126

ALPB_H RB 1.890495

XFAC_H RB 4.316836

ALPB_HE RB 1.543436

XFAC_HE RB 1.804024

ALPB_B RB 2.989999

XFAC_B RB 10.280532

ALPB_C RB 2.287377

XFAC_C RB 24.603216

ALPB_N RB 2.205212

XFAC_N RB 19.919815

ALPB_O RB 1.572166

XFAC_O RB 0.791546

ALPB_F RB 3.131045

XFAC_F RB 2.629683

ALPB_NE RB 2.429710

XFAC_NE RB 7.683406

ALPB_AL RB 0.931060

XFAC_AL RB 19.138062

ALPB_P RB 0.922429

XFAC_P RB 0.526907

ALPB_S RB 1.285680

XFAC_S RB 1.380751

ALPB_CL RB 1.349244

XFAC_CL RB 0.714916

ALPB_AR RB 2.581073

XFAC_AR RB 18.431817

ALPB_K RB 1.719577

XFAC_K RB 1.174003

ALPB_V RB 2.024277

XFAC_V RB 12.360809

ALPB_CR RB 1.569973

XFAC_CR RB 5.846691

ALPB_BR RB 1.737339

XFAC_BR RB 7.887658

ALPB_KR RB 2.413547

XFAC_KR RB 15.315026

ALPB_RB RB 0.684616

XFAC_RB RB 4.280624

USS SR -10.693066

UPP SR -8.539218

BETAS SR -4.904378

BETAP SR 8.809297

ZS SR 2.092264

ZP SR 3.314082

GSS SR 6.494973

GSP SR 4.045506

GPP SR 2.547611

GP2 SR 4.121201

HSP SR 1.102790

ALPB_H SR 2.918082

XFAC_H SR 18.457472

ALPB_C SR 2.802150

XFAC_C SR 11.414713

ALPB_N SR 3.025117

XFAC_N SR 14.103810

ALPB_O SR 3.921499

XFAC_O SR 17.067149

ALPB_F SR 3.309755

XFAC_F SR 3.758667

ALPB_SI SR 2.416117

XFAC_SI SR 29.996030

ALPB_P SR 2.273841

XFAC_P SR 23.946650

ALPB_S SR 3.325295

XFAC_S SR 41.563327

ALPB_CL SR 3.501974

XFAC_CL SR 39.960719

ALPB_TI SR 2.880030

XFAC_TI SR 2.817250

ALPB_GA SR 1.489463

XFAC_GA SR 2.800419

ALPB_BR SR 3.086374

XFAC_BR SR 19.218824

ALPB_SR SR 2.194036

XFAC_SR SR 31.817350

USS Y -17.035117

UPP Y -16.168689

UDD Y -16.354811

BETAS Y -10.513848

BETAP Y -11.341408

BETAD Y -10.701025

ZS Y 1.605083

ZP Y 2.131069

ZD Y 6.021645

ZSN Y 1.186263

ZPN Y 2.244351

ZDN Y 0.911477

GSS Y 5.318448

GSP Y 6.318665

GPP Y 11.005171

GP2 Y 9.590777

HSP Y 0.637634

POC Y 2.019557

F0SD Y 6.855595

G2SD Y 5.889822

ALPB_H Y 2.322175

XFAC_H Y 6.935667

ALPB_LI Y 1.212009

XFAC_LI Y 0.577598

ALPB_C Y 2.541211

XFAC_C Y 19.957240

ALPB_N Y 2.084245

XFAC_N Y 3.253368

ALPB_O Y 2.086475

XFAC_O Y 1.424444

ALPB_F Y 3.245964

XFAC_F Y 9.257528

ALPB_AL Y 1.003500

XFAC_AL Y 0.500670

ALPB_SI Y 2.016820

XFAC_SI Y 3.219030

ALPB_P Y 1.172165

XFAC_P Y 1.726458

ALPB_S Y 1.345475

XFAC_S Y 0.961448

ALPB_CL Y 1.882700

XFAC_CL Y 2.186706

ALPB_K Y 0.947193

XFAC_K Y 1.143281

ALPB_BR Y 1.359600

XFAC_BR Y 1.090173

ALPB_Y Y 1.533049

XFAC_Y Y 15.620872

USS ZR -18.679203

UPP ZR -0.049727

UDD ZR -23.299157

BETAS ZR 1.688872

BETAP ZR 2.330045

BETAD ZR -4.552268

ZS ZR 1.373517

ZP ZR 1.141705

ZD ZR 1.618769

ZSN ZR 1.082243

ZPN ZR 2.978817

ZDN ZR 1.417227

GSS ZR 4.852089

GSP ZR 5.870299

GPP ZR 14.606623

GP2 ZR 12.729368

HSP ZR 0.151363

F0SD ZR 4.737734

G2SD ZR 2.141620

ALPB_H ZR 1.536594

XFAC_H ZR 0.414278

ALPB_C ZR 1.738320

XFAC_C ZR 0.715392

ALPB_N ZR 1.986255

XFAC_N ZR 1.386137

ALPB_O ZR 2.093741

XFAC_O ZR 1.293822

ALPB_F ZR 2.406399

XFAC_F ZR 1.606098

ALPB_AL ZR 1.270620

XFAC_AL ZR 0.874060

ALPB_SI ZR 1.605795

XFAC_SI ZR 1.566491

ALPB_P ZR 0.963910

XFAC_P ZR 1.020015

ALPB_S ZR 0.957666

XFAC_S ZR 0.200522

ALPB_CL ZR 2.352409

XFAC_CL ZR 2.273630

ALPB_BR ZR 1.617591

XFAC_BR ZR 1.243772

ALPB_ZR ZR 2.714671

XFAC_ZR ZR 29.768192

USS NB -33.497110

UPP NB -34.762698

UDD NB -44.819149

BETAS NB -23.566737

BETAP NB -1.623945

BETAD NB -7.421668

ZS NB 2.761686

ZP NB 5.999062

ZD NB 1.611677

ZSN NB 1.429235

ZPN NB 2.911794

ZDN NB 1.950434

GSS NB 6.407780

GSP NB 7.659444

GPP NB 14.277976

GP2 NB 12.442959

HSP NB 0.619230

F0SD NB 6.393769

G2SD NB 1.759636

ALPB_H NB 2.321651

XFAC_H NB 6.958727

ALPB_C NB 2.277928

XFAC_C NB 1.991488

ALPB_N NB 2.810017

XFAC_N NB 4.374260

ALPB_O NB 2.715670

XFAC_O NB 2.793681

ALPB_F NB 3.115376

XFAC_F NB 3.297982

ALPB_NA NB 2.551010

XFAC_NA NB 8.276020

ALPB_P NB 1.922968

XFAC_P NB 6.219347

ALPB_S NB 2.279550

XFAC_S NB 3.225637

ALPB_CL NB 2.757523

XFAC_CL NB 6.452483

ALPB_K NB 4.521360

XFAC_K NB 2.026590

ALPB_BR NB 2.531918

XFAC_BR NB 8.316457

ALPB_NB NB 2.030464

XFAC_NB NB 10.027153

USS MO -51.662768

UPP MO 46.059429

UDD MO -57.269405

BETAS MO 6.685073

BETAP MO 5.485123

BETAD MO -13.146960

ZS MO 1.595399

ZP MO 1.426575

ZD MO 1.787748

ZSN MO 1.903541

ZPN MO 1.592195

ZDN MO 1.889678

GSS MO 8.534266

GSP MO 7.704937

GPP MO 7.807325

GP2 MO 6.803921

HSP MO 1.787407

F0SD MO 9.654475

G2SD MO 2.314954

ALPB_H MO 2.139004

XFAC_H MO 1.177934

ALPB_LI MO 2.201335

XFAC_LI MO 5.209247

ALPB_C MO 2.140063

XFAC_C MO 1.042667

ALPB_N MO 2.293955

XFAC_N MO 1.330858

ALPB_O MO 2.197353

XFAC_O MO 0.864597

ALPB_F MO 2.593518

XFAC_F MO 1.107779

ALPB_NA MO 2.440770

XFAC_NA MO 8.286550

ALPB_P MO 1.850441

XFAC_P MO 1.522846

ALPB_S MO 2.343350

XFAC_S MO 1.822187

ALPB_CL MO 2.358706

XFAC_CL MO 1.570824

ALPB_K MO 1.594941

XFAC_K MO 10.522232

ALPB_CR MO 1.873232

XFAC_CR MO 0.484096

ALPB_FE MO 2.239581

XFAC_FE MO 3.257115

ALPB_BR MO 1.934589

XFAC_BR MO 1.291525

ALPB_RB MO 2.971399

XFAC_RB MO 0.874676

ALPB_MO MO 1.078447

XFAC_MO MO 0.223956

USS TC -48.916740

UPP TC -21.908166

UDD TC -53.807590

BETAS TC -17.096185

BETAP TC -17.740652

BETAD TC -7.241592

ZS TC 2.104672

ZP TC 2.669984

ZD TC 3.030496

ZSN TC 2.061082

ZPN TC 0.888524

ZDN TC 1.575315

GSS TC 9.240580

GSP TC 4.795964

GPP TC 4.356876

GP2 TC 3.796926

HSP TC 0.248174

F0SD TC 7.521148

G2SD TC 3.106149

ALPB_H TC 2.576199

XFAC_H TC 5.418951

ALPB_C TC 2.815972

XFAC_C TC 3.999428

ALPB_N TC 2.177956

XFAC_N TC 0.980071

ALPB_O TC 2.535619

XFAC_O TC 1.303538

ALPB_F TC 3.385092

XFAC_F TC 3.880884

ALPB_P TC 0.930051

XFAC_P TC 0.470758

ALPB_S TC 2.141702

XFAC_S TC 1.449910

ALPB_CL TC 2.360242

XFAC_CL TC 1.744657

ALPB_GE TC 2.852820

XFAC_GE TC 2.152060

ALPB_SE TC 2.523660

XFAC_SE TC 2.202620

ALPB_BR TC 2.688330

XFAC_BR TC 6.426037

ALPB_TC TC 2.153000

XFAC_TC TC 2.572063

USS RU -41.151429

UPP RU -42.965344

UDD RU -45.714719

BETAS RU -4.989393

BETAP RU -10.778690

BETAD RU 1.216566

ZS RU 1.605646

ZP RU 4.580820

ZD RU 1.244578

ZSN RU 1.172546

ZPN RU 1.373361

ZDN RU 1.018114

GSS RU 5.256950

GSP RU 5.631870

GPP RU 6.734273

GP2 RU 5.868779

HSP RU 1.326657

F0SD RU 4.898881

G2SD RU 2.648488

ALPB_H RU 3.031003

XFAC_H RU 0.462490

ALPB_C RU 2.661734

XFAC_C RU 0.434352

ALPB_N RU 1.951233

XFAC_N RU 0.271221

ALPB_O RU 1.928484

XFAC_O RU 0.339590

ALPB_F RU 2.719488

XFAC_F RU 0.680978

ALPB_SI RU 2.775910

XFAC_SI RU 0.849430

ALPB_P RU 1.440298

XFAC_P RU 0.482587

ALPB_S RU 3.002139

XFAC_S RU 0.788319

ALPB_CL RU 3.340740

XFAC_CL RU 1.986295

ALPB_GE RU 2.852320

XFAC_GE RU 2.151560

ALPB_SE RU 2.523160

XFAC_SE RU 2.202120

ALPB_BR RU 2.611647

XFAC_BR RU 3.893512

ALPB_RU RU 2.341541

XFAC_RU RU 0.984874

USS RH -24.613157

UPP RH 6.621039

UDD RH -81.764165

BETAS RH -9.488908

BETAP RH -6.699556

BETAD RH -7.997845

ZS RH 1.591465

ZP RH 4.546046

ZD RH 2.685918

ZSN RH 2.079986

ZPN RH 9.641003

ZDN RH 1.787794

GSS RH 9.325333

GSP RH 11.318440

GPP RH 47.274639

GP2 RH 41.198863

HSP RH 0.017585

F0SD RH 2.230584

G2SD RH 1.492841

ALPB_H RH 2.716287

XFAC_H RH 1.728302

ALPB_B RH 2.400000

XFAC_B RH 2.000000

ALPB_C RH 3.007700

XFAC_C RH 0.562962

ALPB_N RH 3.028135

XFAC_N RH 1.013618

ALPB_O RH 3.452408

XFAC_O RH 1.534037

ALPB_F RH 3.083507

XFAC_F RH 0.772245

ALPB_SI RH 2.776490

XFAC_SI RH 0.850010

ALPB_P RH 2.236601

XFAC_P RH 0.738916

ALPB_S RH 3.005420

XFAC_S RH 0.970563

ALPB_CL RH 3.542676

XFAC_CL RH 0.628186

ALPB_GE RH 2.852900

XFAC_GE RH 2.152140

ALPB_SE RH 2.523740

XFAC_SE RH 2.202700

ALPB_BR RH 2.893677

XFAC_BR RH 1.441509

ALPB_RH RH 3.281577

XFAC_RH RH 17.154616

USS PD -90.670356

UPP PD 45.018147

UDD PD -94.618031

BETAS PD -18.862423

BETAP PD -18.107010

BETAD PD -3.592862

ZS PD 5.790768

ZP PD 2.169788

ZD PD 1.327661

ZSN PD 1.985663

ZPN PD 0.621281

ZDN PD 1.768258

GSS PD 8.902449

GSP PD 3.376439

GPP PD 3.046450

GP2 PD 2.654918

HSP PD 0.043028

F0SD PD 9.251409

G2SD PD 1.948722

ALPB_H PD 3.052992

XFAC_H PD 0.675244

ALPB_C PD 1.449994

XFAC_C PD 0.040769

ALPB_N PD 2.319285

XFAC_N PD 0.327063

ALPB_O PD 2.362481

XFAC_O PD 0.394849

ALPB_F PD 3.117188

XFAC_F PD 0.610235

ALPB_AL PD 1.572720

XFAC_AL PD 1.057290

ALPB_SI PD 2.714212

XFAC_SI PD 1.381243

ALPB_P PD 0.876896

XFAC_P PD 0.223289

ALPB_S PD 3.134436

XFAC_S PD 0.568359

ALPB_CL PD 2.966363

XFAC_CL PD 0.764165

ALPB_BR PD 2.087790

XFAC_BR PD 0.491172

ALPB_PD PD 1.712149

XFAC_PD PD 0.297913

USS AG -92.280499

UPP AG 29.229985

UDD AG -82.344865

BETAS AG -9.850776

BETAP AG -29.894728

BETAD AG -63.636331

ZS AG 1.793032

ZP AG 2.528721

ZD AG 3.524808

ZSN AG 1.619764

ZPN AG 0.439729

ZDN AG 1.210202

GSS AG 7.261991

GSP AG 2.391732

GPP AG 2.156210

GP2 AG 1.879092

HSP AG 0.014435

F0SD AG 8.987758

G2SD AG 4.716654

ALPB_H AG 1.866268

XFAC_H AG 0.669745

ALPB_B AG 1.454270

XFAC_B AG 2.733745

ALPB_C AG 2.401775

XFAC_C AG 1.108319

ALPB_N AG 2.835438

XFAC_N AG 1.090232

ALPB_O AG 2.453629

XFAC_O AG 0.372450

ALPB_F AG 3.119532

XFAC_F AG 0.897783

ALPB_AL AG 1.683750

XFAC_AL AG 1.093559

ALPB_P AG 1.305572

XFAC_P AG 0.482631

ALPB_S AG 2.575670

XFAC_S AG 1.817701

ALPB_CL AG 3.198107

XFAC_CL AG 3.386746

ALPB_K AG 2.092259

XFAC_K AG 5.619211

ALPB_CR AG 2.700428

XFAC_CR AG 21.083639

ALPB_BR AG 3.259287

XFAC_BR AG 6.111850

ALPB_PD AG 4.000000

XFAC_PD AG 2.000000

ALPB_AG AG 1.489404

XFAC_AG AG 0.178879

USS CD -18.127987

UPP CD -13.777839

BETAS CD -23.781665

BETAP CD -11.892060

ZS CD 3.670047

ZP CD 1.857036

GSS CD 8.904816

GSP CD 9.232666

GPP CD 11.103045

GP2 CD 10.905897

HSP CD 0.981926

ALPB_H CD 1.875490

XFAC_H CD 3.377913

ALPB_C CD 1.940388

XFAC_C CD 3.855359

ALPB_N CD 1.769441

XFAC_N CD 1.481460

ALPB_O CD 2.668165

XFAC_O CD 5.349517

ALPB_F CD 3.174783

XFAC_F CD 8.351869

ALPB_NA CD 2.000000

XFAC_NA CD 6.000000

ALPB_SI CD 1.286882

XFAC_SI CD 2.345912

ALPB_S CD 1.735391

XFAC_S CD 2.929257

ALPB_CL CD 1.870170

XFAC_CL CD 2.254752

ALPB_K CD 1.033580

XFAC_K CD 2.093242

ALPB_SE CD 1.881368

XFAC_SE CD 6.139995

ALPB_BR CD 1.918455

XFAC_BR CD 5.550415

ALPB_CD CD 1.428097

XFAC_CD CD 10.662907

USS IN -26.891944

UPP IN -28.519053

BETAS IN -0.447307

BETAP IN -4.269337

ZS IN 1.902085

ZP IN 1.940127

GSS IN 6.493621

GSP IN 12.576468

GPP IN 10.282533

GP2 IN 10.903195

HSP IN 2.133796

ALPB_H IN 1.852461

XFAC_H IN 1.773147

ALPB_B IN 1.735480

XFAC_B IN 1.951651

ALPB_C IN 1.810115

XFAC_C IN 1.041540

ALPB_N IN 2.052217

XFAC_N IN 1.529722

ALPB_O IN 2.178110

XFAC_O IN 1.467957

ALPB_F IN 2.319418

XFAC_F IN 1.018315

ALPB_S IN 2.430104

XFAC_S IN 4.796933

ALPB_CL IN 2.211880

XFAC_CL IN 2.224354

ALPB_GA IN 1.596053

XFAC_GA IN 2.473577

ALPB_AS IN 1.520977

XFAC_AS IN 1.375570

ALPB_SE IN 1.362364

XFAC_SE IN 0.598029

ALPB_BR IN 1.862313

XFAC_BR IN 2.138634

ALPB_RB IN 0.859259

XFAC_RB IN 4.688357

ALPB_IN IN 2.601789

XFAC_IN IN 24.204383

USS SN -33.880164

UPP SN -39.128186

BETAS SN 0.443105

BETAP SN -8.486074

ZS SN 1.959238

ZP SN 1.976146

GSS SN 6.196917

GSP SN 10.595744

GPP SN 14.691065

GP2 SN 13.501111

HSP SN 1.234523

ALPB_H SN 1.855042

XFAC_H SN 1.459969

ALPB_C SN 1.818782

XFAC_C SN 0.961947

ALPB_N SN 1.783560

XFAC_N SN 0.731228

ALPB_O SN 1.959102

XFAC_O SN 0.723272

ALPB_F SN 2.593459

XFAC_F SN 1.477352

ALPB_AL SN 1.597939

XFAC_AL SN 2.367990

ALPB_S SN 2.065722

XFAC_S SN 1.909070

ALPB_CL SN 1.887044

XFAC_CL SN 0.944374

ALPB_K SN 2.238329

XFAC_K SN 3.440153

ALPB_GE SN 2.016055

XFAC_GE SN 3.500376

ALPB_SE SN 1.393411

XFAC_SE SN 0.413851

ALPB_BR SN 1.594297

XFAC_BR SN 0.954605

ALPB_SN SN 1.045406

XFAC_SN SN 0.300460

USS SB -42.835901

UPP SB -19.996258

UDD SB -20.317174

BETAS SB -13.037071

BETAP SB -6.166480

BETAD SB -9.740725

ZS SB 1.998600

ZP SB 1.887062

ZD SB 1.475516

ZSN SB 2.179206

ZPN SB 0.862318

ZDN SB 4.147596

GSS SB 9.994149

GSP SB 1.434008

GPP SB 7.208157

GP2 SB 6.212730

HSP SB 3.566032

ALPB_H SB 1.091035

XFAC_H SB 0.408876

ALPB_C SB 1.240714

XFAC_C SB 0.327493

ALPB_N SB 0.846645

XFAC_N SB 0.137604

ALPB_O SB 1.462059

XFAC_O SB 0.346536

ALPB_F SB 1.622505

XFAC_F SB 0.283768

ALPB_NA SB 1.106800

XFAC_NA SB 0.547287

ALPB_AL SB 1.085906

XFAC_AL SB 1.291895

ALPB_SI SB 2.519702

XFAC_SI SB 8.707039

ALPB_S SB 1.016407

XFAC_S SB 0.211102

ALPB_CL SB 1.170710

XFAC_CL SB 0.217072

ALPB_MN SB 1.698753

XFAC_MN SB 2.384408

ALPB_CO SB 2.204630

XFAC_CO SB 2.276050

ALPB_BR SB 1.227775

XFAC_BR SB 0.567204

ALPB_TC SB 2.204850

XFAC_TC SB 2.276260

ALPB_RU SB 2.968084

XFAC_RU SB 2.509269

ALPB_RH SB 2.204930

XFAC_RH SB 2.276340

ALPB_IN SB 1.011173

XFAC_IN SB 0.470521

ALPB_SB SB 0.657753

XFAC_SB SB 0.219843

USS TE -97.416118

UPP TE -50.000552

BETAS TE -70.028904

BETAP TE -11.183348

ZS TE 3.024819

ZP TE 2.598283

GSS TE 18.350494

GSP TE 11.255114

GPP TE 8.695261

GP2 TE 7.622556

HSP TE 3.626912

ALPB_H TE 2.879705

XFAC_H TE 7.645321

ALPB_B TE 2.443355

XFAC_B TE 2.926026

ALPB_C TE 2.858205

XFAC_C TE 7.513380

ALPB_N TE 2.548060

XFAC_N TE 2.356842

ALPB_O TE 2.359294

XFAC_O TE 1.147602

ALPB_F TE 3.109030

XFAC_F TE 2.199214

ALPB_AL TE 1.783994

XFAC_AL TE 9.305330

ALPB_P TE 1.482343

XFAC_P TE 1.459960

ALPB_S TE 2.969323

XFAC_S TE 14.279019

ALPB_CL TE 1.475730

XFAC_CL TE 0.514830

ALPB_K TE 1.257635

XFAC_K TE 2.073198

ALPB_ZN TE 1.704782

XFAC_ZN TE 4.125260

ALPB_GE TE 2.049526

XFAC_GE TE 7.601044

ALPB_AS TE 1.275249

XFAC_AS TE 0.866529

ALPB_SE TE 1.585819

XFAC_SE TE 1.322800

ALPB_BR TE 2.316655

XFAC_BR TE 4.158560

ALPB_CD TE 1.759718

XFAC_CD TE 8.405812

ALPB_IN TE 1.913212

XFAC_IN TE 9.943252

ALPB_SN TE 2.265433

XFAC_SN TE 11.004064

ALPB_SB TE 1.634994

XFAC_SB TE 0.575666

ALPB_TE TE 3.032862

XFAC_TE TE 29.604279

USS I -63.618928

UPP I -45.760969

UDD I 6.109119

BETAS I -37.373318

BETAP I -10.174482

BETAD I -11.807267

ZS I 3.316202

ZP I 2.449124

ZD I 1.716121

ZSN I 4.000764

ZPN I 3.993847

ZDN I 3.946706

GSS I 7.658717

GSP I 8.237228

GPP I 5.667030

GP2 I 5.661068

HSP I 2.688576

ALPB_H I 2.301289

XFAC_H I 2.457731

ALPB_HE I 2.264096

XFAC_HE I 2.613098

ALPB_LI I 1.392191

XFAC_LI I 1.220335

ALPB_BE I 2.137694

XFAC_BE I 4.012926

ALPB_B I 1.949150

XFAC_B I 1.926808

ALPB_C I 2.105122

XFAC_C I 1.553541

ALPB_N I 2.204300

XFAC_N I 1.197247

ALPB_O I 2.031236

XFAC_O I 0.673908

ALPB_F I 2.168508

XFAC_F I 0.518622

ALPB_NE I 2.572520

XFAC_NE I 1.449278

ALPB_NA I 1.999781

XFAC_NA I 12.909796

ALPB_MG I 1.832289

XFAC_MG I 4.415343

ALPB_AL I 1.515624

XFAC_AL I 2.691541

ALPB_SI I 1.472015

XFAC_SI I 1.272495

ALPB_P I 1.560276

XFAC_P I 2.308251

ALPB_S I 2.108468

XFAC_S I 1.287638

ALPB_CL I 1.674480

XFAC_CL I 0.582734

ALPB_AR I 1.583967

XFAC_AR I 0.297828

ALPB_K I 1.527318

XFAC_K I 6.255639

ALPB_CA I 1.931292

XFAC_CA I 5.485613

ALPB_SC I 1.888645

XFAC_SC I 4.305507

ALPB_TI I 1.569430

XFAC_TI I 2.273746

ALPB_V I 1.204771

XFAC_V I 0.566891

ALPB_CR I 1.505878

XFAC_CR I 0.754833

ALPB_MN I 1.920970

XFAC_MN I 2.239969

ALPB_FE I 1.995455

XFAC_FE I 1.244120

ALPB_CO I 2.394155

XFAC_CO I 3.145732

ALPB_NI I 2.491283

XFAC_NI I 3.452112

ALPB_CU I 3.049738

XFAC_CU I 5.342329

ALPB_ZN I 1.785943

XFAC_ZN I 4.270507

ALPB_GA I 1.903558

XFAC_GA I 3.519264

ALPB_GE I 1.431330

XFAC_GE I 0.946363

ALPB_AS I 1.454624

XFAC_AS I 0.863506

ALPB_SE I 1.464103

XFAC_SE I 0.509254

ALPB_BR I 1.793757

XFAC_BR I 1.192163

ALPB_KR I 1.242469

XFAC_KR I 0.195416

ALPB_RB I 0.893509

XFAC_RB I 0.753057

ALPB_SR I 2.702289

XFAC_SR I 32.561240

ALPB_Y I 1.443236

XFAC_Y I 2.307839

ALPB_ZR I 1.402802

XFAC_ZR I 1.833851

ALPB_NB I 2.001333

XFAC_NB I 4.678302

ALPB_MO I 2.042051

XFAC_MO I 3.618240

ALPB_TC I 2.576693

XFAC_TC I 9.860653

ALPB_RU I 1.432008

XFAC_RU I 0.552218

ALPB_RH I 2.347687

XFAC_RH I 1.588054

ALPB_PD I 1.720521

XFAC_PD I 0.587118

ALPB_AG I 2.959757

XFAC_AG I 9.538157

ALPB_CD I 1.751947

XFAC_CD I 6.820820

ALPB_IN I 1.830626

XFAC_IN I 4.302750

ALPB_SN I 2.479003

XFAC_SN I 24.450811

ALPB_SB I 1.114193

XFAC_SB I 0.767547

ALPB_TE I 2.102109

XFAC_TE I 4.751442

ALPB_I I 1.619225

XFAC_I I 1.278518

USS XE -18.964330

UPP XE -108.181436

BETAS XE -2.718707

BETAP XE -44.936370

ZS XE 3.208788

ZP XE 2.727979

GSS XE 17.906443

GSP XE 4.106228

GPP XE 1.716979

GP2 XE 18.971469

HSP XE 4.990194

ALPB_H XE 1.356861

XFAC_H XE 0.701016

ALPB_HE XE 2.497832

XFAC_HE XE 2.599471

ALPB_LI XE 1.697716

XFAC_LI XE 4.467048

ALPB_BE XE 6.000011

XFAC_BE XE 0.654334

ALPB_B XE 3.233962

XFAC_B XE 1.995594

ALPB_C XE 1.704440

XFAC_C XE 0.826727

ALPB_N XE 1.932952

XFAC_N XE 0.925624

ALPB_O XE 2.566313

XFAC_O XE 1.623526

ALPB_F XE 2.837749

XFAC_F XE 2.086480

ALPB_NE XE 1.330202

XFAC_NE XE 0.293862

ALPB_NA XE 1.291138

XFAC_NA XE 5.076100

ALPB_MG XE 2.756089

XFAC_MG XE 9.774960

ALPB_AL XE 2.420691

XFAC_AL XE 7.358944

ALPB_SI XE 2.796986

XFAC_SI XE 16.526889

ALPB_CL XE 1.389615

XFAC_CL XE 0.593028

ALPB_AR XE 0.591520

XFAC_AR XE 0.049266

ALPB_K XE 0.886811

XFAC_K XE 1.526138

ALPB_CA XE 1.698890

XFAC_CA XE 2.050654

ALPB_BR XE 1.400900

XFAC_BR XE 0.711370

ALPB_KR XE 0.551561

XFAC_KR XE 0.049793

ALPB_RB XE 1.345397

XFAC_RB XE 1.856289

ALPB_I XE 1.187975

XFAC_I XE 0.555791

ALPB_XE XE 1.912510

XFAC_XE XE 9.565337

USS CS -3.996308

UPP CS -2.569885

BETAS CS -11.167340

BETAP CS 9.691485

ZS CS 1.776064

ZP CS 6.025310

GSS CS 18.164131

GSP CS 6.920824

GPP CS 16.792426

GP2 CS 8.175881

HSP CS 4.590034

ALPB_H CS 1.719572

XFAC_H CS 2.711386

ALPB_B CS 3.000034

XFAC_B CS 10.289233

ALPB_C CS 2.251416

XFAC_C CS 17.858749

ALPB_N CS 2.465681

XFAC_N CS 28.270100

ALPB_O CS 1.517551

XFAC_O CS 0.871027

ALPB_F CS 1.636155

XFAC_F CS 0.551707

ALPB_P CS 0.917812

XFAC_P CS 0.499881

ALPB_S CS 1.348833

XFAC_S CS 1.767711

ALPB_CL CS 1.241351

XFAC_CL CS 0.942491

ALPB_K CS 1.722882

XFAC_K CS 1.188658

ALPB_V CS 2.002665

XFAC_V CS 11.159719

ALPB_BR CS 1.949820

XFAC_BR CS 13.999636

ALPB_Y CS 0.929803

XFAC_Y CS 1.057035

ALPB_IN CS 0.852581

XFAC_IN CS 4.457697

ALPB_I CS 1.239277

XFAC_I CS 3.226708

ALPB_CS CS 1.267283

XFAC_CS CS 29.382256

USS BA -11.571532

UPP BA -9.917993

BETAS BA -10.914737

BETAP BA 9.727920

ZS BA 1.750490

ZP BA 1.968788

GSS BA 7.843618

GSP BA 19.900648

GPP BA 20.004643

GP2 BA 19.020523

HSP BA 0.979914

ALPB_H BA 3.120384

XFAC_H BA 27.058819

ALPB_C BA 1.318794

XFAC_C BA 0.549254

ALPB_N BA 2.188957

XFAC_N BA 4.679835

ALPB_O BA 2.337452

XFAC_O BA 4.174798

ALPB_F BA 2.539909

XFAC_F BA 3.008132

ALPB_MG BA 1.432600

XFAC_MG BA 10.342497

ALPB_AL BA 2.891358

XFAC_AL BA 15.460538

ALPB_SI BA 0.996995

XFAC_SI BA 0.887820

ALPB_P BA 1.646819

XFAC_P BA 8.719637

ALPB_S BA 1.637082

XFAC_S BA 1.742576

ALPB_CL BA 1.987384

XFAC_CL BA 2.636334

ALPB_CA BA 1.342035

XFAC_CA BA 2.833561

ALPB_TI BA 1.702345

XFAC_TI BA 4.943061

ALPB_CU BA 1.699850

XFAC_CU BA 1.896329

ALPB_BR BA 1.806723

XFAC_BR BA 2.830984

ALPB_SB BA 1.329425

XFAC_SB BA 12.262981

ALPB_I BA 1.370665

XFAC_I BA 2.112756

ALPB_BA BA 1.860576

XFAC_BA BA 57.199345

USS LA -15.586927

UPP LA 58.477136

UDD LA -19.818759

BETAS LA -18.460416

BETAP LA -19.708547

BETAD LA 0.849478

ZS LA 3.398968

ZP LA 1.811983

ZD LA 1.894574

ZSN LA 1.187188

ZPN LA 2.542482

ZDN LA 2.306744

GSS LA 4.516349

GSP LA 5.344112

GPP LA 10.610725

GP2 LA 9.202959

HSP LA 0.286106

POC LA 1.846287

F0SD LA 7.808849

G2SD LA 5.958952

ALPB_H LA 1.073406

XFAC_H LA 0.399521

ALPB_C LA 2.129683

XFAC_C LA 4.650201

ALPB_N LA 2.329214

XFAC_N LA 2.192625

ALPB_O LA 1.940554

XFAC_O LA 1.648001

ALPB_F LA 2.228378

XFAC_F LA 1.892928

ALPB_AL LA 1.003510

XFAC_AL LA 0.500540

ALPB_SI LA 2.872867

XFAC_SI LA 1.218295

ALPB_P LA 1.991054

XFAC_P LA 18.284518

ALPB_S LA 1.158196

XFAC_S LA 0.486832

ALPB_CL LA 1.835651

XFAC_CL LA 1.631876

ALPB_BR LA 1.253581

XFAC_BR LA 0.731795

ALPB_I LA 1.612519

XFAC_I LA 3.278712

ALPB_LA LA 2.066209

XFAC_LA LA 29.272376

ALPB_H GD 0.390870

XFAC_H GD 0.135810

ALPB_C GD 0.446870

XFAC_C GD 0.053040

ALPB_N GD 1.159410

XFAC_N GD 0.205050

ALPB_O GD 0.862040

XFAC_O GD 0.175800

ALPB_F GD 1.497980

XFAC_F GD 0.334630

ALPB_AL GD 1.003510

XFAC_AL GD 0.500540

ALPB_SI GD 2.112525

XFAC_SI GD 3.203995

ALPB_P GD 0.954450

XFAC_P GD 0.541660

ALPB_S GD 2.003930

XFAC_S GD 2.655400

ALPB_CL GD 0.806810

XFAC_CL GD 0.089970

ALPB_BR GD 0.715810

XFAC_BR GD 0.240740

ALPB_I GD 0.585360

XFAC_I GD 0.278240

ALPB_GD GD 3.348180

XFAC_GD GD 2.670400

USS LU -21.914035

UPP LU 54.132176

UDD LU -24.661582

BETAS LU -26.143720

BETAP LU -9.506888

BETAD LU 3.472080

ZS LU 2.327039

ZP LU 6.000335

ZD LU 1.208414

ZSN LU 0.449170

ZPN LU 2.469444

ZDN LU 2.216418

GSS LU 1.708751

GSP LU 2.037073

GPP LU 10.305910

GP2 LU 8.938585

HSP LU 0.000293

POC LU 5.824175

F0SD LU 9.700135

G2SD LU 6.013887

ALPB_H LU 2.089118

XFAC_H LU 7.421490

ALPB_C LU 2.386830

XFAC_C LU 6.432873

ALPB_N LU 1.647895

XFAC_N LU 0.783123

ALPB_O LU 1.889190

XFAC_O LU 0.868896

ALPB_F LU 1.888274

XFAC_F LU 1.730185

ALPB_P LU 1.345992

XFAC_P LU 8.048165

ALPB_CL LU 2.558367

XFAC_CL LU 8.330639

ALPB_BR LU 1.381701

XFAC_BR LU 0.992835

ALPB_I LU 1.436788

XFAC_I LU 4.313665

ALPB_LU LU 1.403653

XFAC_LU LU 21.889048

USS HF -25.690382

UPP HF -9.479410

UDD HF -39.077741

BETAS HF -4.866355

BETAP HF -21.264221

BETAD HF -12.878794

ZS HF 2.854938

ZP HF 3.079458

ZD HF 2.067146

ZSN HF 3.099683

ZPN HF 3.333027

ZDN HF 3.025020

GSS HF 11.791941

GSP HF 12.198754

GPP HF 13.909964

GP2 HF 12.064475

HSP HF 3.057466

F0SD HF 4.020384

G2SD HF 4.323408

ALPB_H HF 2.088799

XFAC_H HF 3.833288

ALPB_B HF 1.617370

XFAC_B HF 0.588837

ALPB_C HF 2.294622

XFAC_C HF 4.159075

ALPB_N HF 2.521801

XFAC_N HF 5.468404

ALPB_O HF 2.446232

XFAC_O HF 2.857484

ALPB_F HF 2.979096

XFAC_F HF 4.736067

ALPB_NA HF 1.840619

XFAC_NA HF 8.832085

ALPB_MG HF 1.911350

XFAC_MG HF 4.330250

ALPB_AL HF 0.949150

XFAC_AL HF 0.622520

ALPB_SI HF 2.189300

XFAC_SI HF 3.382300

ALPB_P HF 2.099591

XFAC_P HF 5.936976

ALPB_S HF 2.327110

XFAC_S HF 1.666760

ALPB_CL HF 1.953166

XFAC_CL HF 1.685929

ALPB_CA HF 2.054500

XFAC_CA HF 4.319510

ALPB_AS HF 1.799500

XFAC_AS HF 1.280820

ALPB_BR HF 2.237896

XFAC_BR HF 6.312154

ALPB_I HF 2.354639

XFAC_I HF 18.443532

ALPB_BA HF 2.264830

XFAC_BA HF 9.022520

ALPB_HF HF 2.216588

XFAC_HF HF 29.394192

USS TA -34.075891

UPP TA -5.504664

UDD TA -35.650460

BETAS TA -15.943219

BETAP TA 8.985389

BETAD TA -11.508162

ZS TA 4.116264

ZP TA 3.380936

ZD TA 1.755408

ZSN TA 1.011432

ZPN TA 2.139168

ZDN TA 1.685479

GSS TA 3.847731

GSP TA 4.550506

GPP TA 8.927545

GP2 TA 7.743093

HSP TA 0.256277

F0SD TA 7.257766

G2SD TA 1.619809

ALPB_H TA 1.786631

XFAC_H TA 1.893110

ALPB_C TA 1.450720

XFAC_C TA 0.581370

ALPB_N TA 2.013737

XFAC_N TA 1.152896

ALPB_O TA 2.494885

XFAC_O TA 2.316225

ALPB_F TA 2.732769

XFAC_F TA 2.163084

ALPB_NA TA 2.551120

XFAC_NA TA 8.276130

ALPB_P TA 2.513800

XFAC_P TA 6.261880

ALPB_S TA 2.091335

XFAC_S TA 3.201126

ALPB_CL TA 2.003584

XFAC_CL TA 1.661719

ALPB_K TA 4.521470

XFAC_K TA 2.026700

ALPB_BR TA 1.962327

XFAC_BR TA 3.310460

ALPB_I TA 1.500797

XFAC_I TA 1.995370

ALPB_TA TA 0.982767

XFAC_TA TA 0.831956

USS W -52.048404

UPP W -39.590059

UDD W -53.556920

BETAS W -63.148771

BETAP W -2.737119

BETAD W 1.132748

ZS W 3.881177

ZP W 2.044717

ZD W 1.928901

ZSN W 3.461491

ZPN W 1.904387

ZDN W 2.180340

GSS W 13.168346

GSP W 8.485482

GPP W 7.947717

GP2 W 6.893262

HSP W 0.826801

F0SD W 10.002668

G2SD W 3.417555

ALPB_H W 2.665390

XFAC_H W 5.441909

ALPB_C W 2.600118

XFAC_C W 4.729842

ALPB_N W 2.505903

XFAC_N W 3.877751

ALPB_O W 2.343168

XFAC_O W 1.878859

ALPB_F W 2.411756

XFAC_F W 1.368205

ALPB_NA W 1.090156

XFAC_NA W 0.686226

ALPB_MG W 1.434249

XFAC_MG W 1.904971

ALPB_P W 1.715627

XFAC_P W 4.472129

ALPB_S W 2.045564

XFAC_S W 2.401567

ALPB_CL W 1.907817

XFAC_CL W 1.349560

ALPB_K W 1.521243

XFAC_K W 2.096182

ALPB_CA W 1.870733

XFAC_CA W 8.590544

ALPB_FE W 1.787925

XFAC_FE W 1.977394

ALPB_NI W 1.775099

XFAC_NI W 1.430746

ALPB_ZN W 1.928464

XFAC_ZN W 5.376323

ALPB_BR W 2.143627

XFAC_BR W 3.993357

ALPB_RB W 0.900113

XFAC_RB W 4.075269

ALPB_ZR W 2.023641

XFAC_ZR W 19.994079

ALPB_I W 1.997307

XFAC_I W 5.825642

ALPB_CS W 0.899625

XFAC_CS W 4.061044

ALPB_BA W 1.566159

XFAC_BA W 1.861828

ALPB_W W 2.141401

XFAC_W W 13.807246

USS RE -41.679545

UPP RE 43.429894

UDD RE -54.761512

BETAS RE 8.467231

BETAP RE -6.468335

BETAD RE -11.136390

ZS RE 2.452162

ZP RE 1.583194

ZD RE 2.414839

ZSN RE 2.433415

ZPN RE 0.838026

ZDN RE 1.921708

GSS RE 9.257297

GSP RE 3.796290

GPP RE 3.497395

GP2 RE 3.033382

HSP RE 0.046330

F0SD RE 5.229236

G2SD RE 1.821985

ALPB_H RE 1.748317

XFAC_H RE 0.497281

ALPB_C RE 2.109510

XFAC_C RE 0.646616

ALPB_N RE 2.474230

XFAC_N RE 1.438570

ALPB_O RE 2.403640

XFAC_O RE 1.075351

ALPB_F RE 2.790322

XFAC_F RE 1.384171

ALPB_SI RE 2.775930

XFAC_SI RE 0.849450

ALPB_P RE 1.316878

XFAC_P RE 0.761808

ALPB_S RE 2.637193

XFAC_S RE 3.055234

ALPB_CL RE 2.857608

XFAC_CL RE 3.265852

ALPB_GE RE 2.852340

XFAC_GE RE 2.151580

ALPB_SE RE 2.523170

XFAC_SE RE 2.202140

ALPB_BR RE 2.195052

XFAC_BR RE 1.575571

ALPB_SB RE 2.204360

XFAC_SB RE 2.275780

ALPB_I RE 2.239594

XFAC_I RE 3.240592

ALPB_RE RE 2.195649

XFAC_RE RE 1.776660

USS OS -65.963764

UPP OS 37.736568

UDD OS -89.718816

BETAS OS -43.486712

BETAP OS -25.607006

BETAD OS -1.430819

ZS OS 3.094808

ZP OS 2.845232

ZD OS 1.986395

ZSN OS 2.613281

ZPN OS 2.062936

ZDN OS 2.944917

GSS OS 9.941551

GSP OS 8.617668

GPP OS 8.609401

GP2 OS 7.467158

HSP OS 1.886033

F0SD OS 8.758980

G2SD OS 4.717871

ALPB_H OS 2.399448

XFAC_H OS 3.609773

ALPB_C OS 1.938959

XFAC_C OS 0.616916

ALPB_N OS 2.139750

XFAC_N OS 0.730399

ALPB_O OS 2.539022

XFAC_O OS 1.230187

ALPB_F OS 2.210417

XFAC_F OS 0.562952

ALPB_NA OS 2.550740

XFAC_NA OS 8.275750

ALPB_P OS 2.060122

XFAC_P OS 4.267629

ALPB_S OS 2.809500

XFAC_S OS 4.186050

ALPB_CL OS 2.080978

XFAC_CL OS 1.177666

ALPB_K OS 1.351484

XFAC_K OS 0.875486

ALPB_BR OS 2.225810

XFAC_BR OS 2.709104

ALPB_I OS 2.189487

XFAC_I OS 4.869377

ALPB_OS OS 1.661052

XFAC_OS OS 0.928334

USS IR -40.856798

UPP IR -2.270208

UDD IR -68.020682

BETAS IR -11.770307

BETAP IR -13.487742

BETAD IR -5.642629

ZS IR 1.924564

ZP IR 3.510744

ZD IR 2.437796

ZSN IR 2.108777

ZPN IR 0.618406

ZDN IR 1.826929

GSS IR 8.022296

GSP IR 2.803574

GPP IR 2.580839

GP2 IR 2.238429

HSP IR 0.013100

F0SD IR 3.726074

G2SD IR 2.747207

ALPB_H IR 1.634365

XFAC_H IR 0.406470

ALPB_C IR 1.604977

XFAC_C IR 0.185955

ALPB_N IR 2.997358

XFAC_N IR 1.790021

ALPB_O IR 3.116069

XFAC_O IR 2.303902

ALPB_F IR 2.612609

XFAC_F IR 0.714245

ALPB_NA IR 2.550820

XFAC_NA IR 8.275830

ALPB_P IR 2.714060

XFAC_P IR 6.284670

ALPB_S IR 3.009199

XFAC_S IR 2.680449

ALPB_CL IR 2.575683

XFAC_CL IR 0.858848

ALPB_K IR 4.521170

XFAC_K IR 2.026400

ALPB_BR IR 2.058351

XFAC_BR IR 0.804901

ALPB_I IR 2.031222

XFAC_I IR 1.787121

ALPB_CS IR 1.559526

XFAC_CS IR 1.027369

ALPB_IR IR 1.465795

XFAC_IR IR 0.190914

USS PT -55.878758

UPP PT 52.660706

UDD PT -92.789895

BETAS PT -10.270452

BETAP PT 10.016048

BETAD PT -7.705919

ZS PT 2.922551

ZP PT 0.725689

ZD PT 2.158085

ZSN PT 3.083320

ZPN PT 19.427280

ZDN PT 2.233704

GSS PT 11.729692

GSP PT 13.983535

GPP PT 81.077279

GP2 PT 70.320441

HSP PT 0.000643

F0SD PT 4.725137

G2SD PT 3.459127

ALPB_H PT 3.062604

XFAC_H PT 2.051954

ALPB_C PT 2.296772

XFAC_C PT 0.370388

ALPB_N PT 2.347134

XFAC_N PT 0.447775

ALPB_O PT 2.680367

XFAC_O PT 0.827827

ALPB_F PT 3.157007

XFAC_F PT 1.031240

ALPB_AL PT 1.572360

XFAC_AL PT 1.056930

ALPB_SI PT 0.999990

XFAC_SI PT 0.099990

ALPB_P PT 1.307810

XFAC_P PT 0.485582

ALPB_S PT 2.919597

XFAC_S PT 2.008326

ALPB_CL PT 3.034813

XFAC_CL PT 1.610994

ALPB_K PT 1.495407

XFAC_K PT 2.058817

ALPB_BR PT 2.596546

XFAC_BR PT 1.409311

ALPB_AG PT 1.387422

XFAC_AG PT 5.456551

ALPB_I PT 2.228284

XFAC_I PT 1.174520

ALPB_PT PT 3.276872

XFAC_PT PT 8.178033

USS AU -94.841695

UPP AU -61.195249

UDD AU -114.242383

BETAS AU -13.460355

BETAP AU -24.921790

BETAD AU -63.835796

ZS AU 1.904923

ZP AU 2.408005

ZD AU 4.377691

ZSN AU 2.228930

ZPN AU 4.555019

ZDN AU 2.406645

GSS AU 8.479387

GSP AU 10.011584

GPP AU 19.009792

GP2 AU 16.487689

HSP AU 0.645273

F0SD AU 9.054233

G2SD AU 5.690708

ALPB_H AU 2.006469

XFAC_H AU 0.748516

ALPB_C AU 2.119485

XFAC_C AU 0.603200

ALPB_N AU 2.395362

XFAC_N AU 0.620935

ALPB_O AU 2.323131

XFAC_O AU 0.355344

ALPB_F AU 3.153884

XFAC_F AU 0.880186

ALPB_AL AU 1.572570

XFAC_AL AU 1.057140

ALPB_P AU 1.360881

XFAC_P AU 0.477023

ALPB_S AU 1.908644

XFAC_S AU 0.423252

ALPB_CL AU 2.495913

XFAC_CL AU 1.153029

ALPB_K AU 1.098797

XFAC_K AU 0.777289

ALPB_SE AU 1.840962

XFAC_SE AU 1.396436

ALPB_BR AU 1.633736

XFAC_BR AU 0.259683

ALPB_PD AU 1.311827

XFAC_PD AU 0.663311

ALPB_I AU 2.017017

XFAC_I AU 1.635086

ALPB_AU AU 1.539843

XFAC_AU AU 0.352184

USS HG -18.205464

UPP HG -15.280873

BETAS HG -9.919880

BETAP HG 2.134696

ZS HG 2.575831

ZP HG 1.955505

GSS HG 7.687301

GSP HG 8.355361

GPP HG 5.135379

GP2 HG 9.529761

HSP HG 0.954727

ALPB_H HG 1.953060

XFAC_H HG 3.306359

ALPB_C HG 1.702331

XFAC_C HG 0.911944

ALPB_N HG 1.715039

XFAC_N HG 1.016140

ALPB_O HG 2.151298

XFAC_O HG 2.032727

ALPB_F HG 1.836494

XFAC_F HG 0.631905

ALPB_NA HG 1.459803

XFAC_NA HG 2.437893

ALPB_SI HG 2.770860

XFAC_SI HG 3.680740

ALPB_P HG 0.891179

XFAC_P HG 1.351633

ALPB_S HG 1.900145

XFAC_S HG 1.772158

ALPB_CL HG 1.838378

XFAC_CL HG 1.142381

ALPB_TI HG 3.414630

XFAC_TI HG 2.957200

ALPB_SE HG 1.607270

XFAC_SE HG 1.015811

ALPB_BR HG 1.705395

XFAC_BR HG 1.997568

ALPB_TE HG 1.536568

XFAC_TE HG 4.486299

ALPB_I HG 1.476731

XFAC_I HG 2.489683

ALPB_HG HG 2.288223

XFAC_HG HG 29.334203

USS TL -31.112183

UPP TL -18.547083

UDD TL 10.294626

BETAS TL -2.456566

BETAP TL -4.949902

BETAD TL 0.079835

ZS TL 1.903342

ZP TL 2.838647

ZD TL 5.015677

GSS TL 11.438997

GSP TL 6.598450

GPP TL 6.054580

GP2 TL 5.507624

HSP TL 0.894757

ALPB_H TL 2.098110

XFAC_H TL 2.104104

ALPB_B TL 1.558857

XFAC_B TL 8.505888

ALPB_C TL 2.721075

XFAC_C TL 5.320930

ALPB_N TL 1.975560

XFAC_N TL 1.168533

ALPB_O TL 3.327926

XFAC_O TL 14.162059

ALPB_F TL 3.188782

XFAC_F TL 5.857103

ALPB_AL TL 1.458567

XFAC_AL TL 7.820754

ALPB_S TL 2.648560

XFAC_S TL 7.355971

ALPB_CL TL 3.127130

XFAC_CL TL 15.106797

ALPB_BR TL 2.596395

XFAC_BR TL 9.792163

ALPB_RB TL 1.512698

XFAC_RB TL 19.748653

ALPB_I TL 2.616072

XFAC_I TL 30.000873

ALPB_TL TL 2.597707

XFAC_TL TL 32.531404

USS PB -39.446347

UPP PB -29.348301

UDD PB -72.584748

BETAS PB -64.174888

BETAP PB -4.631384

BETAD PB -5.319005

ZS PB 4.706006

ZP PB 2.591455

GSS PB 8.368048

GSP PB 8.606930

GPP PB 6.431147

GP2 PB 6.550076

HSP PB 0.984819

ALPB_H PB 2.827636

XFAC_H PB 11.387111

ALPB_LI PB 0.947660

XFAC_LI PB 1.191773

ALPB_B PB 1.718658

XFAC_B PB 2.379671

ALPB_C PB 2.608618

XFAC_C PB 6.379969

ALPB_N PB 1.830414

XFAC_N PB 1.025862

ALPB_O PB 2.980453

XFAC_O PB 4.952015

ALPB_F PB 3.676637

XFAC_F PB 9.548494

ALPB_SI PB 1.284024

XFAC_SI PB 1.429987

ALPB_P PB 1.955648

XFAC_P PB 13.812691

ALPB_S PB 2.362854

XFAC_S PB 5.436090

ALPB_CL PB 1.499678

XFAC_CL PB 0.736101

ALPB_CA PB 1.691921

XFAC_CA PB 6.709141

ALPB_V PB 1.501708

XFAC_V PB 3.819119

ALPB_CR PB 1.305185

XFAC_CR PB 0.974428

ALPB_ZN PB 1.434109

XFAC_ZN PB 2.865002

ALPB_AS PB 1.792215

XFAC_AS PB 4.943875

ALPB_SE PB 2.893161

XFAC_SE PB 29.986812

ALPB_BR PB 2.364003

XFAC_BR PB 6.777519

ALPB_NB PB 1.500000

XFAC_NB PB 1.000000

ALPB_MO PB 1.759074

XFAC_MO PB 5.265939

ALPB_TE PB 3.242448

XFAC_TE PB 176.768383

ALPB_I PB 2.179090

XFAC_I PB 8.112077

ALPB_W PB 1.517042

XFAC_W PB 1.512242

ALPB_PB PB 2.529682

XFAC_PB PB 38.479040

USS BI -36.561343

UPP BI -30.823167

UDD BI -19.667431

BETAS BI -63.673960

BETAP BI -6.931981

BETAD BI -8.868066

ZS BI 5.465413

ZP BI 2.037481

ZD BI 2.855400

ZSN BI 4.275828

ZPN BI 3.018252

ZDN BI 4.889868

GSS BI 3.438678

GSP BI 3.987429

GPP BI 8.221978

GP2 BI 8.183927

HSP BI 1.610989

ALPB_H BI 1.727556

XFAC_H BI 1.225129

ALPB_LI BI 1.221685

XFAC_LI BI 2.187383

ALPB_C BI 1.970985

XFAC_C BI 1.397988

ALPB_N BI 1.976984

XFAC_N BI 1.315182

ALPB_O BI 2.337898

XFAC_O BI 1.621567

ALPB_F BI 2.029420

XFAC_F BI 0.490733

ALPB_NA BI 1.532800

XFAC_NA BI 2.410886

ALPB_S BI 1.866193

XFAC_S BI 1.624988

ALPB_CL BI 1.405944

XFAC_CL BI 0.496440

ALPB_K BI 1.417970

XFAC_K BI 2.123183

ALPB_SE BI 1.609528

XFAC_SE BI 1.139985

ALPB_BR BI 1.750597

XFAC_BR BI 1.792203

ALPB_RB BI 1.528441

XFAC_RB BI 2.435372

ALPB_I BI 1.592333

XFAC_I BI 2.364966

ALPB_CS BI 1.567880

XFAC_CS BI 2.314878

ALPB_BI BI 1.756620

XFAC_BI BI 7.710187
